# The mitochondrial genome map of *Nelumbo nucifera* reveals ancient evolutionary features

**DOI:** 10.1038/srep30158

**Published:** 2016-07-22

**Authors:** Songtao Gui, Zhihua Wu, Hongyuan Zhang, Yinzhen Zheng, Zhixuan Zhu, Dequan Liang, Yi Ding

**Affiliations:** 1 State Key Laboratory of Hybrid Rice, Department of Genetics, College of Life Sciences, Wuhan University, Wuhan 430072, China; 2 Nextomics Biosciences Co., Ltd., Wuhan, 430075, China

## Abstract

*Nelumbo nucifera* is an evolutionary relic from the Late Cretaceous period. Sequencing the *N. nucifera* mitochondrial genome is important for elucidating the evolutionary characteristics of basal eudicots. Here, the *N. nucifera* mitochondrial genome was sequenced using single molecule real-time sequencing technology (SMRT), and the mitochondrial genome map was constructed after *de novo* assembly and annotation. The results showed that the 524,797-bp *N. nucifera* mitochondrial genome has a total of 63 genes, including 40 protein-coding genes, three rRNA genes and 20 tRNA genes. Fifteen collinear gene clusters were conserved across different plant species. Approximately 700 RNA editing sites in the protein-coding genes were identified. Positively selected genes were identified with selection pressure analysis. Nineteen chloroplast-derived fragments were identified, and seven tRNAs were derived from the chloroplast. These results suggest that the *N. nucifera* mitochondrial genome retains evolutionarily conserved characteristics, including ancient gene content and gene clusters, high levels of RNA editing, and low levels of chloroplast-derived fragment insertions. As the first publicly available basal eudicot mitochondrial genome, the *N. nucifera* mitochondrial genome facilitates further analysis of the characteristics of basal eudicots and provides clues of the evolutionary trajectory from basal angiosperms to advanced eudicots.

The size of the mitochondrial genome differs among angiosperm species, ranging from approximately 220 kb (*Brassica napus*)^1^ to 11.3 Mb (*Silene conica*)^2^, reflecting the insertion of noncoding DNA^3^, including DNA of plastid and nuclear origin and DNA from horizontal gene transfer (HGT)^4^,^5^. Genomic organization is also variable across angiosperm evolution, reflecting frequent intramolecular or intermolecular homologous recombination mediated through repeats^6^. The organization, gene content and RNA editing of the mitochondrial genome differs over a significant scale across seed plants. This variation is useful for studying the evolution of both genome structures and sequences.

*Nelumbo nucifera* Gaertn. (Sacred lotus) is considered an evolutionary relic, which like *Ginkgo*, *Sequoia*, *Metasequoia* and *Liriodendron*, has survived since the Late Cretaceous period^7^. During the Early Cretaceous period, *N. nucifera* was a perennial aquatic plant that flourished during the middle Albian^8^,^9^. Currently, *N. nucifera* has been classified in the monotypic family Nelumbonaceae, which contains a single genus *Nelumbo.* This genus includes two species, *N. nucifera* and *N. lutea.* As a eudicot whose lineage emerged prior to the divergence of core eudicots^10^, *N. nucifera* provides new insights into the origin of eudicots. The nuclear^11^,^12^ and chloroplast^13^ genomes of *N. nucifera* have recently been released. However, no information on the *N. nucifera* mitochondrial genome has been reported. Thus, it is important to sequence the *N. nucifera* mitochondrial genome to reveal the evolutionary characteristics of this plant and provide clues concerning the evolutionary trajectory from basal angiosperms to advanced eudicots.

Third-generation sequencing through single molecule real-time sequencing technology (SMRT)^14^,^15^ produces considerably longer (up to 30 kb) unbiased DNA sequences without PCR amplification^16^. This technology has previously been used in *de novo* assembly through the PacBio RS II platform^17^,^18^,^19^,^20^,^21^.

In the present study, using an optimized method for mitochondrial DNA isolation, we prepared *N. nucifera* mitochondrial DNA and sequenced the genome using SMRT technology. The mitochondrial genome map was constructed after *de novo* assembly and annotation of the sequence data. Our analyses provide insights into the evolution of gene content and order, RNA editing patterns, positively selected sites and chloroplast DNA insertions in core eudicots.

## Results

### *N. nucifera* mitochondrial DNA isolation and genome assembly

Mitochondria were purified from *N. nucifera* etiolated seedlings after discontinuous sucrose gradient centrifugation and DNase I digestion. *Janus Green* B staining showed that most isolated mitochondria were intact (Supplementary Figure S1). The 260/230 and 260/280 ratios of isolated mtDNA were 2.08 and 1.93, respectively. Semi-quantitative PCR showed that the isolated DNA was pure enough to build a library for sequencing (Supplementary Figure S2).

PacBio RSII sequencing generated 76,495 reads (341,866,338-bp in total), with a mean read quality of 0.83. After trimming off adapters and low quality regions and correcting by mapping short reads to long seeds, we have obtained 9,165 reads (42,623,117-bp in total, 4,651-bp per read on average) with an accuracy of 99%. After filtering chloroplast reads, a total of 7,151 reads (31,112,098-bp in total, 4,351-bp per read on average) were used for the assembly process, reaching a coverage depth of 59 over the *N. nucifera* mitochondrial genome. The assembly was verified by comparing with Sanger sequencing of PCR amplification using 18 primer pairs. ABI3730 sequencing generated a total of 20,176-bp sequences, representing 3.84% of the genome. Only one mismatch was detected at position 68,132 of the assembled mitochondrial genome (Supplementary Table S1), making the assembly accuracy of approximately 99.995%.

### Genome size and content

The *N. nucifera* mitochondrial genome is assembled into a single circular-mapping^22^ molecule of 524,797-bp (Table 1), with a GC content of 48.16%. To our knowledge, *N. nucifera* has the second highest GC content of all plant mitochondrial genomes, while the *Butomus umbellatus* mitochondrial genome has the highest GC content of 49.1%^23^ (Supplementary Table S2). Eight long repeats (>500-bp) including four direct repeats (DRs) and four inverted repeats (IRs) were identified, accounting for 9.3% (48,898-bp) of the total size. In addition to the long repeats, the *N. nucifera* mitochondrial genome also contained many small repeats (20- to 500-bp), comprising 3.2% (16,668-bp) of the total length. Two hundred and one simple sequence repeats (SSRs) were identified (Supplementary Table S3), accounting for 0.5% (2628-bp) of the total length.

**Figure 1:**
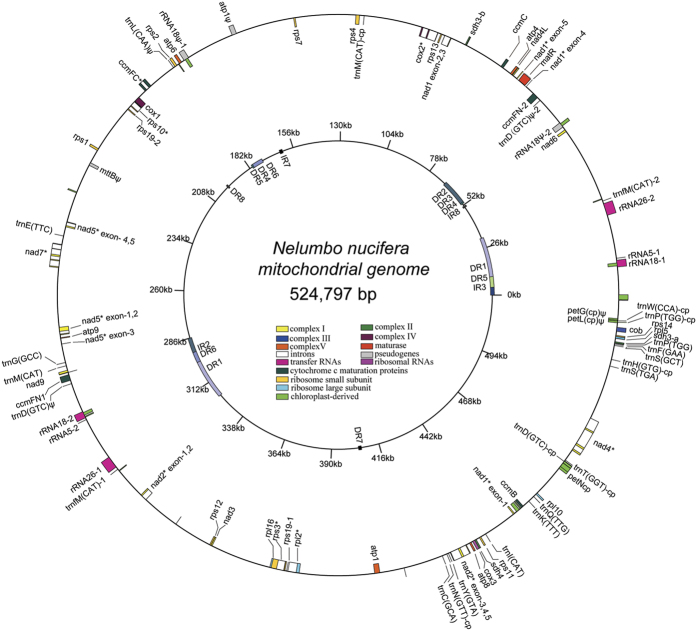
The *Nelumbo nucifera* mitochondrial genome map. Displayed as a circle, without implying that this is the *in vivo* conformation^22^. The genes are indicated in the outer circle, and the eight large repeats (>500-bp) with 99% identity are indicated in the inner circle. Chloroplast-derived regions are noted with a ‘-cp’ suffix. The same genes with different sequences are indicated with ‘-a,-b’. The same genes with the same sequences are indicated with ‘-1,-2′.

The *N. nucifera* mitochondrial genome contains a total of 63 genes, including 40 protein-coding genes, three rRNA genes (*rrn5*, *rrn18* and *rrn26*), 13 complete native mitochondrial tRNA genes and seven chloroplast-derived tRNA genes (Fig. 1, Table 2). Of these genes, *ccmFN, rps19* and all three rRNA genes have two identical copies, while *sdh3* has two different copies, *sdh3-*a and *sdh3-*b. *sdh3-*a is 39 bp longer than *sdh3-*b and has a 5-bp difference with *sdh3*-b at the 3′-terminal nucleotides (Supplementary Figure S3). Two different copies of a gene can result from horizontal gene transfer (HGT) events between angiosperm mitochondrial genomes^5^ or mtDNA recombination. However, the high sequence similarity (100% identity from 1 to 331 bases) of *sdh3-*a and *sdh3-*b suggests that these two copies result from *N. nucifera* mtDNA recombination rather than HGT from other species. Ninety-six unknown functional open reading frames (ORFs) were also predicted in the present study, comprising 7.3% (38,062-bp) of the total length (Table 1). The *N. nucifera* mitochondrial genome contained 25 Group II introns, including 20 *cis*-spliced and five *trans*-spliced introns. The total length of the 20 *cis*-spliced introns is 39,780-bp, accounting for 7.6% of the entire *N. nucifera* mitochondrial genome (Table 1),

To analyse the potential codon bias in the *N. nucifera* mitochondrial genome, RSCU (relative synonymous codon usage)^24^ was calculated using the coding sequences of 40 different mitochondrial protein-coding genes in *N. nucifera*. The results showed that A or U were more frequently used compared with G or C at the third position of *N. nucifera* mitochondrial codons (Supplementary Table S4), as observed in both chloroplast genomes and plant mitochondria genomes^25^,^26^. These results reflected a high A/T content at the third position of each codon^27^, that 61.27% of the *N. nucifera* mitochondrial codons have third position of A/T.

### Conserved gene clusters

Based on the endosymbiont hypothesis that mitochondrial genomes are the remnants of a free-living prokaryote (most likely an extant α-proteobacterium), some small DNA fractions of the original genes have been identified in the mitochondrial DNA of higher order plants^28^. Therefore, conserved gene clusters remain present in some angiosperms despite multipartite organizations in angiosperm mitochondrial genomes^29^,^30^. A comparison of the collinearity among 38 analysed species revealed 15 collinear gene clusters in the *N. nucifera* mitochondrial genome relative to *Liriodendron tulipifera* (Supplementary Table S5): *rrn18*-*rrn5*, *atp4*-*nad4L*, *atp8*-*cox3*-*sdh4*, *rpl2*-*rps19*-*rps3*-*rpl16*, *nad1*_exon2,3*-rps13*, *cox1-rps10*, *nad5*_exon4,5-*trnE*(*TTC*)-*nad7*, *nad5*_exon1,2-*atp9*, *nad3*-*rps12*, *trnN*(*GTT*)(cp)-*trnY*(*GTA*), *ccmB-trnK*(*TTT*)*-trnQ*(*TTG*), *trnS*(*GCT*)*-trnF*(*GAA*)*-trnP*(*TGG*)*-sdh3-*a, *cob-rps14-rpl5*, *trnP*(*TGG*)(cp)- *trnW*(*CCA*)(cp) and *petG*ψ-*petL*ψ. Among these, six *N. nucifera* mitochondrial gene clusters were found conserved in other angiosperm species. *Atp8-cox3-sdh4* occurred in both the ‘early-diverging’ angiosperm *L. tulipifera* and some eudicot species. *Cox1-rps10*, *nad5*_exon1,2*-atp9, trnN*(*GTT*)(cp)*-trnY*(*GTA*) and *petG*ψ-*petL*ψ were represented only in eudicots. *Nad5*_exon4,5*-trnE*(*TTC*)*-nad7* was found only in *L. tulipifera* and *N. nucifera*. The nine remaining gene clusters were variously distributed among all of these taxa.

### RNA editing of *N. nucifera* mitochondrial protein-coding genes

In the present study, with the exception of *rpl10*, all of the mitochondrial protein-coding genes of *N. nucifera* were successfully amplified, and a total of 33,719-bp cDNA sequences were sequenced; the amplicon for *ccmFC* was derived from pre-mRNA, with unspliced introns rather than mature mRNA. After aligning the DNA and cDNA sequences, 700 RNA editing sites were identified, including 612 nonsynonymous, 58 synonymous and 30 unpartitioned edits of the pseudogene *mttB* (Supplementary Table S6). As the same RNA editing sites were identified in both copies of *sdh3*, the RNA editing sites of the *sdh3* genes were only counted once. Approximately 87% of the total editing sites changed the translated amino acid sequence. Three U-to-C edits were identified in *ccmFN*, *cox1* and *cox2*, changing GUG (V) to GCG (A), UGC (C) to CGC (R) and UCU (S) to CCU (P), respectively. The most frequently edited genes were *atp9* and *ccmB*, with editing frequencies of 7.111 edits/100 nt and 6.924 edits/100 nt, respectively, while the frequencies of other genes were less than five edits/100 nt. The genes with no RNA editing sites included *ccmC*, *rps12*, *rps2* and *sdh4. N. nucifera* showed a pattern of relative editing levels across genes that was similar to other angiosperms^31^. For example, the ribosomal protein-coding genes had fewer edits, while the NADH dehydrogenase genes had more RNA edits.

In addition to RNA editing site analysis, we compared the RNA editing data obtained from *Amborella trichopoda*, *L. tulipifera*, *Vitis vinifera* and *Oryza sativa* with that of *N. nucifera* to identify similarities in the RNA editing sites (Supplementary Table S7). The results showed that the total number of RNA editing sites of *A. trichopoda, L. tulipifera* and *N. nucifera* (818, 802 and 700, respectively) were higher compared with *V. vinifera* and *O. sativa* (403 and 489, respectively). To examine similarities in RNA editing, we summarized the number of unique and shared edits in Supplementary Figure S4 using a pairwise comparison of the RNA editing sites of these five species. Compared with *A. trichopoda,* the maximum number of shared edits was 444 in *L. tulipifera*, followed by 361 in *N. nucifera,* 209 in *O. sativa* and 176 in *V. vinifera.* Compared with *L. tulipifera*, the maximum number of shared edits was 482 in *N. nucifera,* followed by 261 in *O. sativa* and 205 in *V. vinifera.* Moreover, *N. nucifera* contained the fewest number of unique edits (Supplementary Figure S4).

### Phylogenetic and selection pressure analysis

To examine the phylogenetic evolution of *N. nucifera* mitochondria, a maximum likelihood (ML) tree of 79 plant mitochondrial genomes, including *N. nucifera*, was built using the sequences of 41 mitochondrial protein-coding genes and rooted with the green algae *Mesostigma viride*^32^ (Supplementary Figure S5). The resulting tree showed that the topology was consistent with the representatives of the Angiosperm Phylogeny Group (APG) III^33^. In the phylogenetic tree, the algae were sisters of land plants; pteridophyte *Huperzia squarrosa* was a sister of the gymnosperm *Cycas taitungensis* and Angiospermae. Within the Angiospermae clade, *Amborella trichopoda* was sister of all other angiosperms. The monocots and the eudicots were monophyletic; *Spirodela polyrhiza* was sister of all other monocots; and *N. nucifera* formed an independent basal branch as a sister of all other eudicots. Each of these relationships has 100% bootstrap value support.

To explore potential horizontal gene transfer (HGT) events that occurred in *N. nucifera* mitochondria, Maximum Likelihood (ML) trees were reconstructed from 41 individual gene alignments using optimal substitution models (Supplementary Figure S6). The support of topological structure of each gene tree was evaluated by both bootstrap values of 1,000 bootstrap replicates and approximate likelihood ratio test (aLRT) values. The results showed that, among the 41 single gene trees, *N. nucifera* was located at the bottom of the angiosperm cluster as sister to the early-derived angiosperms *L. tulipifera* and *A. trichopoda* in 19 gene trees, and located as the basal branch of eudicot clusters in another 18 gene trees. In the rest four trees, *N. nucifera* was also embedded in the eudicot clusters: In *nad4L* and *rps19* trees, *N. nucifera* was sister to *V. vinifera*, which formed a basal branch of eudcot cluster in the mitochondria tree (Supplementary Figure S5). In *cox1* tree, *N. nucifera* was sister to *Vaccinium macrocarpon*, which was sister of all other Asterids in the mitochondria tree. And in *sdh4* tree, *N. nucifera* was sister to *Malus x domestica* which located at the bottom of Rosids cluster in the mitochondria tree. Many of the 41 gene trees had topologies inconsistent with the mitochondrial tree (Supplementary Figure S5) or the phylogenetic history of some plant species. For examples, *H. squarrosa* was embedded into the bryophytes cluster in 15 of all 33 gene trees containing *H. squarrosa.* And *Chara vulgaris* was clustered as sister to *Nitella hyalina* in all 32 gene trees containing *C. vulgaris*, 22 of which were well supported by both the bootstrap and aLRT values. Some eudicots were found among totally unrelated plant phyla in several gene trees: In *atp9* tree, *B. umbellatus* was clustered as sister to eudicots *Silene vulgaris* and *Silene latifolia* with bootstrap value of 85% and aLRT value of 0.89, but formed a long branch. In *nad7* tree, *V. macrocarpon* was embedded in the monocots cluster as sister to *Spirodela polyrhiza*. In *rps7* tree, *Salvia miltiorrhiza* and *Boea hygrometrica* were sisters to *Entransia fimbriata* with 100% bootstrap support and 100% aLRT support, but formed long branches. In *rps11* tree, *V. vinifera* was embedded into alga cluster. In *rpl2* tree, eudicots *S. miltiorrhiza*, *Ajuga reptans* and *Daucus carota* were clustered within alga branch. And in *sdh3* tree, eudicot *Millettia pinnata* was also embedded into alga cluster. Several observations may explain the cause of these tree abnormalities. The affinity of *S. miltiorrhiza* and *B. hygrometrica* with *E. fimbriata* in *rpl2* tree was more of a long branch attraction (LBA) of highly divergent sequences, because the alignment demonstrated that the *rps7* sequence of *E. fimbriata* was highly divergent from that of *S. miltiorrhiza* and *B. hygrometrica*. NCBI-BLASTN searches of these *rps7* genes against the Nucleotide Collection Database showed that *rps7* in *S. miltiorrhiza* and *B. hygrometrica* mitochondria have shown 100% identity with *rps7* of their own chloroplast genomes, while *rps7* in *E. fimbriata* showed low similarity with other nucleotides. The abnormalities in *rps11* tree and *rpl2* tree could also be caused by genes of chloroplast origin, because BLASTN showed a high similarly of *V. vinifera* mitochondrial *rps11* with its chloroplast *rps11,* the *rpl2* of *S. miltiorrhiza, A. reptans* and *D. carota* also showed 100% similarities with their chloroplast ones. The *sdh3* of *M. pinnata* showed extremely low similarity with records in both nucleotide collection database and non-redundant protein database of NCBI, which leads to the embedding of *M. pinnata* into alga cluster with a extremely long branch. The abnormalities in *nad7* tree may be caused by lacking of informative sites due to the limited sequence variations, since the deletion of *S. polyrhiza* and *Phoenix dactylifera* leads to the relocation of *V. macrocarpon* as sister to *D. carota* in *nad7* tree with higher branch support values. And the alignments showed that *V. macrocarpon* shows higher similarity with *D. carota* than with *S. polyrhiza*. The abnormalities in *atp9* tree may be also caused by lacking of informative sites considering its short length (225-bp in the alignments) and the high similarity between eudicots and monocots.

Mitochondrial protein-coding genes are essential for the assembly of respiratory chain complexes and the translation of proteins. Therefore, these genes are subject to purifying selection pressures^34^. The improved branch-site model was used to identify the positively selected sites of 41 mitochondrial genes in *N. nucifera* using the alignments and gene trees of the 79 mitochondria genomes. After likelihood ratio tests (LRT), alternative models were significantly favoured in 27 genes. However, in other genes, alternative models were not preferable to the null model (Supplementary Table S8). Positively selected sites were identified in 14 genes after Bayes Empirical Bayes (BEB) analyses. Among these 14 genes, only two genes had positively selected sites that were significantly supported by posterior probability values: 96.1% and 95.6% of the 25th and 31st sites in *sdh4*, respectively, and 99.6% of 36th site in *cox1*. The low proportion of positively selected genes in *N. nucifera* suggested that most of the *N. nucifera* mitochondrial genes were conserved and remained stable during evolution. The positively selected sites in *cox1* and *sdh4* might imply functional variations. Thus, the structural analysis and functional verification of these two genes will be necessary in subsequent research.

### Chloroplast DNA insertions in *N. nucifera* mtDNA

**Figure 2:**
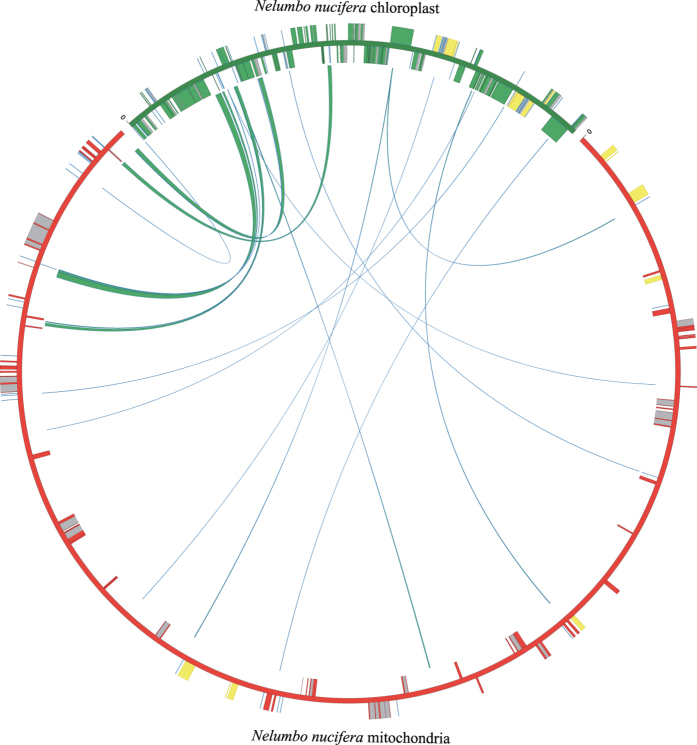
Schematic representation of chloroplast DNA transferred into the *Nelumbo nucifera* mitochondrial genome. The green line within the circle shows the regions of the plastid genome that has been inserted into different locations of the mitochondrial genome. The genes in plastid and mitochondria are indicated by green and red boxes, respectively. The introns are indicated by grey boxes, and rRNAs and tRNAs are indicated by yellow and blue boxes, respectively. The boxes on the inside and outside of the maps were transcribed in clockwise and counterclockwise directions, respectively.

Nineteen fragments of chloroplast DNA ranging from 54 to 1,998-bp were identified in *N. nucifera* mtDNA (Fig. 2, Table 3). The total size was 8,256-bp, approximately 1.6% of the *N. nucifera* mitochondrial genome and 5.0% of the chloroplast genome. After comparing the 19 chloroplast-derived fragments with the 78 other plant mitochondrial genomes used in the present study, homologues of 15 fragments were identified in other plant mtDNA (Supplementary Table S9). None of these chloroplast-derived fragments was observed in algae, bryophytes or the lycophyte *H. squarrosa*. The 141-bp fragment (positions 472035-472175) and the two shortest fragments (positions 91514-91569 and 342137-342190) were unique to the *N. nucifera* mitochondrial genome. The third shortest fragment (positions 413440-413496) was only identified in *A. trichopoda*. The longest fragment (positions 469237-471234) was only identified in *L. tulipifera*. The 84-, 81- and 78-bp fragments containing the *trnN*(GTT), *trnH*(GTG) and *trnM*(CAT) genes, respectively, had homologues in more than 40 species. The 84-bp fragment was commonly identified in angiosperms, but was absent in the gymnosperm *C. taitungensis*, while the 81- and 78-bp fragments were commonly observed in both angiosperms and *C. taitungensis*.

## Discussion

We sequenced the *N. nucifera* mitochondrial genome using third-generation SMRT sequencing technology and constructed a genomic map. In our previous work^13^, a comparison between *N. nucifera* chloroplast assemblies obtained using Sanger, Illumina MiSeq, and PacBio RS II platforms indicated that SMRT sequencing technology showed great promise for the production of highly accurate genome sequences because it produces long reads and lacks bias. In this study, the assembly validation experiment showed a great accuracy of *N. nucifera* mitochondrial genome assembly.

As the first publicly available basal eudicot mitochondrial genome, the *N. nucifera* mitochondrial genome provides valuable insights into the pattern of the evolution from basal angiosperms to advanced eudicots, and shows ancient evolutionary features in many aspects:

1. Conservation of genomic content. The *N. nucifera* mitochondrial genome retains 40 of all the 41 protein-coding genes present in ancestral flowering plant mitochondrial genomes^35^. This is consistent with previously described Southern blot results^36^, which suggest that the *Nelumbo* mitochondrial genome contains the full set of protein-coding genes present in the ancestral angiosperm. To our knowledge, of all available angiosperm mitochondrial genomes, only *A. trichopoda*^5^ and *L. tulipifera*^37^ have the full set of 41 protein-coding genes. Frequent loss and transfer of mitochondrial genes, particularly of the ribosomal protein-coding genes and succinate dehydrogenase (*sdh*) genes, has occurred in other angiosperm mitochondrial genomes and results in variable gene content among angiosperms^36^. The total length of the 20 *cis*-spliced introns of the *N. nucifera* mitochondrial genome is longer than any other sequenced angiosperm mitochondrial genome^37^. Currently, the complete set of 20 *cis*-spliced introns has only been identified in the mitochondria of three angiosperm species, including *N. nucifera* (in the present study), the ‘early-diverging’ angiosperm *L. tulipifera* and the ‘early-diverging’ core eudicot *Vitis vinifera.* In contrast, the five *trans*-spliced introns in *N. nucifera* were conserved in all of the sequenced angiosperm mitochondrial genomes.
2. Conservation of gene clusters. As a sister group, the ‘early-diverging’ angiosperm *L. tulipifera* occurred prior to the divergence of monocots and eudicots, showing primitive features in gene content and at RNA editing sites^37^. Hence, the *L. tulipifera* mitochondrial genome is a scale to explore the evolution of *N. nucifera* and other angiosperms. The *N. nucifera* mitochondrial genome has gene clusters that can date back to the original bacterial ancestor of mitochondria (*rrn18*-*rrn5*, *rpl2*-*rps19*-*rps3*-*rpl16*), gene clusters unique to angiosperms (*atp8*-*cox3*-*sdh4, nad1*_exon2,3 *-rps13*), a gene cluster unique to eudicots (*petG*ψ-*petL*ψ) and a gene cluster (*nad5*_exon4,5-*trnE*(*TTC*)-*nad7*) that is only shared with *L. tulipifera*. This further indicates that the *N. nucifera* mitochondrial genome was more primitive than other advanced angiosperms in angiosperm evolution.
3. Retention of ancient RNA editing sites. During the evolution of advanced angiosperms, RNA editing sites showed various degrees of subsequent losses in different lineages^37^,^38^,^39^. Though the emergence and maintenance of RNA editing in plants has not yet been clearly explained, a hypothesis falling under the scheme of “constructive neutral evolution” holds that editing may have emerged through neutral processes, to replace functionally important cytosines that require post-transcriptional editing to produce a conserved amino acid^40^. According to this hypothesis, most editing sites are nonsynonymous. In *N. nucifera*, approximately 87% of the total editing sites changed the translated amino acid sequence. Comparing RNA editing sites of *A. trichopoda*, *L. tulipifera*, *V. vinifera* and *O. sativa* with that of *N. nucifera* clearly shows that the degree of editing loss in *N. nucifera* is much lower than that in *V. vinifera* and *O. sativa*, suggesting that the *N. nucifera* mitochondrial genome was more ancient.

*N. nucifera* was originally considered to be the closest relative to *Nymphaea alba* because of morphological similarities, reflecting convergent evolution with *N. alba* in the same aquatic environment^41^. However, phylogenetic analyses of angiosperms based on their nuclear genome have revealed that *N. nucifera* is a member of the Proteales within the basal eudicots and phylogenetic analyses using chloroplast sequences clustered *N. nucifera* into the basal eudicot branches as a sister to Meliosma and Platanus^13^. In this study, a phylogenetic tree built using the sequences of 41 mitochondrial protein-coding genes also puts *N. nucifera* as a sister to all other eudicots. Finally, the phylogenetic analysis in the present study, along with previous studies based on chloroplast and nuclear genome data, provided a comprehensive view of the basal location of *N. nucifera* in eudicots. Thus, we concluded that *N. nucifera* is an ‘early-diverging’ basal eudicot. In the most of the 41 gene trees, the *N. nucifera* sequences were clustered in the basal location in eudicots, suggesting that *N. nucifera* mitochondrial genes maintained low evolutionary rates. Topological incongruence was observed in different species of many single gene trees in the present study. The embedding of *H. squarrosa into* the bryophytes clusters confirmed Liu’s study on *H. squarrosa* mitochondria^42^ which suggests the mitochondrial genome as the most archaic form in vascular plants and shows a gene content resembling those of charophytes and most bryophytes. *C. vulgaris* is considered to be the most closely related alga to land plants^43^. Turmel’s study on *C. vulgaris* mitochondria^43^ shows that *C. vulgaris* mtDNA strikingly resembles *Marchantia polymorpha* mtDNA, supported by both genome comparisons and phylogenetic analyses. The sister-group relationship between *C. vulgaris* and *N. hyalina* in our study suggested that *C. vulgaris* mtDNA may have more similarities with *N. hyalina* mtDNA. The embedding of species into totally unrelated plant phyla identified in six gene trees was expected to be possible HGT candidates. But our further analysis showed that this topological incongruence was caused either by long branch attraction of highly divergent genes, or by lacking of informative sites due to the limited sequence variations. Of the eight highly divergent genes, six genes were found similar to their own chloroplast genes, and three of these six genes were reported as chloroplast-derived by former studies (*rps11* of *V. vinifera*^6^, *rpl2* of *A. reptans*^44^ and *rpl2* of *D. carota*^4^). If assuming correct genome assembly and annotation, the low similarity of *sdh3* of *M. pinnata* mitochondria compared with other nucleotide in the NCBI nucleotide collection database may show some new insights. However, in the present study, we found none of the topological incongruences showed a reliable evidence of HGT event. And among the 41 gene trees, *N. nucifera* was clustered with either eudicots or the early-derived angiosperms, indicating that HGT from other plant mitochondria rarely occurred in *N. nucifera* mitochondrial genes.

However, intracellular gene transfer (IGT) of DNA from the plastid to the mitochondrial genomes of higher plants is a common phenomenon on an evolutionary timescale. Sometimes, these IGT events may initiate a gain of functional tRNAs. Due to the slow rate of mitochondrial sequence change, some of the chloroplast-derived fragments identified in *N. nucifera* mitochondria may trace back to retention of an earlier HGT event. The absence of the 19 chloroplast-derived fragments in algae, bryophytes and the lycophyte *H. squarrosa* indicates that DNA transfer from the chloroplast genome to the mitochondrial genome in *N. nucifera* might have occurred after the gymnosperm/angiosperm divergence. We found that the *trnN*(GTT)-contained chloroplast-derived fragment was absent in the gymnosperm *C. taitungensis* while the *trnH*(GTG)- and *trnM*(CAT)-containing fragments were commonly observed in both angiosperms and *C. taitungensis*. This finding is consistent with the analysis of chloroplast-derived tRNA gene evolution^37^. Thus, it is likely that the chloroplast-derived genes *trnH*(GTG) and *trnM*(CAT), previously present in *C. taitungensis*, were obtained from an earlier transfer, while *trnN*(GTT) might have occurred after the separation of gymnosperms and angiosperms. The short stretches of these three fragments in *N. nucifera* suggested that the selection of the flanking sequences might have occurred prior to the separation of early-diverging angiosperms and basal eudicots. Plant mitochondrial tRNAs were derived from three origins: (i) ‘native’ mitochondrial tRNAs, orthologous to those in moss mitochondria and derived from a mitochondrial ancestor; (ii) chloroplast-like mitochondrial tRNAs, highly homologous to chloroplast DNA; and (iii) unknown-origin mitochondrial tRNAs, such as the bacterial-like *trnC*(*GCA*), obtained through horizontal gene transfer events^45^,^46^. Compared to all sequenced angiosperm mitochondrial genomes, the *N. nucifera* mitochondrial genome had a complete set of 13 intact ‘native’ mitochondrial tRNAs, which were also identified in the ‘early-diverging’ core eudicot *V. vinifera,* but variously presented in other angiosperms. The ‘native’ mitochondrial tRNAs retained in gymnosperms, *trnL*(CAA) and *trnD*(GTC), have become pseudogenes in *N. nucifera* during the evolution of angiosperms^47^. The absence of *trnL*(CAA) and *trnD*(GTC) was compensated with chloroplast-derived *trnL*(CAA) or cytosolic tRNAs^3^. The *N. nucifera* mitochondrial genome contained seven chloroplast-derived tRNA genes present in the ‘early-diverging’ core eudicot *V. vinifera.* Notably, the chloroplast-derived *trnT*(GGT) gene included in the 141-bp unique fragment in *N. nucifera* was also identified within a 3,571-bp chloroplast-derived fragment in *V. vinifera* mitochondria^6^ but was absent in other plant mitochondrial genomes, suggesting that the gain of the chloroplast-derived *trnT*(GGT) gene in *N. nucifera* and *V. vinifera* mitochondria were separate events.

Though dozens of flowering plant mitochondrial genomes are published, early-derived angiosperms or basal eudicot mitochondrial genomes are still limited. The difficulty of isolating mitochondrial DNA is a bottleneck in acquiring complete mitochondrial genome sequence data. The optimized method for the isolation of mitochondrial DNA from plants rich in secondary metabolites used in the present study would promote the sequencing of more plant mitochondria. The genomic data of other basal eudicots, such as Platanus, will provide more detailed insights into mitochondrial genome evolution in basal eudicots.

## Materials and Methods

### Mitochondrial genome DNA isolation

Mature seeds of *N. nucifera* were harvested from the Wuhan Moshan Botanical Garden (114°22′ N, 30°33′ E) of Hubei Province, China and germinated in the dark at room temperature. Discontinuous sucrose gradient centrifugation combined with DNase I digestion *in vitro* was used to separate mitochondrial DNA from etiolated seedlings. Three pairs of primers specific to genes in the nuclear, mitochondria and chloroplast were designed to evaluate the purity of the mtDNA through semi-quantitative PCR. A full description of the mtDNA isolation method has been provided in Supplementary Method S1.

### Library construction and sequencing

A total of 20 μg of standard mtDNA was required to construct a size-selected ~20 kb SMRT-bell library. Subsequently, the SMRT-bell library was sequenced using three SMRT cells (Pacific Biosciences) with C2 chemistry on a PacBio RS II sequencing platform^14^. The sequencing and *de novo* assembly were performed at Yale University, Connecticut, America and Nextomics, Wuhan, China, respectively.

### Genome assembly and annotation

The primitive reads from the Pacbio RS II were filtered and corrected by mapping short reads to long “seed” reads (6,000-bp) using SMRT Analysis 2.1 (Filtering parameters: Minimum Subread Length = 500, Minimum Polymerase Read Quality = 0.8, and Minimum Polymerase Read Length = 100. Correcting parameters: Alignment Candidates Per Chunk = 10, Total Alignment Candidates = 24, Number Of Seed Read Chunks = 6, Blasr: -noSplitSubreads -minReadLength 200 -maxScore -1000 -maxLCPLength 16 ). Then, the reads were mapped to both the *Nelumbo nucifera* chloroplast genome [GenBank:KM655836] and the NCBI chloroplast genome database using Blat^48^ with default settings to filter chloroplast reads, reads with more than 90% matched and identity of more than 90% were removed. Both Celera Assembler 7.0^49^ and MIRA 4.0rc5 were used for the assembly process. Reads longer than 8,000-bp were extracted for assembly. In the Celera Assembler assembly process, the fastqToCA utility in Celera Assembler was used to generate wrapper LIB messages, reads were assembled into contigs using runCA (-p asm -d asm -s asm.spec) and then aligned to the contigs using the BWA-SW algorithm in BWA-0.7.3a^50^ with a mismatch penalty set to 5, contigs with less than 10X coverage depth were filtered. In the MIRA assembly process, the assembly was performed with parameters of “-GE:not = 20 –hirep_best -SK:mchr = 30 -HS:mnr = yes -HS:nrr = 5 PCBIOHQ_SETTINGS -CL:pec = no technology = PCBIOHQ”, redundancy was subsequently removed using self-to-self alignment performed by LastZ^51^. Both assembly processes output four contigs, these contigs were subsequently assembled into one contig using LastZ. At last, Quiver was used to correct the error regions and generate the best consensus sequence for the mitochondrial genome^16^. Default parameters were otherwise employed for processes above. To verify the assembly, 18 primer pairs were designed to target random selected intergenic regions (Supplementary Table S1).

The *N. nucifera* mitochondrial genome was manually annotated based on the output of NCBI-BLASTN and NCBI-BLASTX searches to a custom local database. The online tool tRNAscan-SE^52^ was also used to search the tRNA genes. Conserved ORFs were identified through BLASTX searches of the NCBI database. The mitochondrial genome map of *N. nucifera* was drawn using Genome Vx^53^. Large repeats (>500-bp) were identified through BLASTN searches with 99% identities. Chloroplast insertions were identified through BLASTN searches of the *N. nucifera* chloroplast genome with 70% identity and an e-value of 1e–5. To identify the nuclear-derived sequences, the protein-coding genes, cis-spliced introns, rDNAs, tRNAs and chloroplast-derived sequences were masked; subsequently, the masked sequences were searched in the *N. nucifera* nuclear genome. The RSCU (relative synonymous codon usage) of the *N. nucifera* mitochondrial genome was calculated from the coding sequences of 40 different protein-coding genes. MISA^54^ was used to screen for perfect SSRs, including mono-, di-, tri-, tetra-, penta- and hexa-nucleotide repeats. The minimum number of repeats was set at 10, 5, 4, 3, 3 and 3, respectively.

### Mitochondrial genome gene collinear analysis

To evaluate the collinear relationships between genes, all protein-coding genes, tRNA genes and rRNA genes from the *N. nucifera* mitochondria genome were compared to 37 representative species of angiosperm mitochondria genomes available in NCBI using BLASTP/BLASTX^55^. The blast results were inputted into MCscan^56^ with default parameters to compute multiple synteny. The final gene collinearity results were generated from the MCscan output file by swapping the gene order number of each gene with their names using a Perl script.

### RNA editing of *N. nucifera* mitochondrial protein-coding genes

Total RNA was isolated from tender leaves using the modified CTAB method^57^(Supplementary Figure S7, a); total cDNA was produced through RT-PCR (reverse transcription PCR) (Supplementary Figure S7,b). The genome sequence data were used to design PCR primers for all 40 protein-coding genes and one pseudogene (Supplementary Table S10). These primers were immediately flanked by genes based on the assumption that these regions comprised parts of the 5′ and 3′ untranslated regions, and the predicted PCR products were searched in the expressed sequence tags database using NCBI-BLASTN to evaluate whether the primers were in the untranslated regions. These genes were amplified from both the total DNA and total cDNA using the following conditions: 35 cycles of (40 s at 94 °C, 40 s at 48~56 °C and 1.5~2 min at 72 °C), with an initial step of 5 min at 94 °C and a final step of 10 min at 72 °C. The amplicons were detected on 1% agarose gels and purified using a Gel Extraction Kit (BioDev-Tech, Beijing, China). The purified cDNA amplicons were directly sequenced using an ABI 3730, and some of the purified products were also ligated into the pMD18-T Easy vector (TaKaRa, Dalian, China), transformed into Top10 chemically competent *E. coli* and subsequently sequenced. The sequenced cDNAs were compared with the genomic sequence and aligned using ClustalW^58^ implemented in MEGA5^24^. The editing sites were detected according to the alignments and the chromatogram peaks of the cDNA sequences and subsequently partitioned based on a nonsynonymous or synonymous effect using a Perl script. The RNA editing data of *A. trichopoda*^5^, *L. tulipifera*^37^, *O. sativa*^59^ and *V. vinifera*^6^ were acquired from GenBank or REDIdb and parsed using Perl scripts.

### Phylogenetic and selection pressure analysis

In addition to the sequenced mtDNA of *N. nucifera*, the protein-coding sequences from 78 published complete mitochondrial sequences of angiosperms were downloaded from the NCBI database (Supplementary Table S11). All 41 protein-coding genes were selected for phylogenetic analysis. These nucleotide sequences were separately aligned using ClustalW and manually adjusted, and the independent alignments were subsequently concatenated. The program jModelTest^60^ was used to identify the most appropriate substitution model and combination of gamma distribution and proportion of invariant sites, with the parameter number of rate categories set to 4. Both the Akaike Information Criterion (AIC) and Bayesian Information Criterion (BIC) were calculated. During the analysis, if the predicted models of AIC and BIC were not the same, AIC models were used (instead of BIC models). Phylogenetic analyses of each gene and the concatenated sequences were performed using the maximum likelihood (ML) method implemented in RAxML-HPC v8.1.2^61^ according to the optimal substitution models under the rapid bootstrap algorithm (1000 replicates). To test support for the branch points of each gene trees, nonparametric branch support tests based on the Shimodaira-Hasegawa-like approximate likelihood ratio test (SH-like aLRT) procedure were performed using phyML^62^ with the tree topology search operation option of Nearest Neighbour Interchange (NNI), and the same substitution models as used in RAxML trees. R packages ape^63^ and ggtree^64^ were used to combine the branch supports of bootstrap value with SH-like aLRT value in each gene tree and present the tree structures. The alignments can be found in Supplementary Data S1, the results of jModelTest and all the trees can be found in Supplementary Data S2.

The program Codeml in the PAML package^65^ was used to identify the positively selected genes in the *N. nucifera* mitochondrial genome. The phylogenetic trees of 41 protein-coding genes were used as tree structure files in Codeml, and *N. nucifera* was indicated as foreground in all gene trees. Test 2 of Model A in the branch-site model was selected, using the following parameters: in the null hypothesis, model  =  2, NSsites = 2, fix_omega = 1, omega = 1; in the alternative hypothesis, model = 2, NSsites = 2, fix_omega = 0, omega = 1.5; and other parameters were left by default. Likelihood ratio tests (LRT) were performed, and the P value was calculated using the chi2 program in PAML packages.

### Chloroplast insertions in *N. nucifera* mtDNA

After sequence annotation, the protein-coding genes, tRNA genes and rRNA genes of the *N. nucifera* mitochondrial genome were masked to eliminate BLAST hits of the conserved household genes in chloroplast and mitochondrial genomes. The masked genome sequence was BLASTed against the chloroplast genome of *N. nucifera*. The chloroplast-derived fragments were subsequently BLASTed against a local database containing all Viridiplantae mitochondrial genomes available in NCBI to identify homologues with an identity greater than 80% and full-length or nearly full-length (more than 90%) alignment. The maps of *N. nucifera* mitochondrial and chloroplast genomes and the chloroplast fragment transferences were drawn using Circos v.0.67^66^.

### Availability of supporting data

The precise mitochondrial genome of *N. nucifera* has been submitted to GenBank [GenBank: KR610474]. The raw sequence data have been deposited in the Short Read Archieve (SRA) database of NCBI [SRA: SRP066826], note that only data from SMRT cell 3 (named “mt_s3.fastq”) was used for the assembly in this study. Other data sets supporting the results of the present study are included within the article and supplementary information.

## Additional Information

**How to cite this article**: Gui, S. *et al.* The mitochondrial genome map of *Nelumbo nucifera* reveals ancient evolutionary features. *Sci. Rep.* **6**, 30158; doi: 10.1038/srep30158 (2016).

## Supplementary Material

Supplementary Information

Supplementary Data S1

Supplementary Data S2

## Figures and Tables

**Table 1:** The statistics of the features of the *Nelumbo nucifera* mitochondrial genome.

Category	Feature	Number	bp (%)
genome (524,797 bp)	G + C		252,718 (48.16)
Genes (total)			
	protein coding^1^	43	35651 (6.8)
	pseudogenes	9	5541 (1.1)
	rRNA^2^	6	11407 (2.2)
	mt-derived tRNA	13	1054 (0.2)
	cp-derived tRNA	7	506 (0.1)
	ORF	96	38062 (7.3)
Introns			
	cis-spliced	20	39780 (7.6)
	trans-spliced	5	n.a
Repeats			
	Repeats(>500)	8	48898 (9.3)
	Short Repeats(20<, <500)	191	16668 (3.2)
	SSRs	201	2628 (0.5)

^1^Each of gene *ccmFN, rps19* and *sdh3* has two copies.

^2^Each of the three ribosome RNAs (*rrn26, rrn18, rrn5*) has two copies.

**Table 2:** List of the genes present in the mitochondrial genome of *Nelumbo nucifera.*

Group of genes	Name of genes
Complex I	nad1*^1^, nad2*, nad3, nad4*, nad4L, nad5*, nad6, nad7*, nad9
Complex II	*sdh3-a, sdh3-b, sdh4*
Complex III	*cob*
Complex IV	*cox. cox2. cox3*
Complex V	*atp1. atp4. atp6. atp8. atp9*
Cytochrome c biogenesis	ccmB, ccmC, ccmFC*, ccmFN(×2)
Ribosome large subunit	rpl2*, rpl5, rpl10, rpl16
Ribosome small subunit	rps1. rps2, rps3*. rps4. rps7. rps10*, rps11,rps12, rps13, rps14, rps19 (×2)
Intron maturase	*matR*
rRNA genes	*rrn26*(×2)*, rrn18*(×2)*, rrn5*(×2)
tRNA genes	*trnC*(*GCA*)*, trnE*(*TTC*)*, trnG*(*GCC*)*, trnK*(*TTT*)*, trnM*(*CAT*)*, trnfM*(*CAT*)(×2)*, trnI*(*CAT*)*, trnF*(*GAA*)*, trnP*(*TGG*)*, trnQ*(*TTG*)*, trnS*(*GCT*)*, trnS*(*TGA*)*, trnY*(*GTA*)
chloroplast-derived genes	*petN*(*cp*)*, trnM*(*CAT*)*-cp, trnN*(*GTT*)*-cp, trnD*(*GTC*)*-cp, trnT*(*GGT*)*-cp, trnH*(*GTG*)*-cp, trnP*(*TGG*)*-cp, trnW*(*CCA*)*-cp*
Pseudogenes	*atp1ψ, mttBψ, petG*(*cp*)*ψ, petL*(*cp*)*ψ, ycf3*(*cp*)*ψ, rRNA18-1ψ, rRNA18-2ψ, trnD*(*GTC*)*ψ, trnL*(*CAA*)*ψ*

^1^Genes with introns are indicated with asterisks (*).

**Table 3:** Chloroplast Insertions in the *Nelumbo nucifera* mitochondrial genome.

No.	Length	Position	Genes Contained
1	1998	469237–471234	***petN;psbM***
2	1387	523411–524797	*ycf3*
3	1229	516533–517761	*petL-petG-**trnW***(***CCA***)***-trnP***(***UGG***)
4	997	451200–452196	*psbC*
5	621	471275–471895	***trnD***(***GTC***)
6	321	230963–231283	*psbC*
7	297	452532–452828	*psbD;psbC*
8	211	181488–181698	*ndhD*
9	206	29058–29263	*ycf2*
10	206	318795–319000	*ycf2*
11	145	523258–523402	*ycf3*
12	141	472035–472175	***trnT***(***GGT***)
13	87	285719–285805	*ycf2*
14	84	426801–426884	***trnN***(***GTT***)
15	81	505480–505560	***trnH***(***GTG***)
16	78	123420–123497	***trnM***(***CAT***)
17	57	413440–413496	*ndhE*
18	56	91514–91569	*trnT*(*GGT*)
19	54	342137–342190	*trnA*(*TGC*)

Pseudogenes were shown in regular font. Genes with intact ORFs and genes which encode tRNAs with intact folding ability are highlighted in bold.
