# Supplementary material for: The mitochondrial genome map of Nelumbo nucifera reveals ancient evolutionary features
Source: Sci Rep. 2016 Jul 22;6:30158. doi: 10.1038/srep30158 (PMC4957087; doi:10.1038/srep30158)
Supplement: Supplementary Information [file srep30158-s1.pdf]

The mitochondrial genome map of *Nelumbo nucifera* reveals ancient evolutionary features

Songtao Gui[1#], Zhihua Wu[1#], Hongyuan Zhang[1], Yinzhen Zheng[1], Zhixuan Zhu[1], Dequan Liang[2] and Yi Ding[1*]

1 State Key Laboratory of Hybrid Rice, Department of Genetics, College of Life Sciences, Wuhan University, Wuhan 430072, China

2 Nextomics Biosciences Co., Ltd., Wuhan, 430075, China

#The authors contributed equally to this study

*Correspondence and requests for materials should be addressed to Y D.(email: yiding@whu.edu.cn)

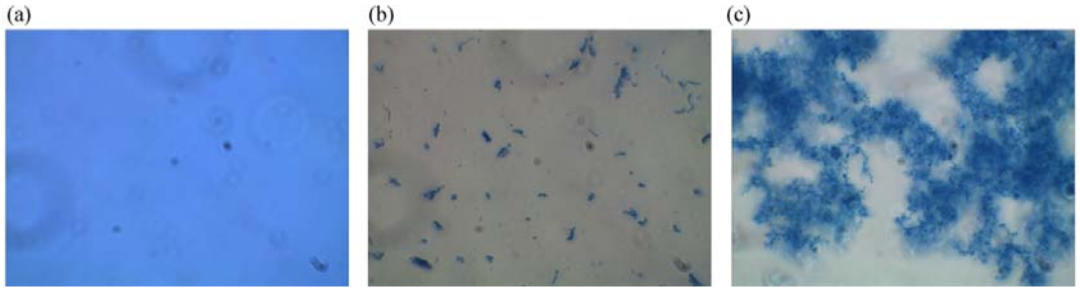

Figure S1. The microscopic observation of dyed mitochondria using *Janus Green* B staining. (a) Native control of water, (b) positive control of oral epithelial cells and (c) isolated *N. nucifera* mitochondria with activity.

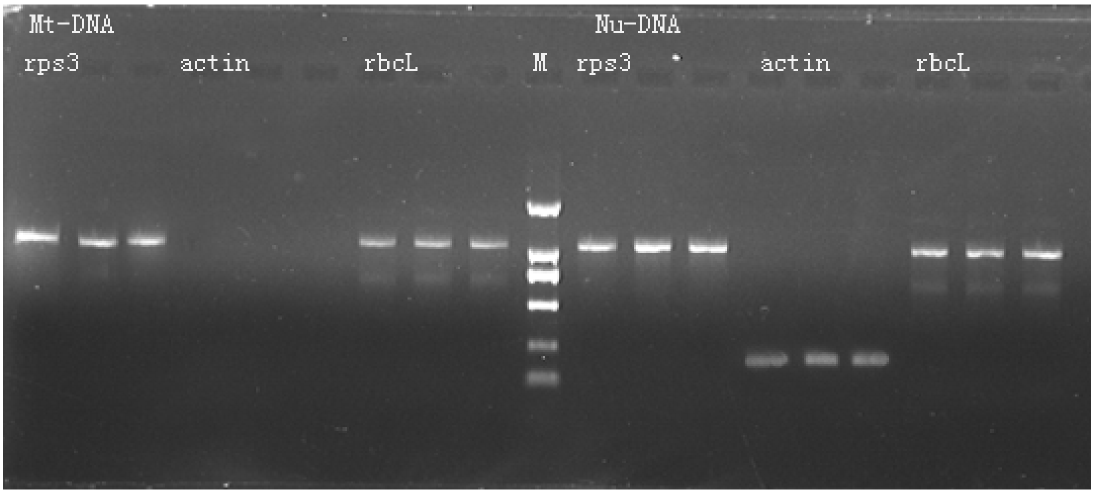

Figure S2. The detection of mtDNA purity using three specific primers from nuclear (*β-actin*), mitochondrial (*rps3*) and chloroplast (*rbcL*) genes. "mt-DNA" represents the mtDNA isolated from the 1.6 M/1.2 M sucrose interface, and "Nu-DNA" represents the total DNA. "M" indicates the DL2000 marker.

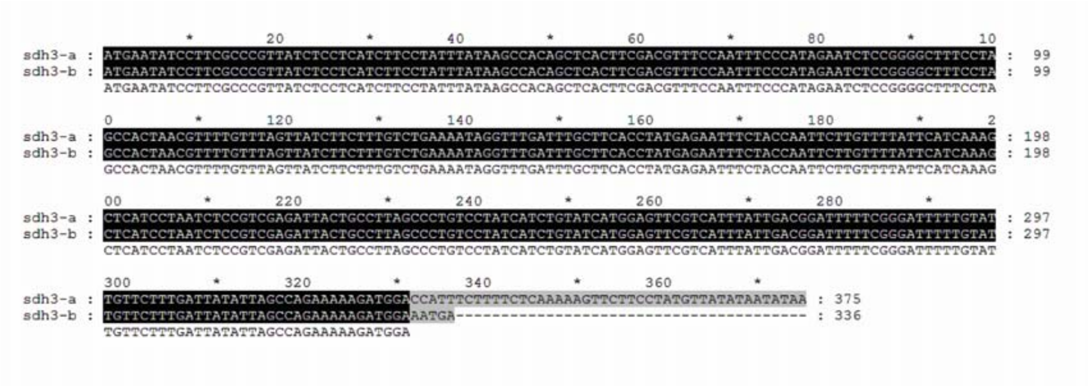

Figure S3. The alignment of the *sdh3-a* and *sdh3-b* genes in the *N. nucifera* mitochondrial genome.

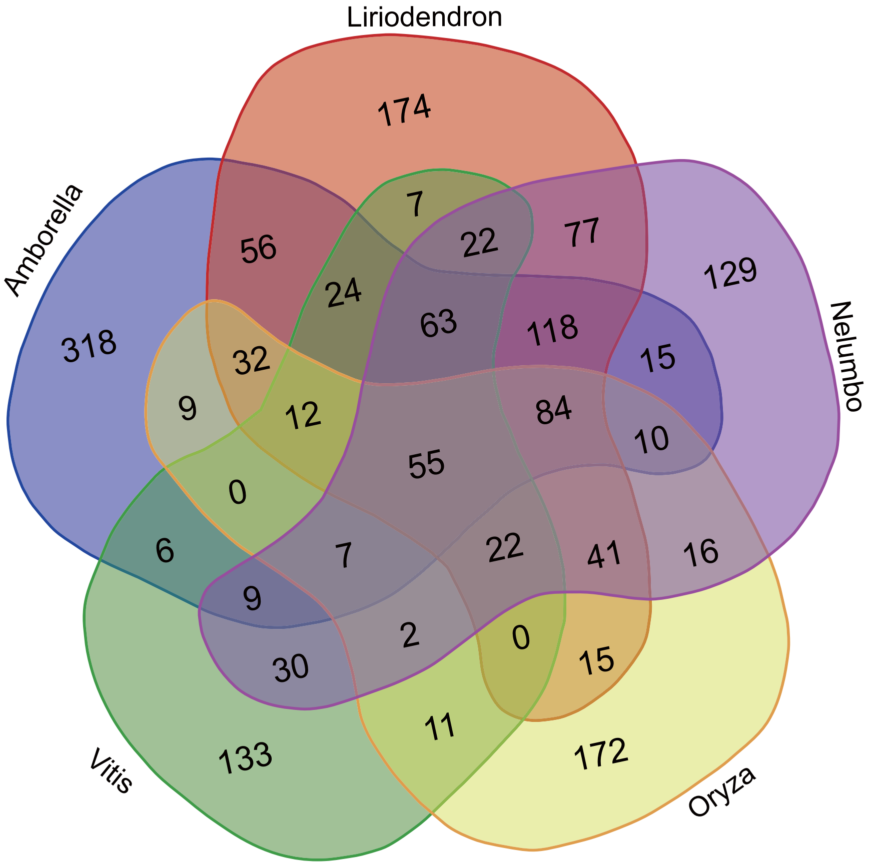

Figure S4. Venn diagram of the shared RNA editing sites among *Amborella trichopoda, Liriodendron tulipifera*, *Nelumbo nucifera*, *Vitis vinifera* and *Oryza sativa*.

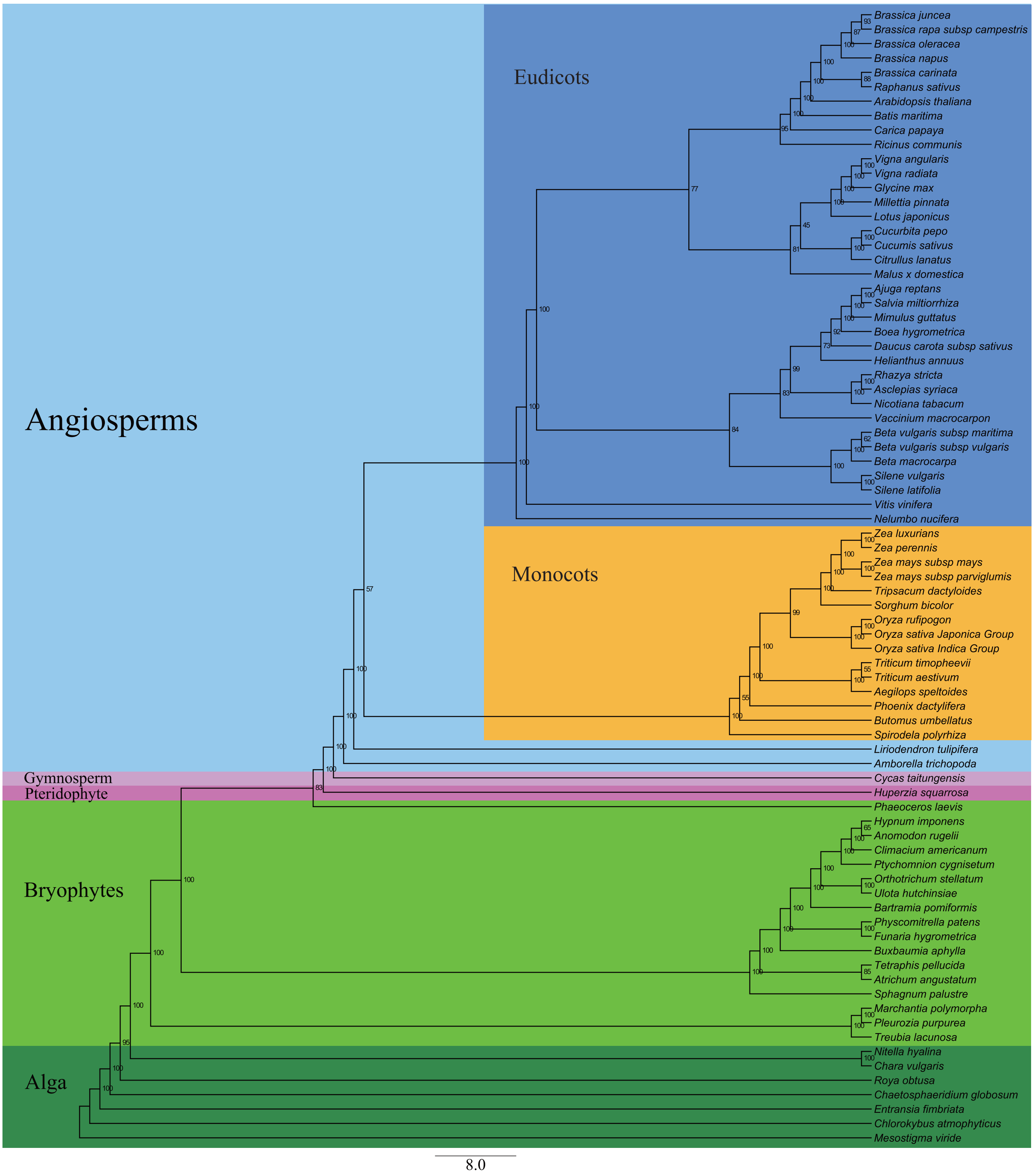

Figure S5. Phylogenetic tree constructed using the data from 79 taxa based on 41 mitochondrial protein-coding genes. Different phyla and classes are highlighted in different colors, with the name of each phylum or class marked at the left of each highlight. The number at each node indicates the percentage of bootstrap value.

Figure S6. The maximum likelihood trees of 41 single genes. Bootstrap values >50 or aLRT values >0.75 are shown on the branches. Black dots on the node indicate bootstrap value >60 and aLRT values >0.8; red dots on the node indicate bootstrap value >85 and aLRT values >0.95. Different phyla and classes are highlighted in different colors (Alga, dark green; Bryophytes, light green; Pteridophyte, purple; Gymnosperm, pink; Angiosperms, light blue; Monocots, orange; Eudicots, dark blue). The branch scales (substitutions per site) are shown on the top left of each tree, bold branches correspond to four times the scales.

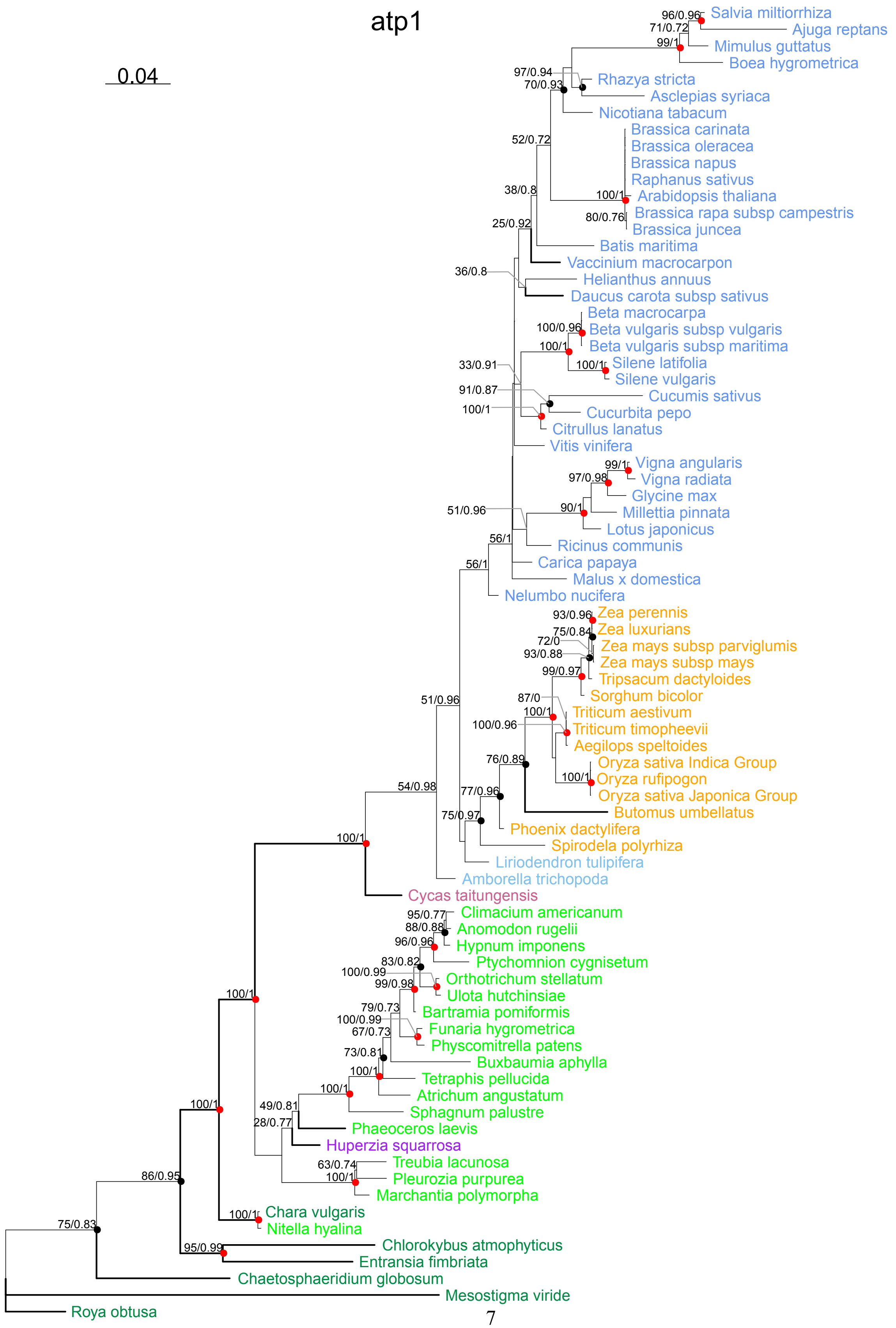

Figure S6 (continued)

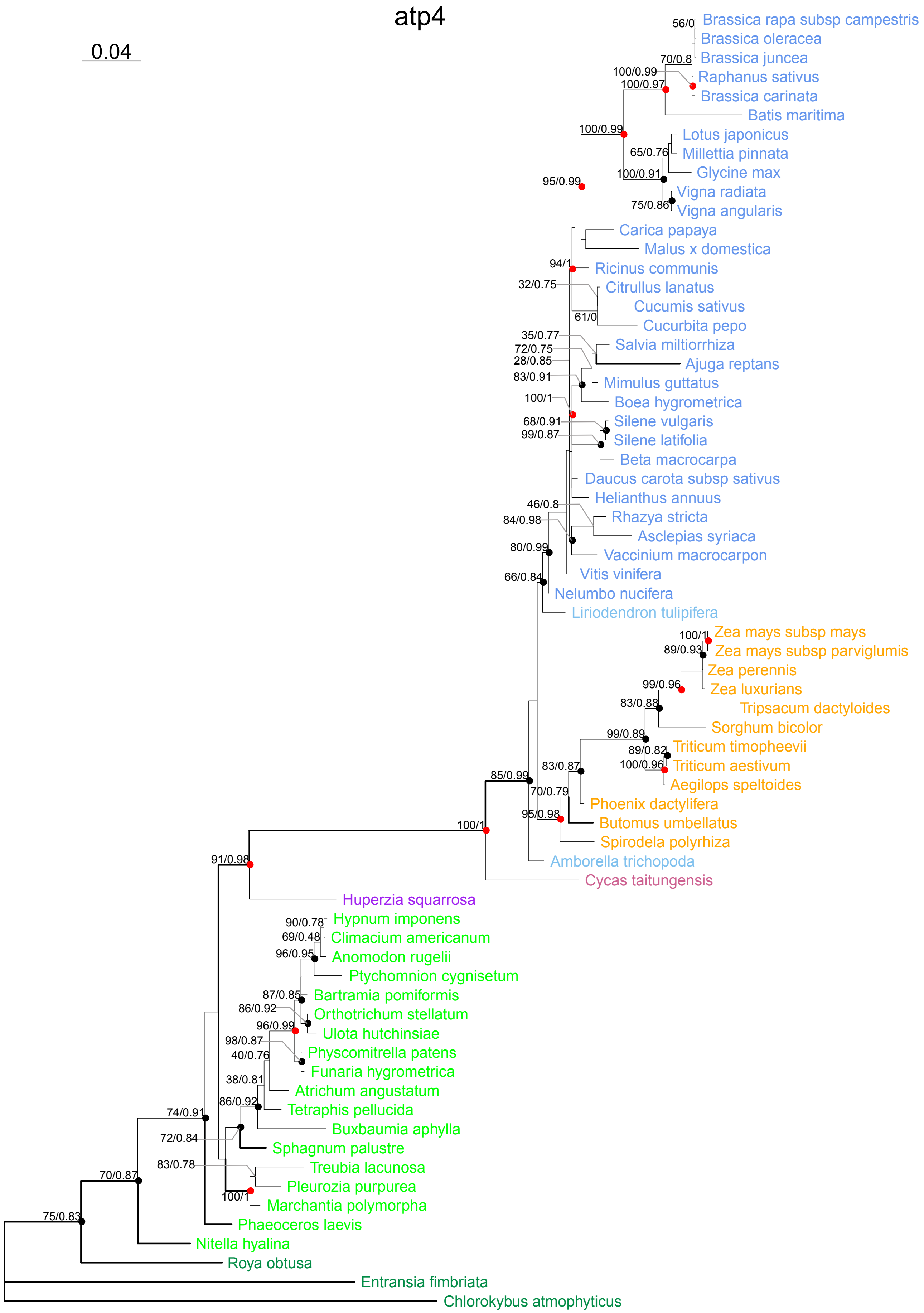

Figure S6 (continued)

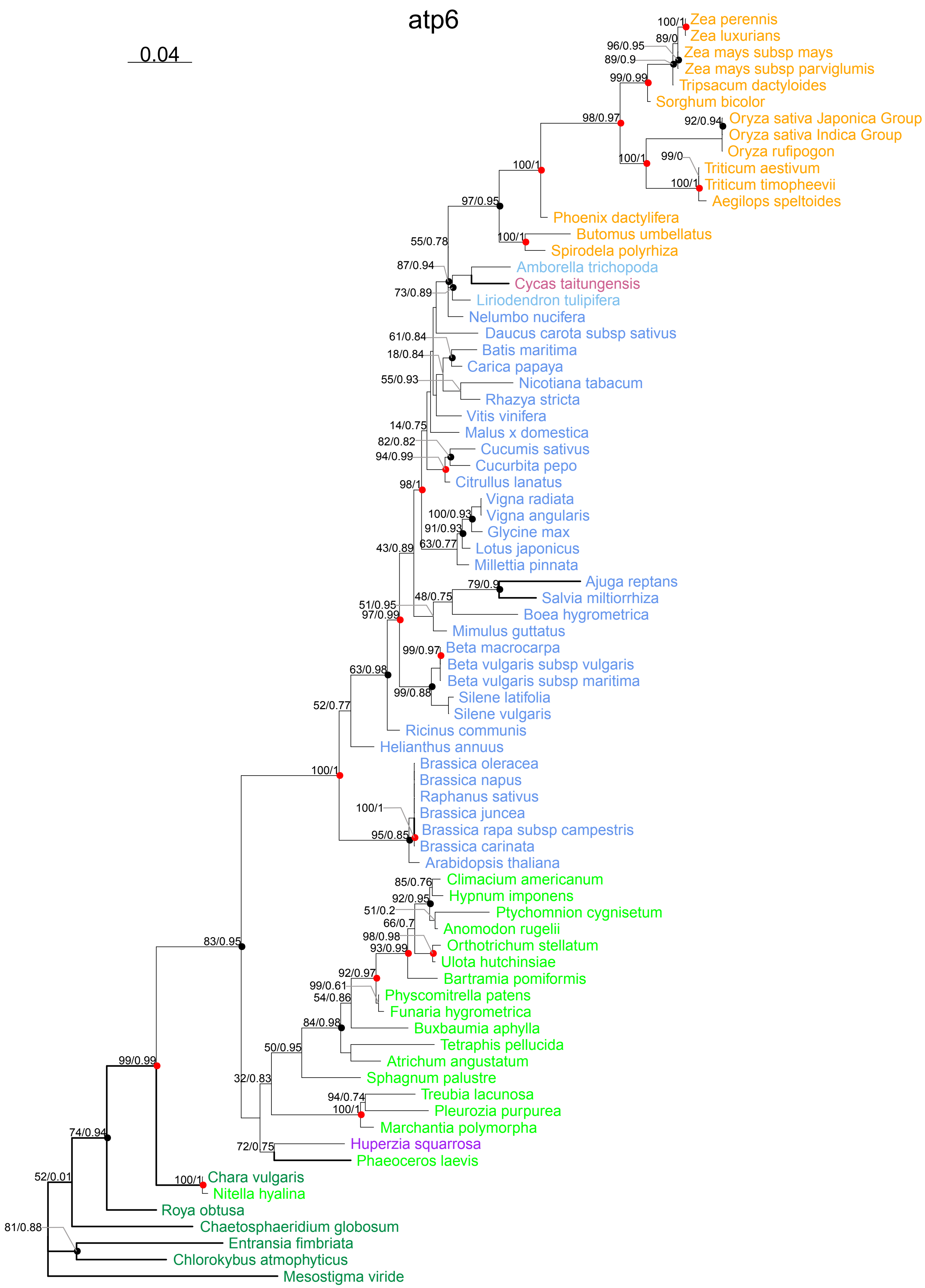

Figure S6 (continued)

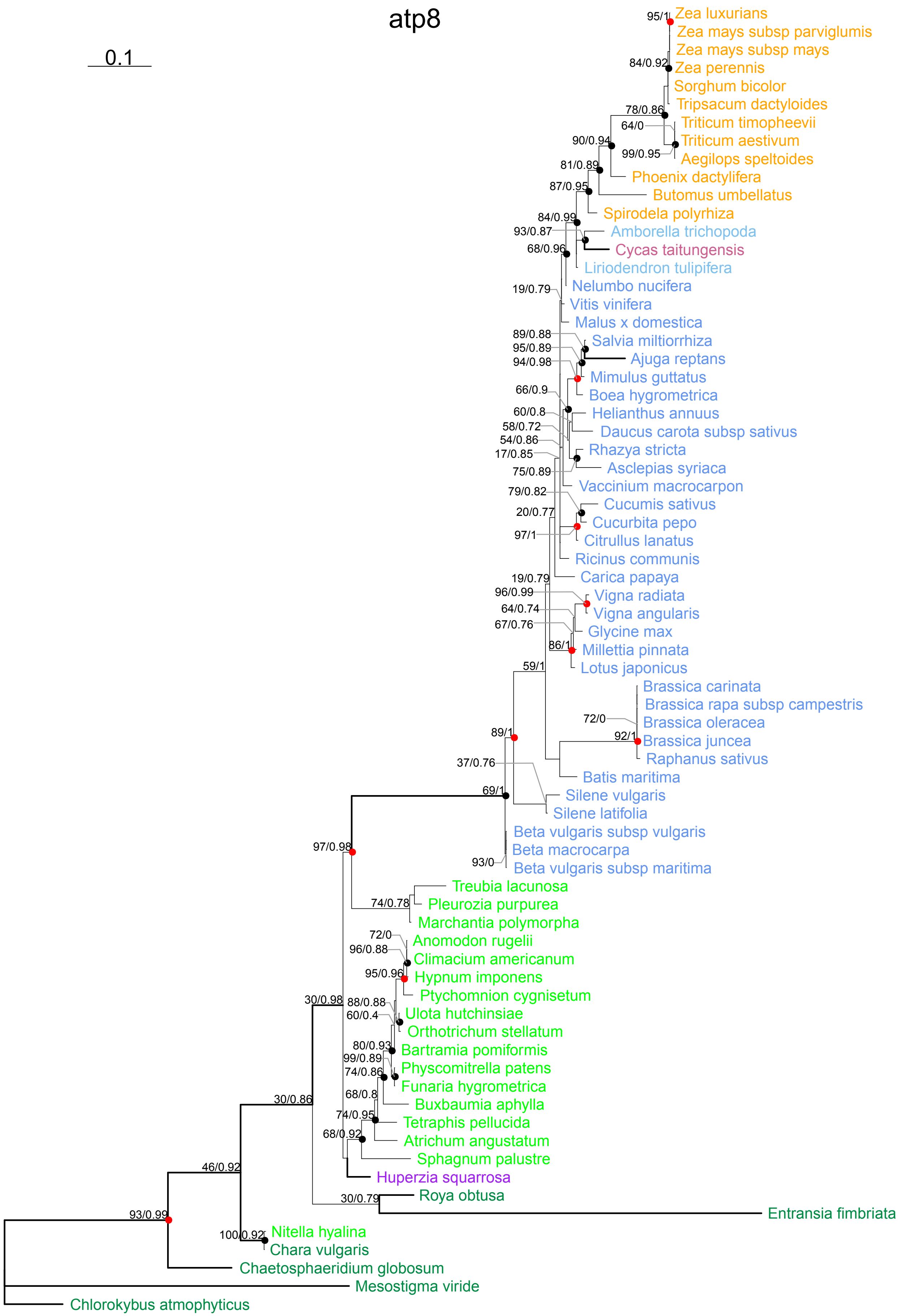

Figure S6 (continued)

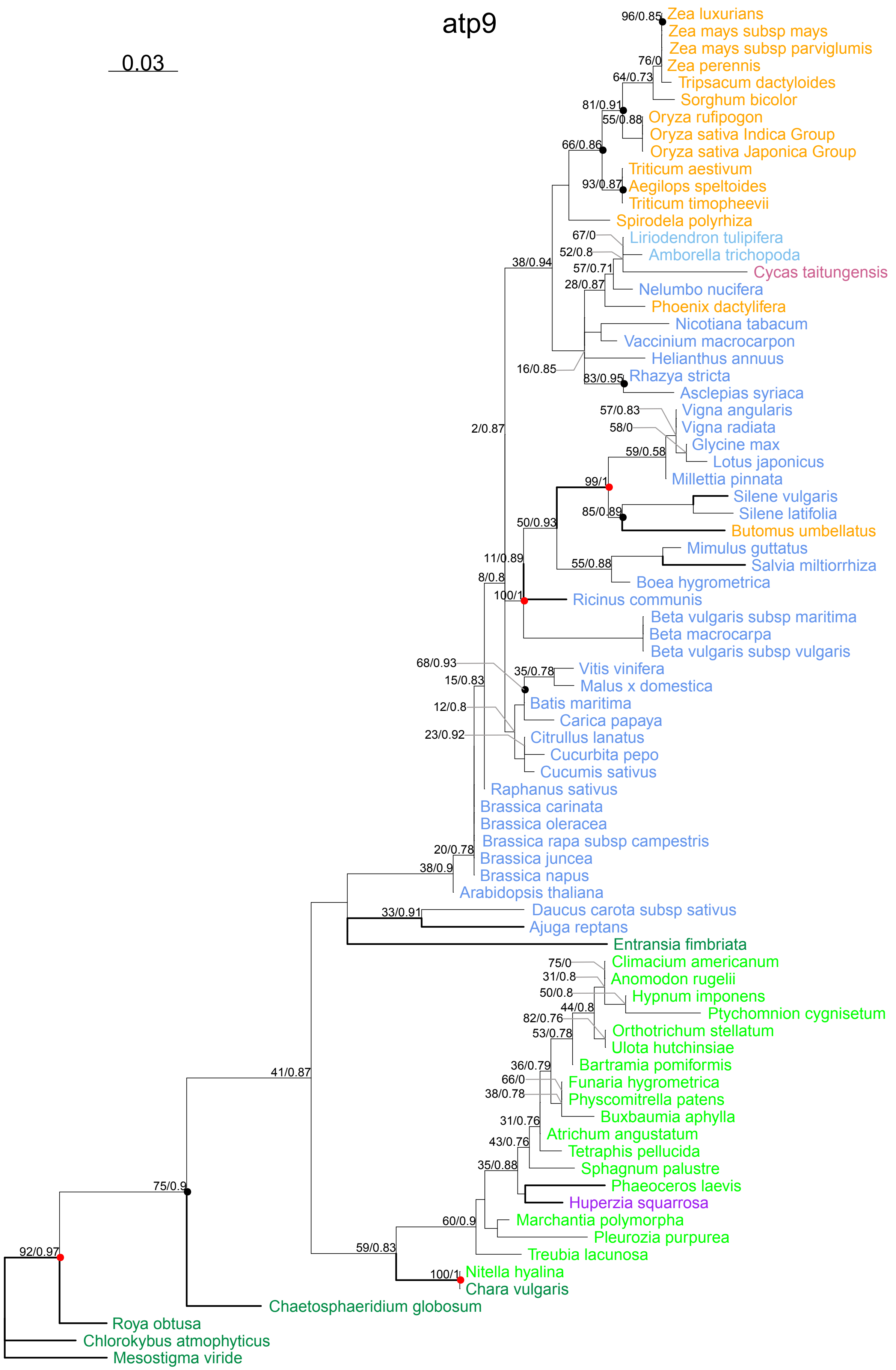

Figure S6 (continued)

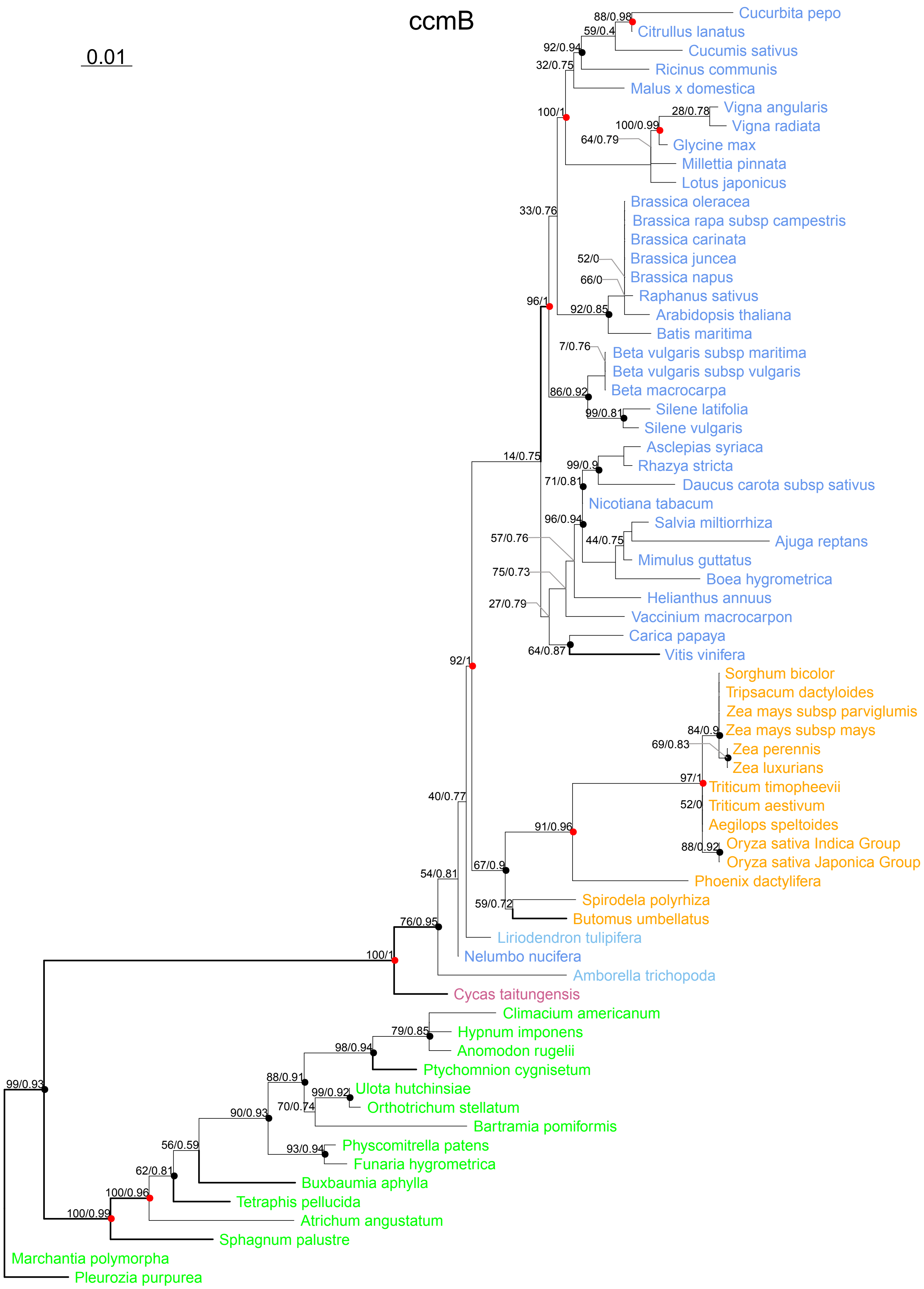

Figure S6 (continued)

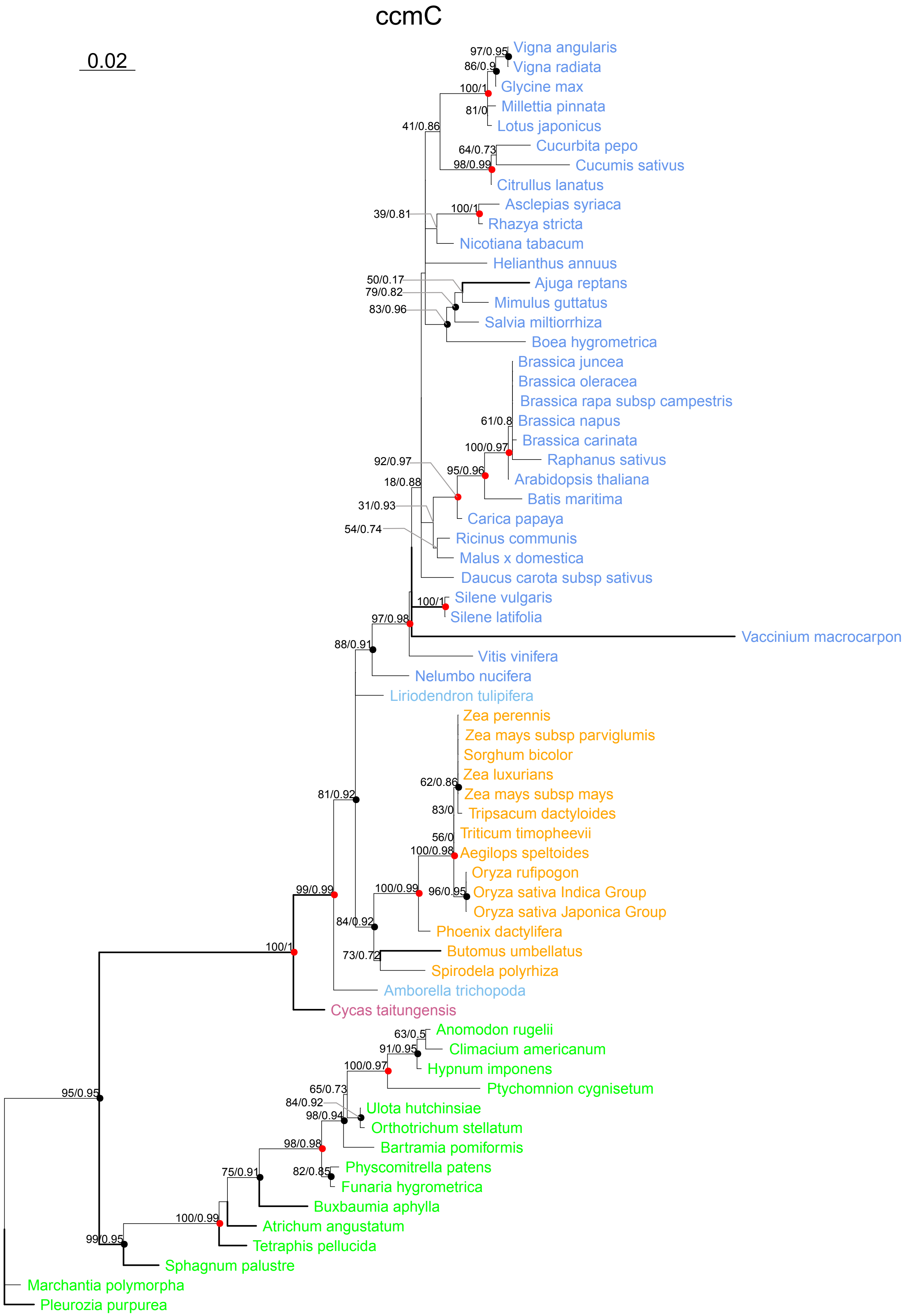

Figure S6 (continued)

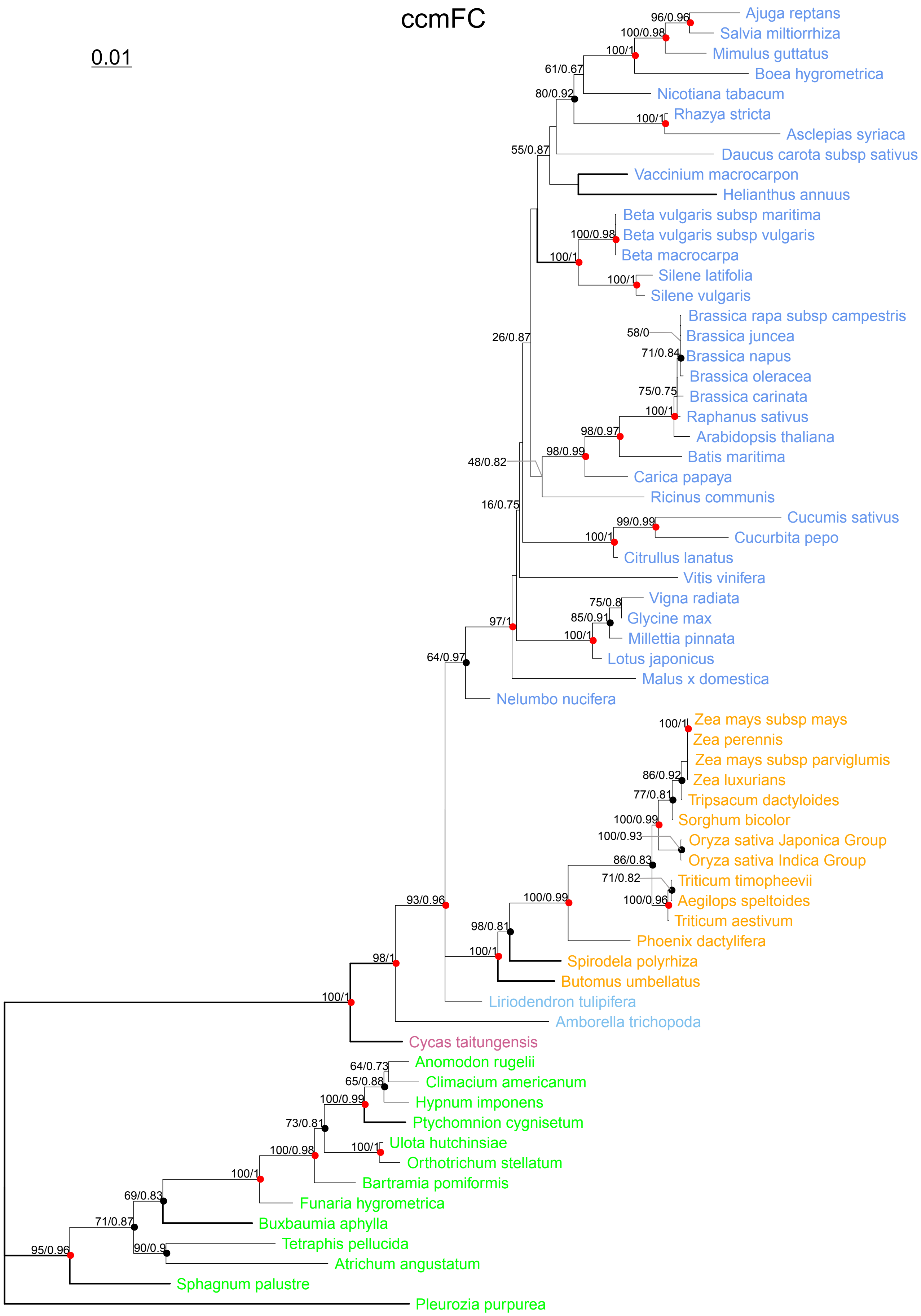

Figure S6 (continued)

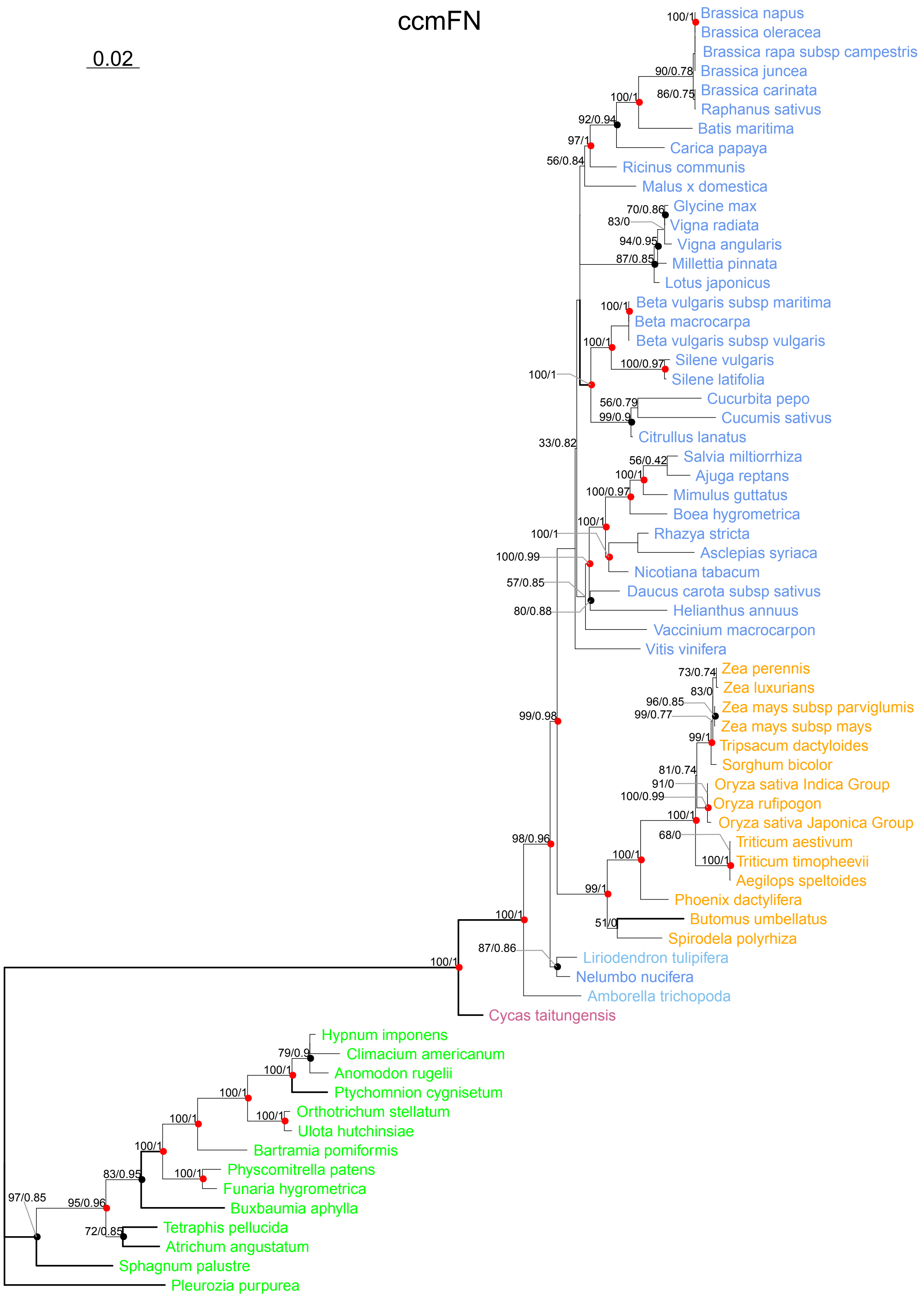

Figure S6 (continued)

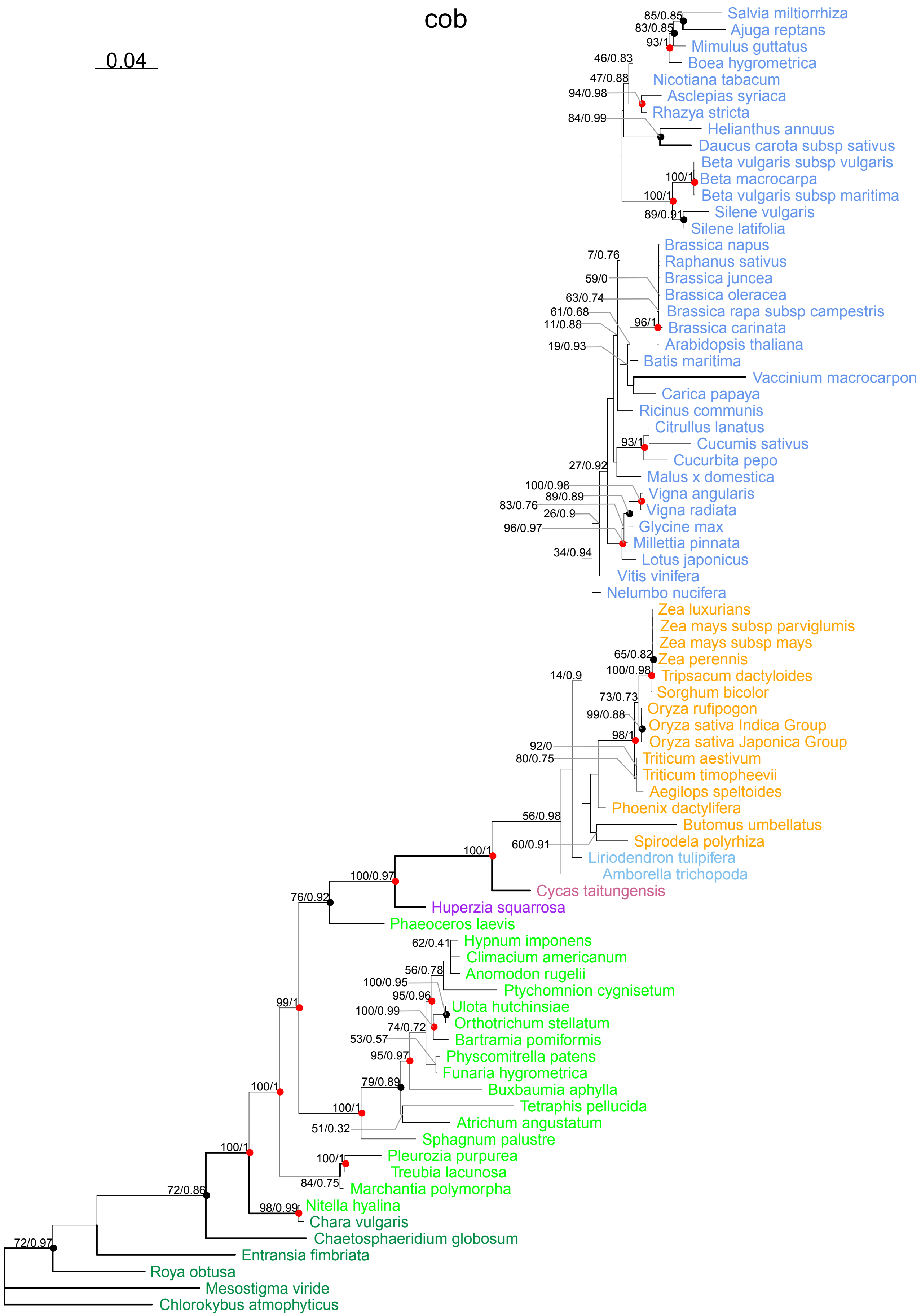

Figure S6 (continued)

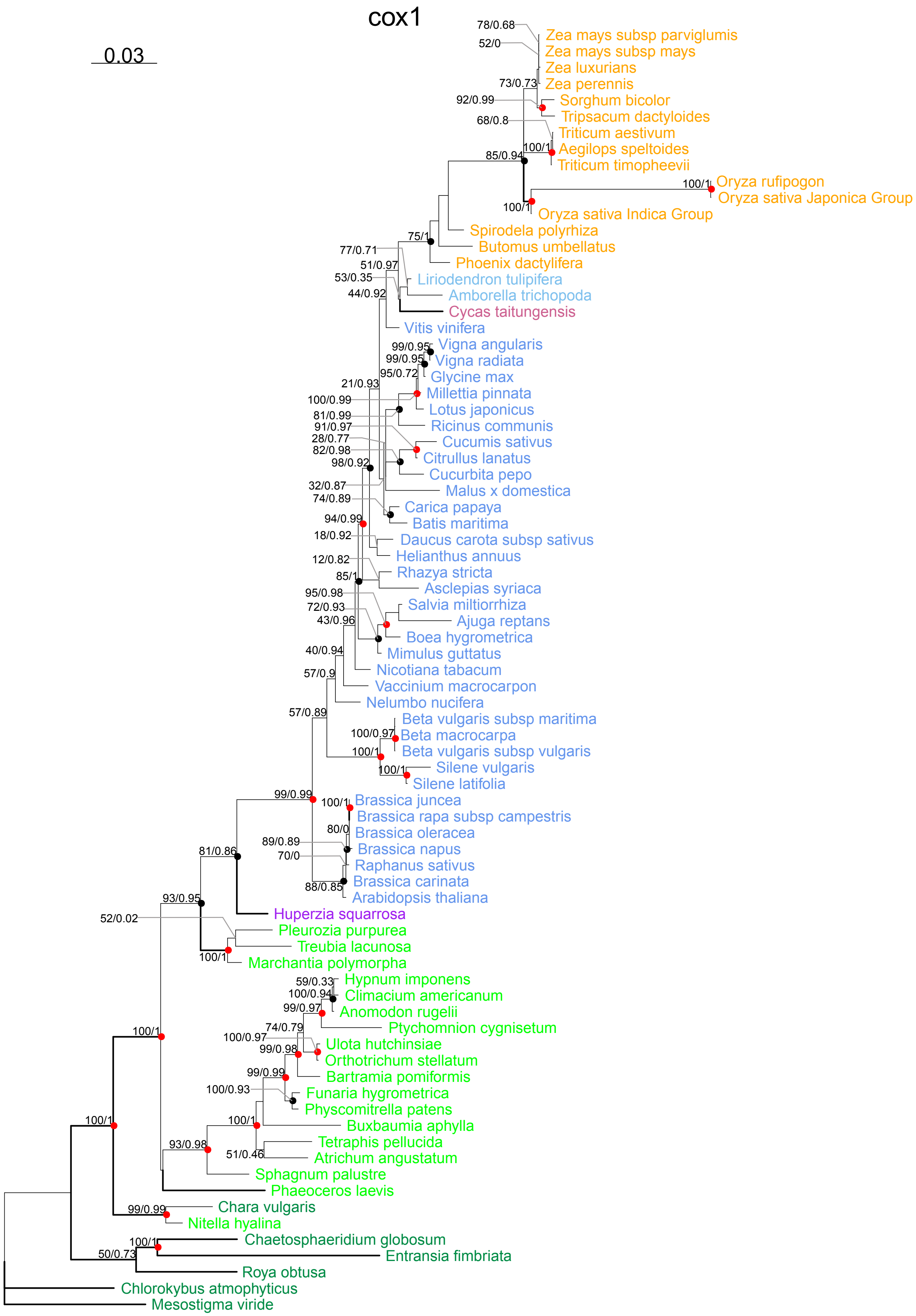

Figure S6 (continued)

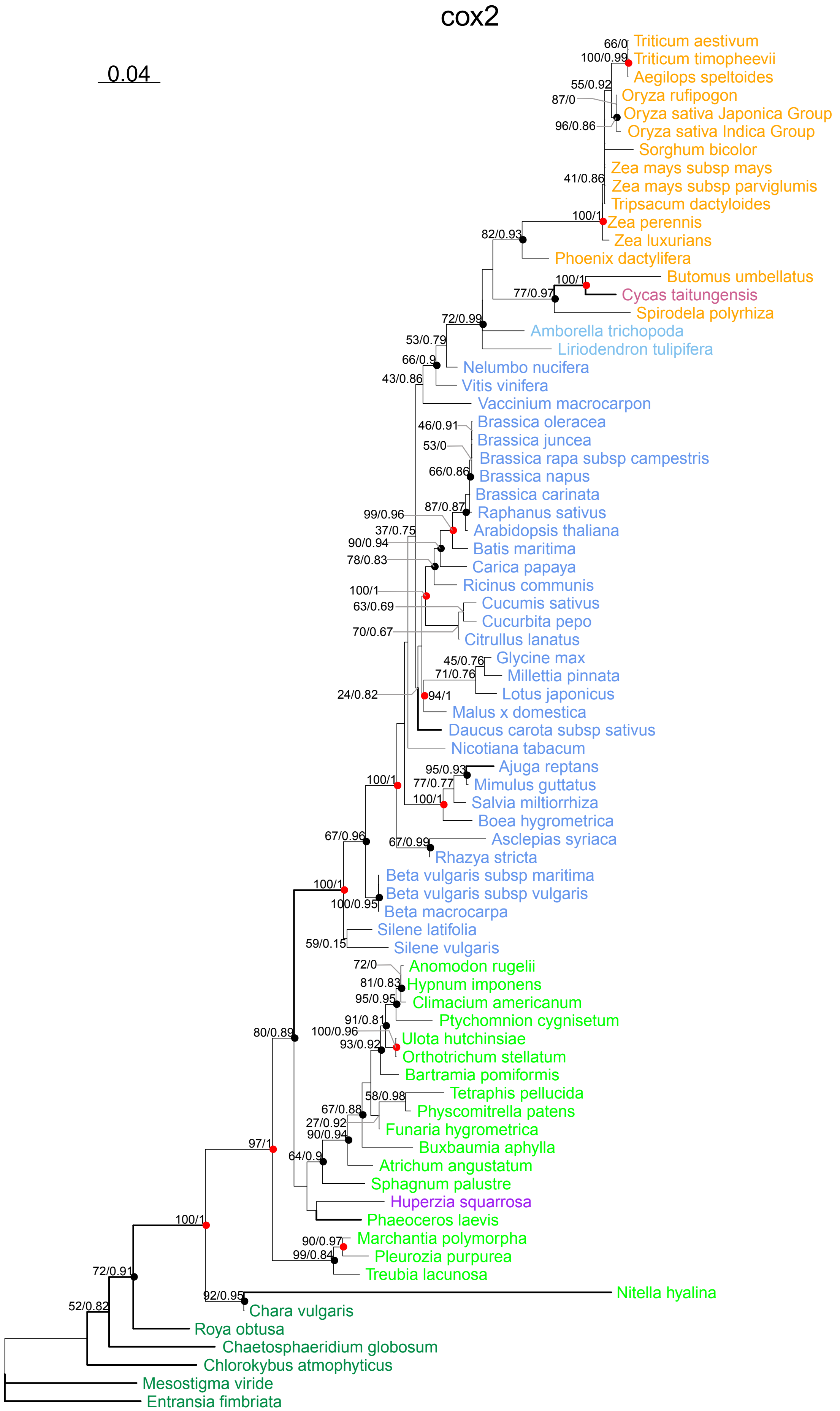

Figure S6 (continued)

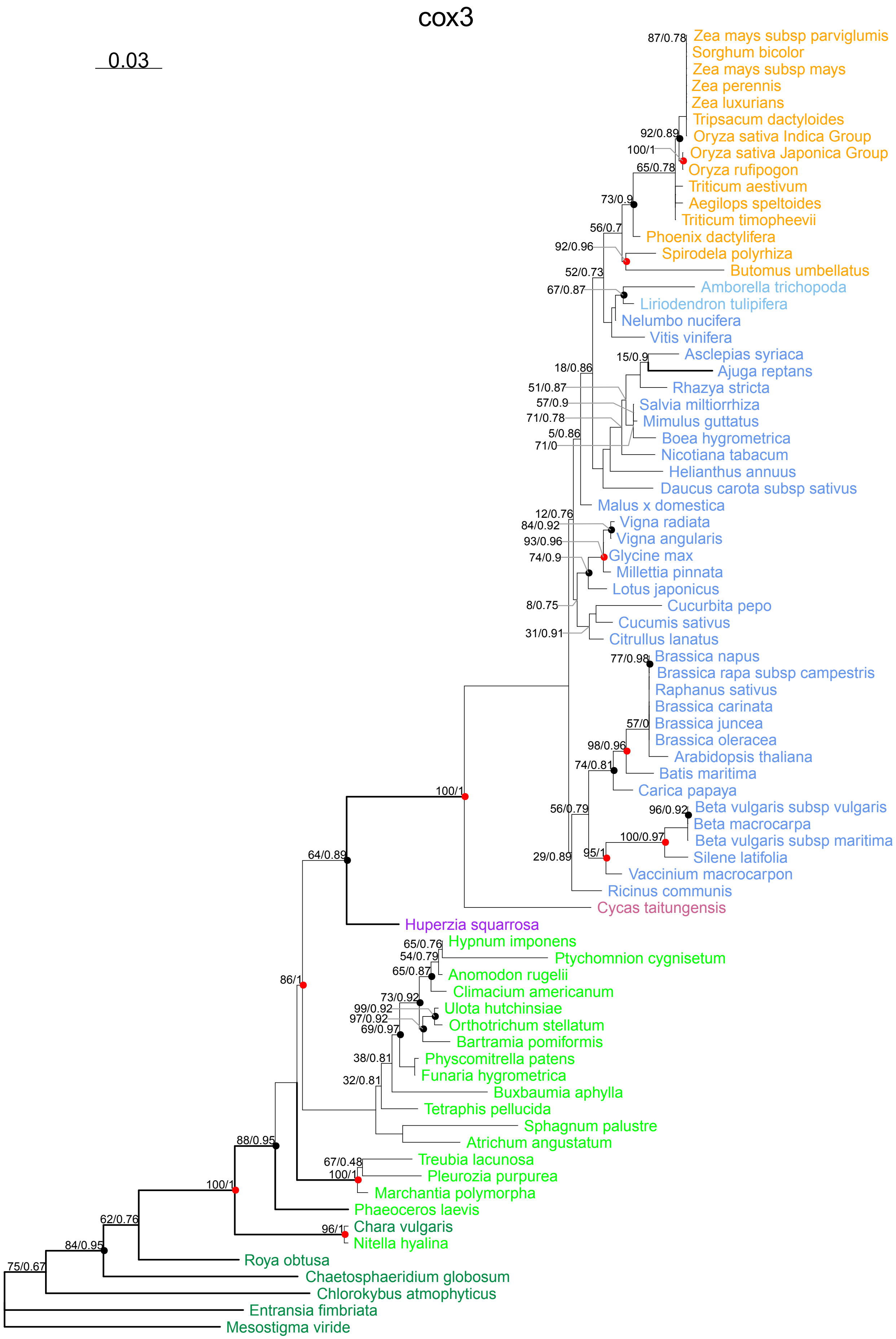

Figure S6 (continued)

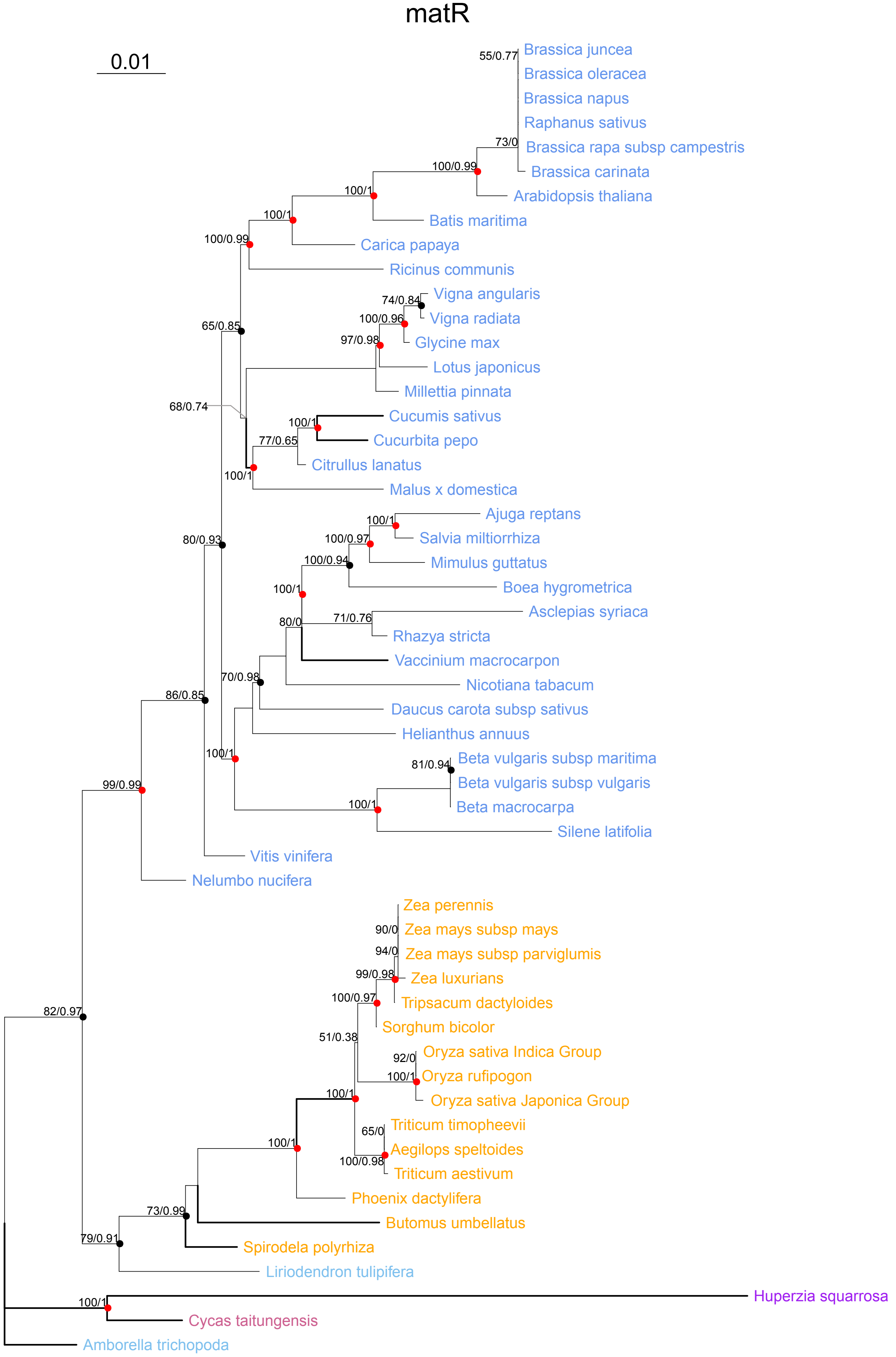

Figure S6 (continued)

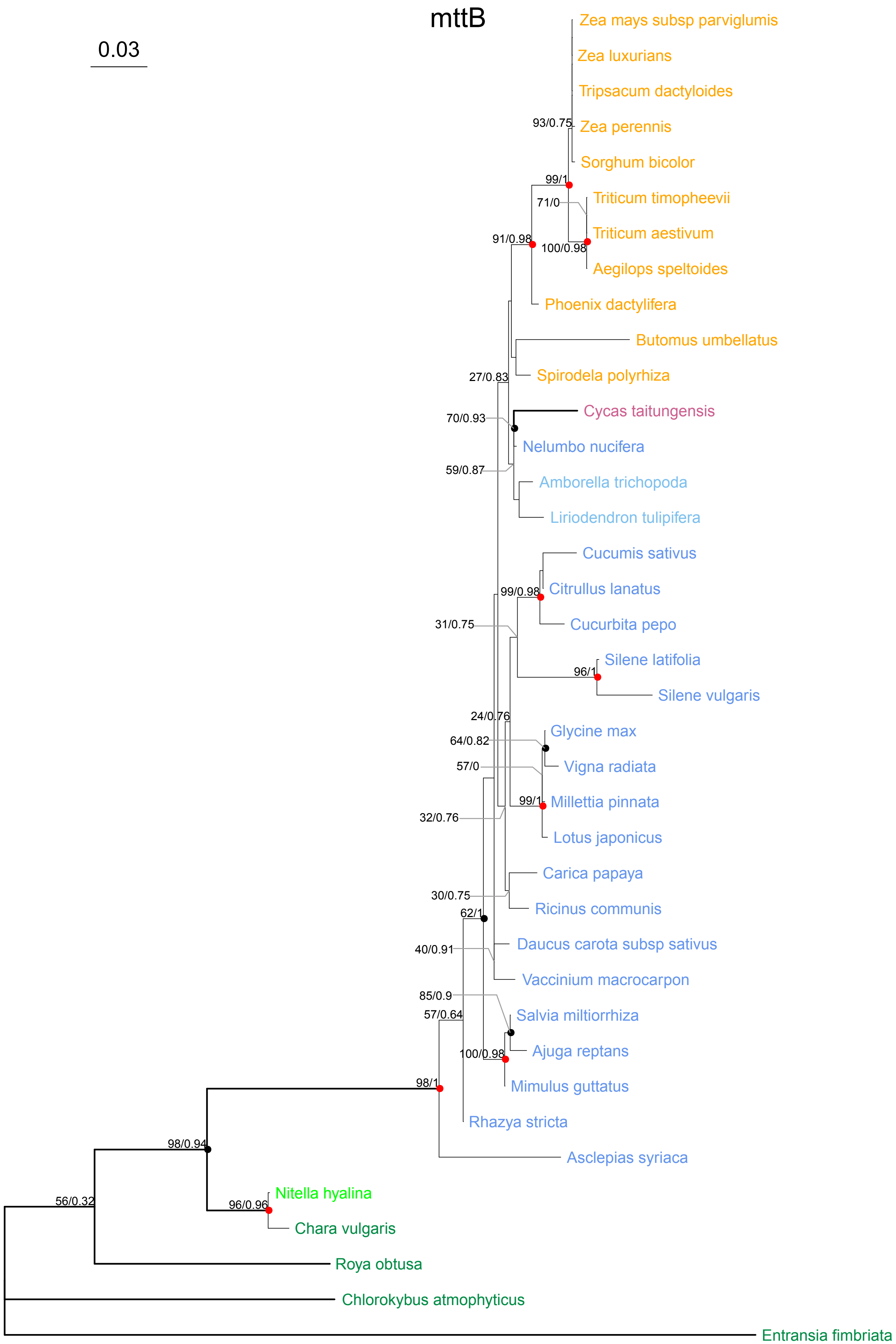

Figure S6 (continued)

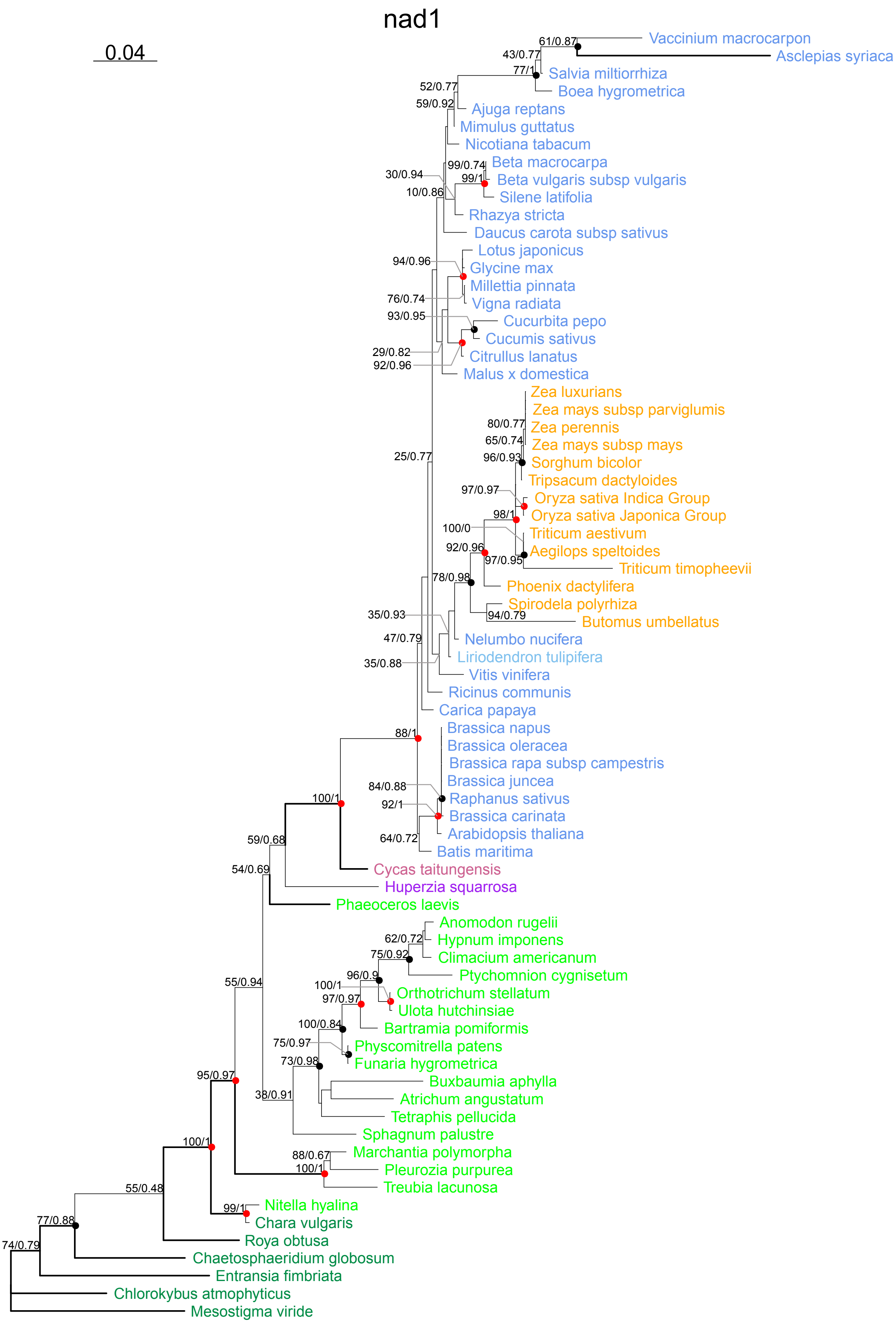

Figure S6 (continued)

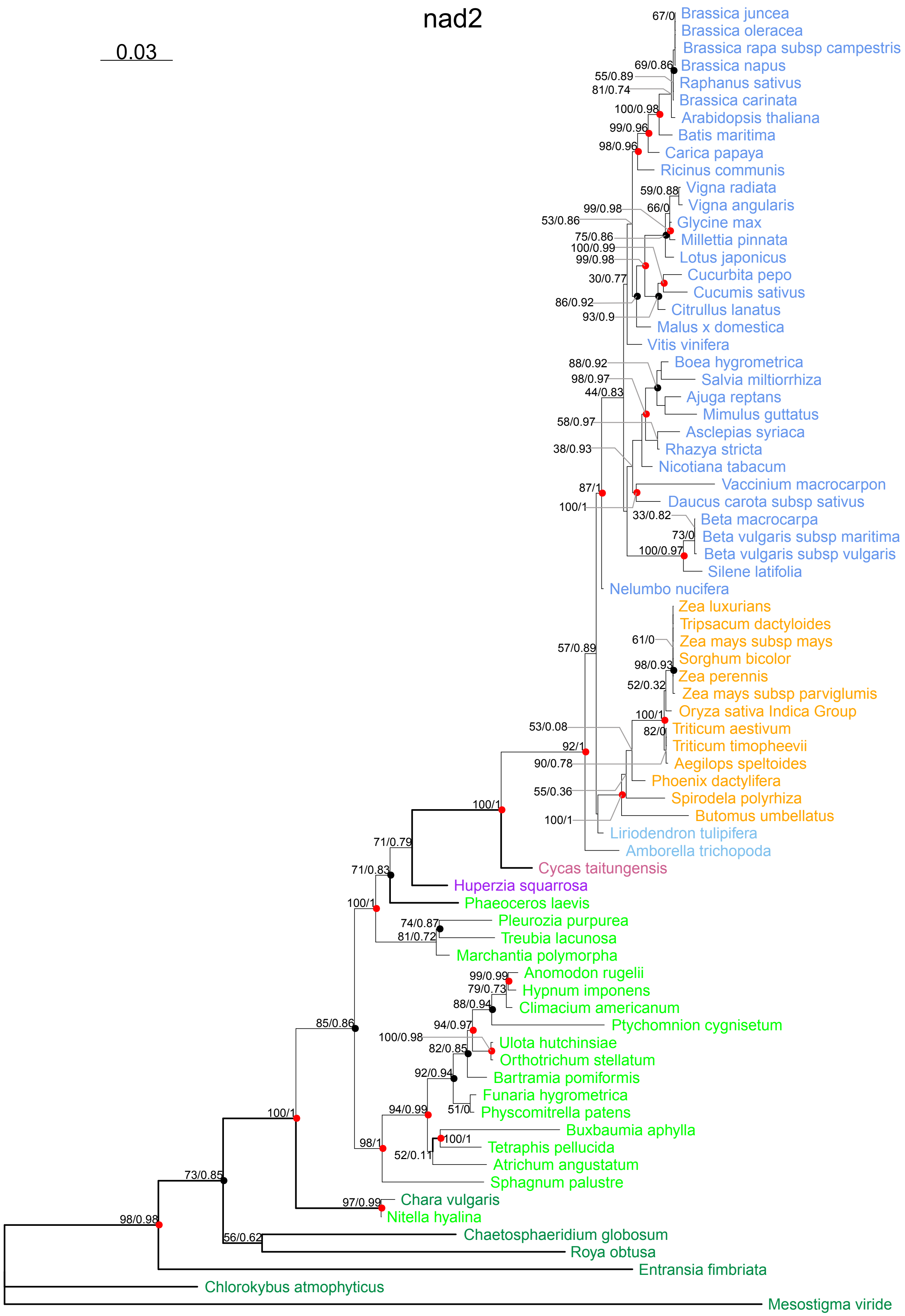

Figure S6 (continued)

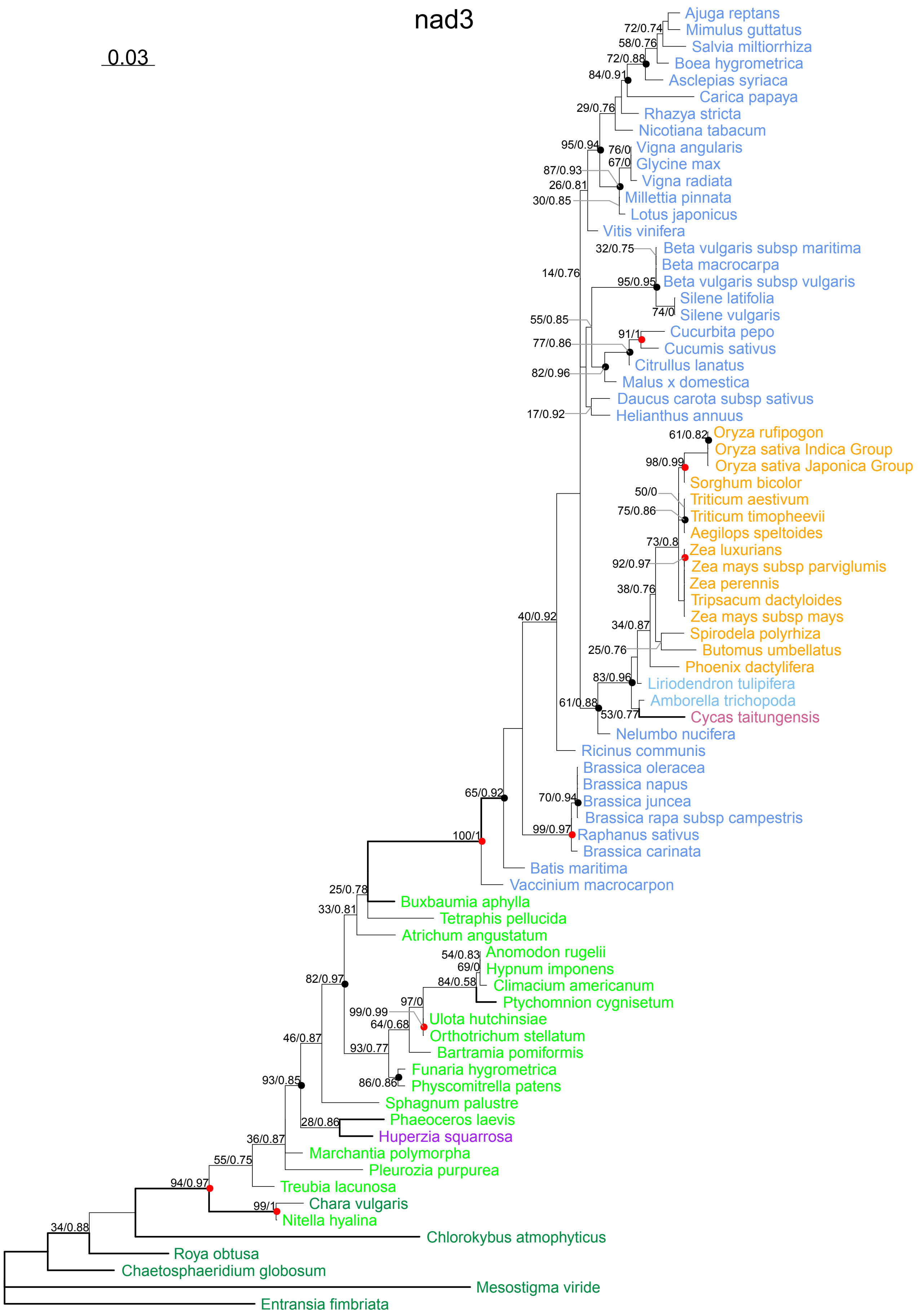

Figure S6 (continued)

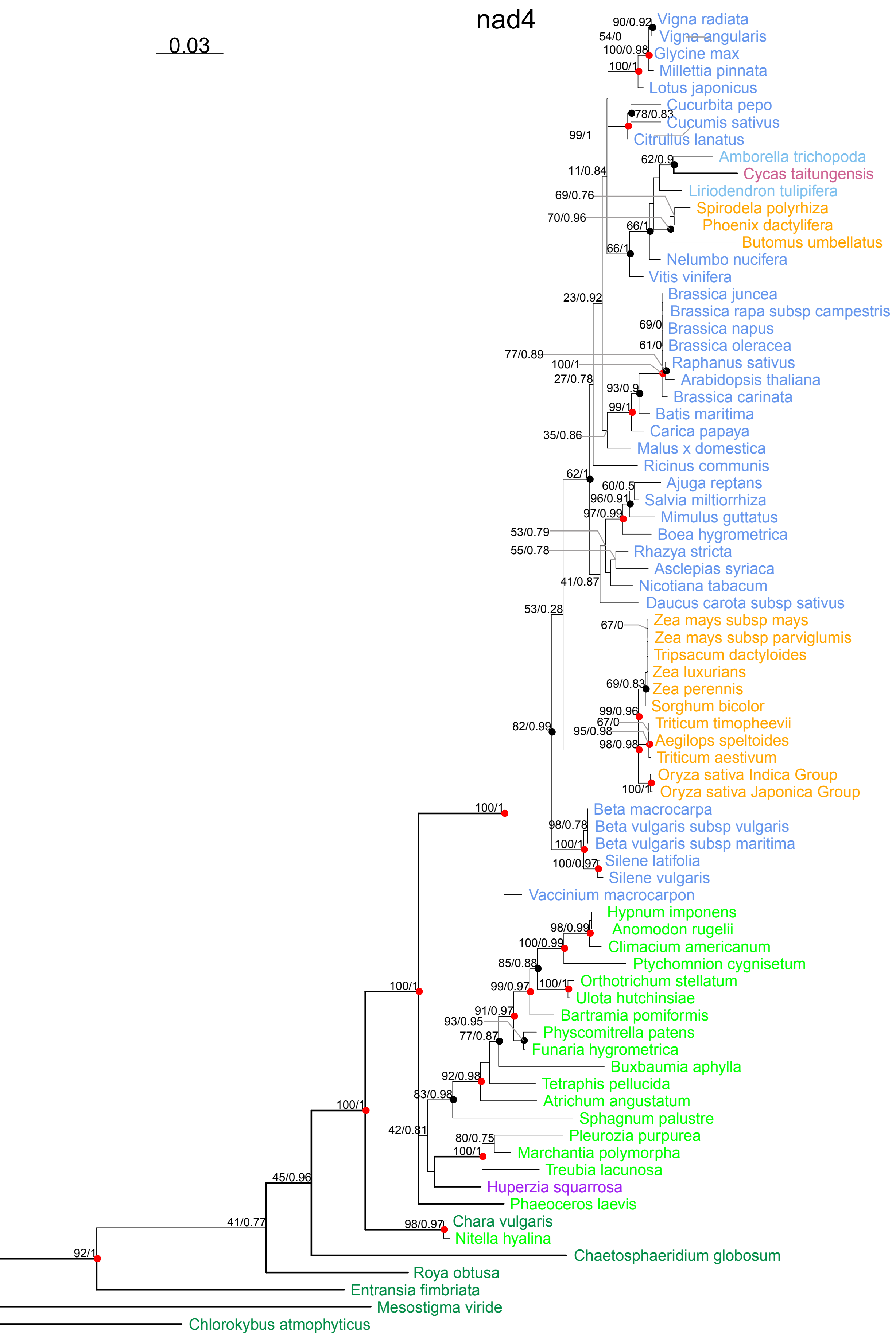

Figure S6 (continued)

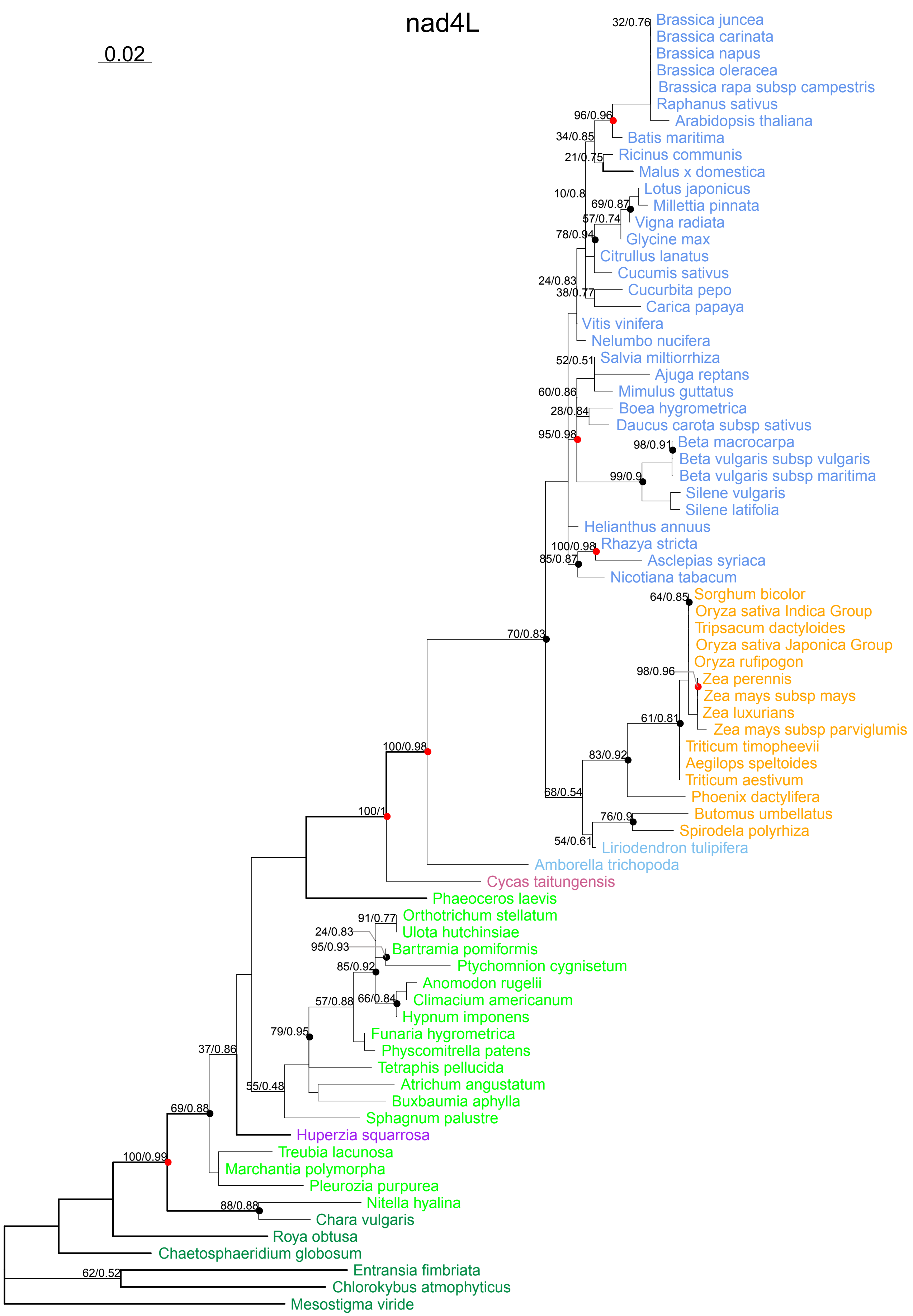

Figure S6 (continued)

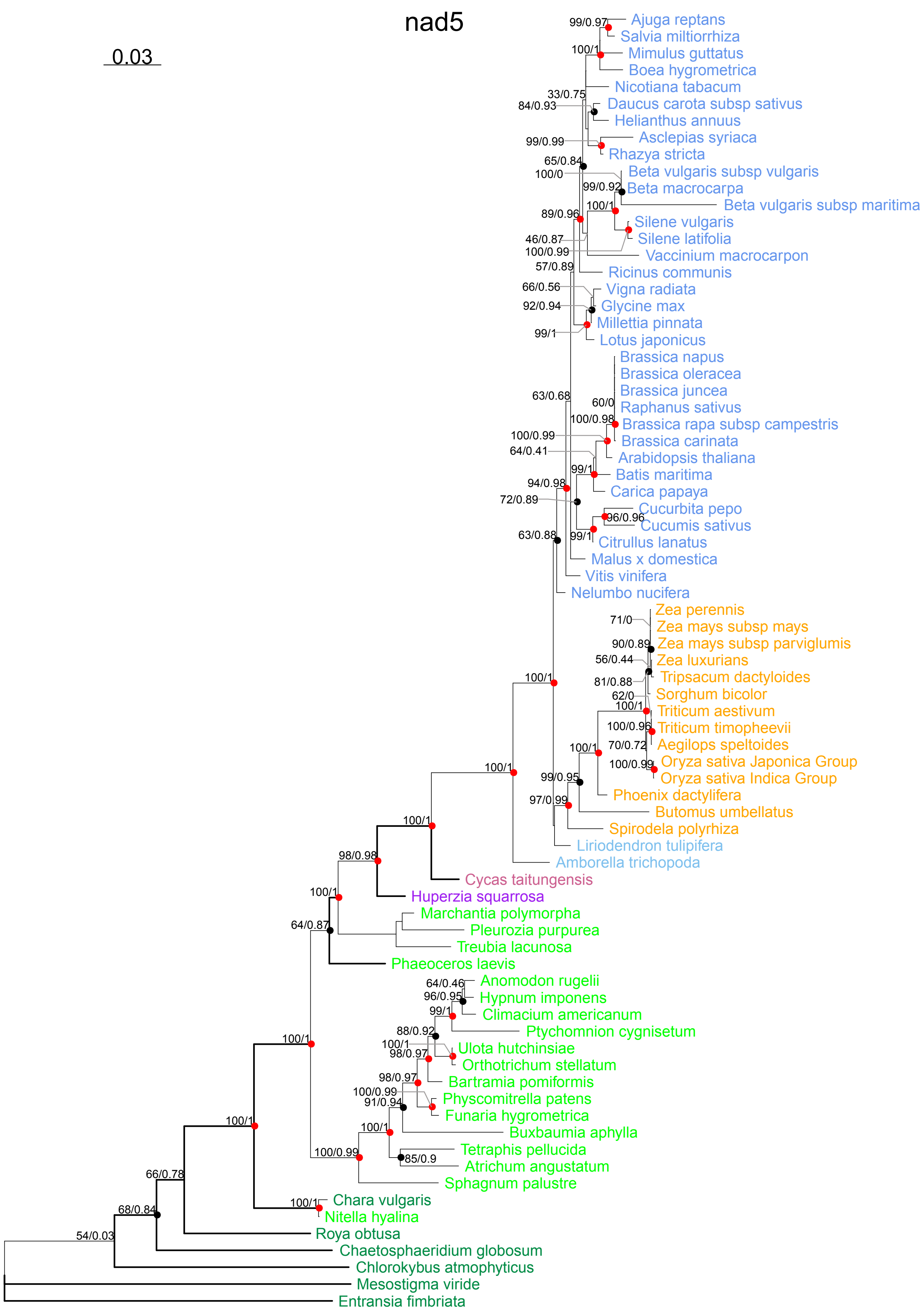

Figure S6 (continued)

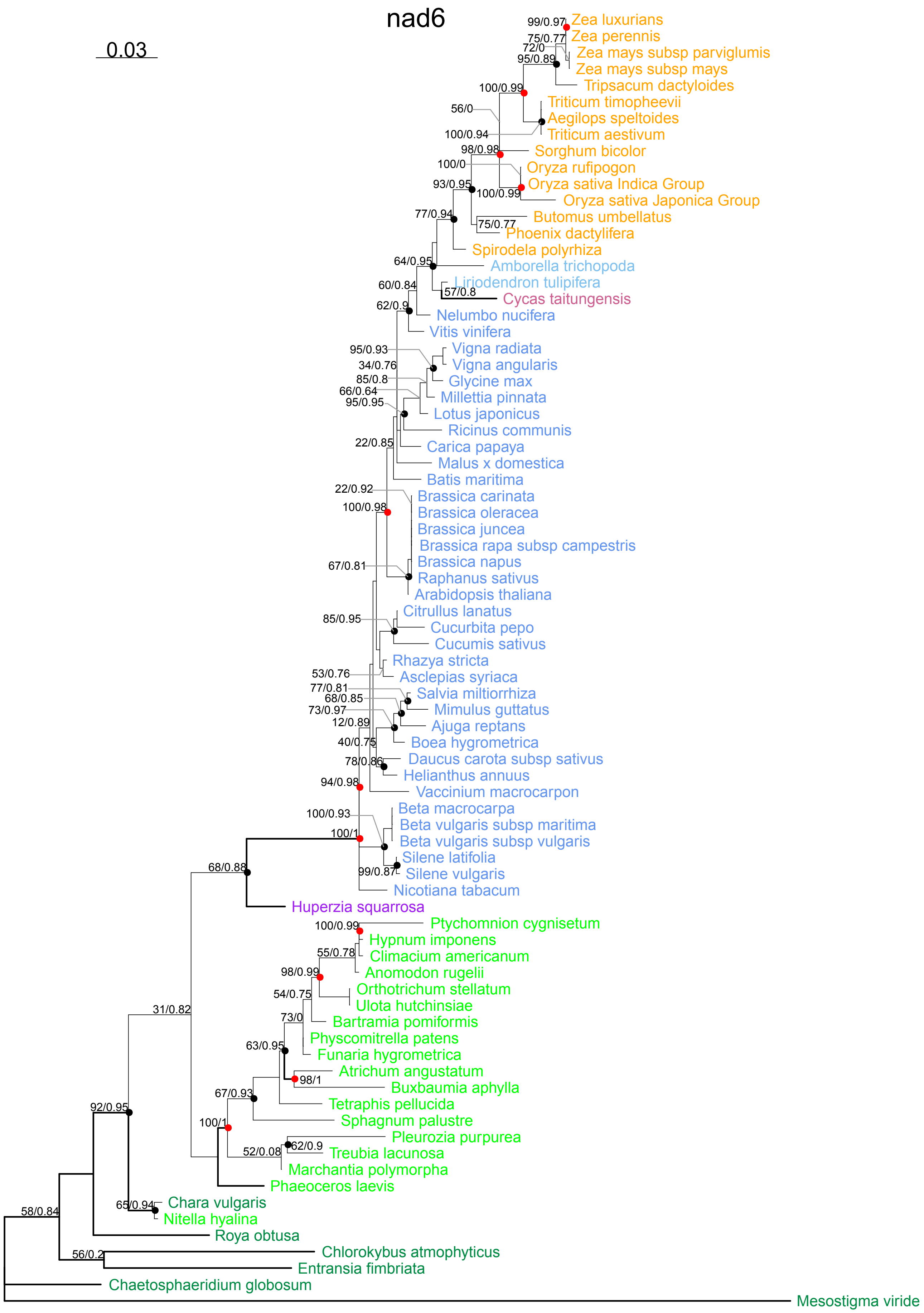

Figure S6 (continued)

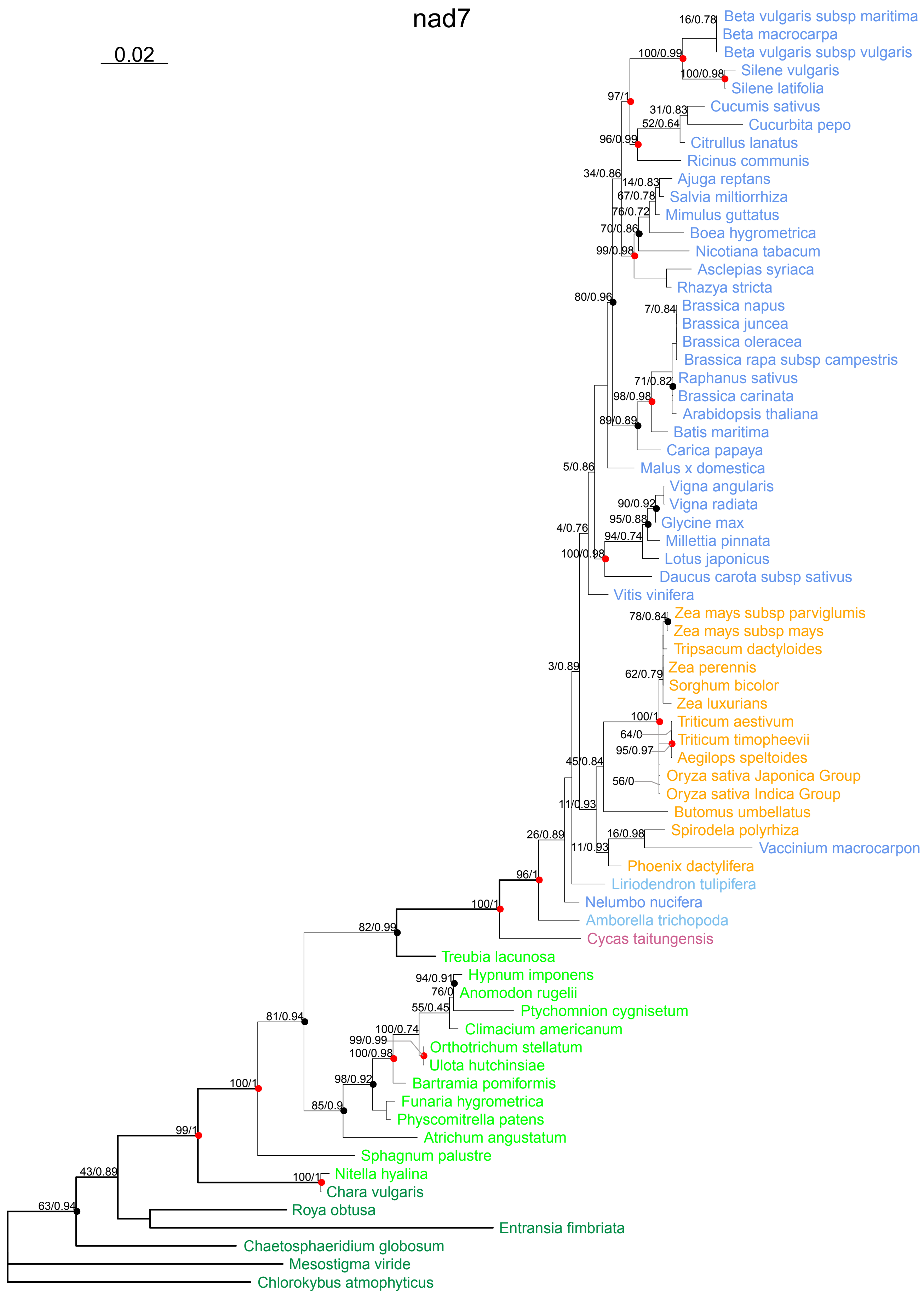

Figure S6 (continued)

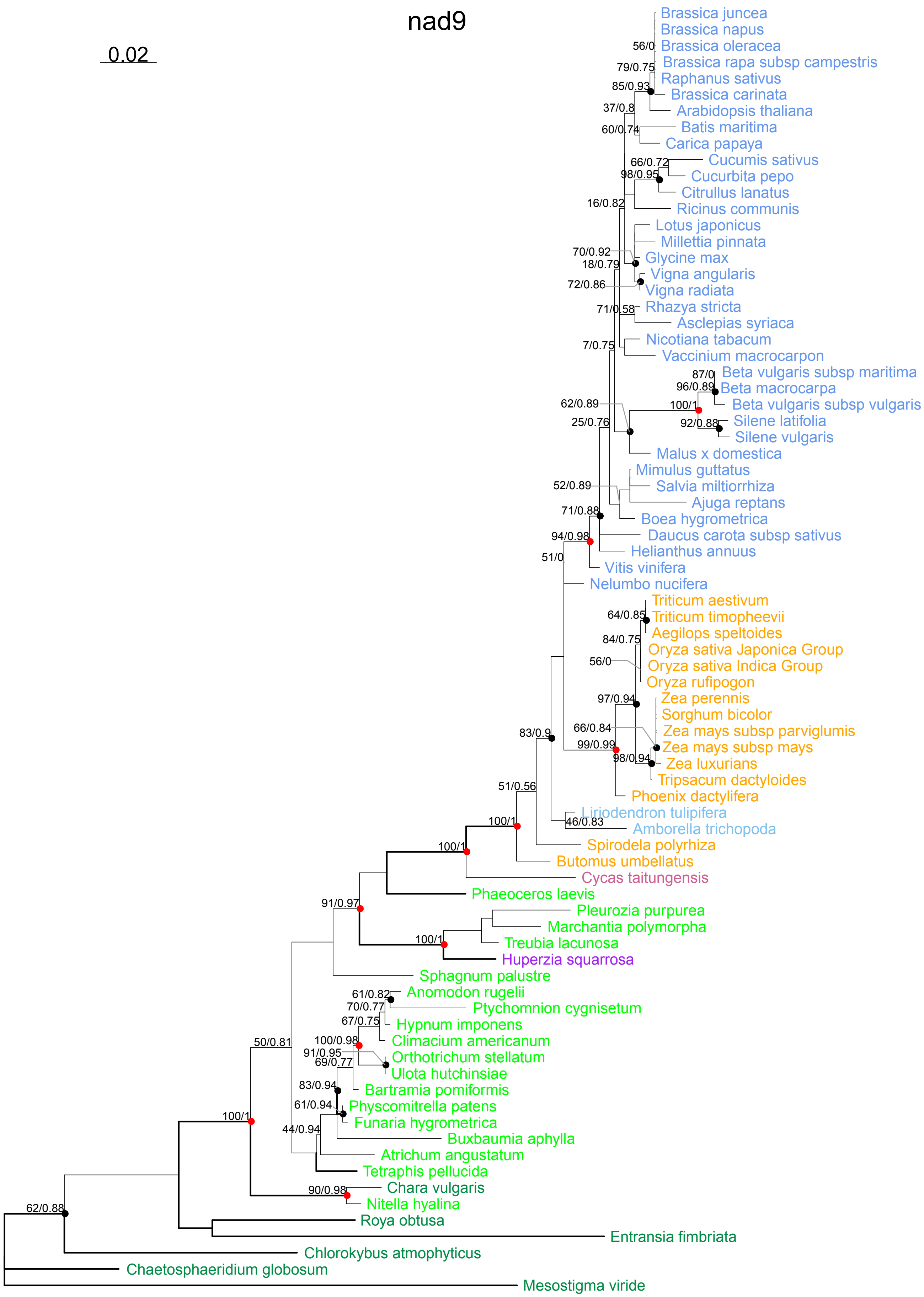

Figure S6 (continued)

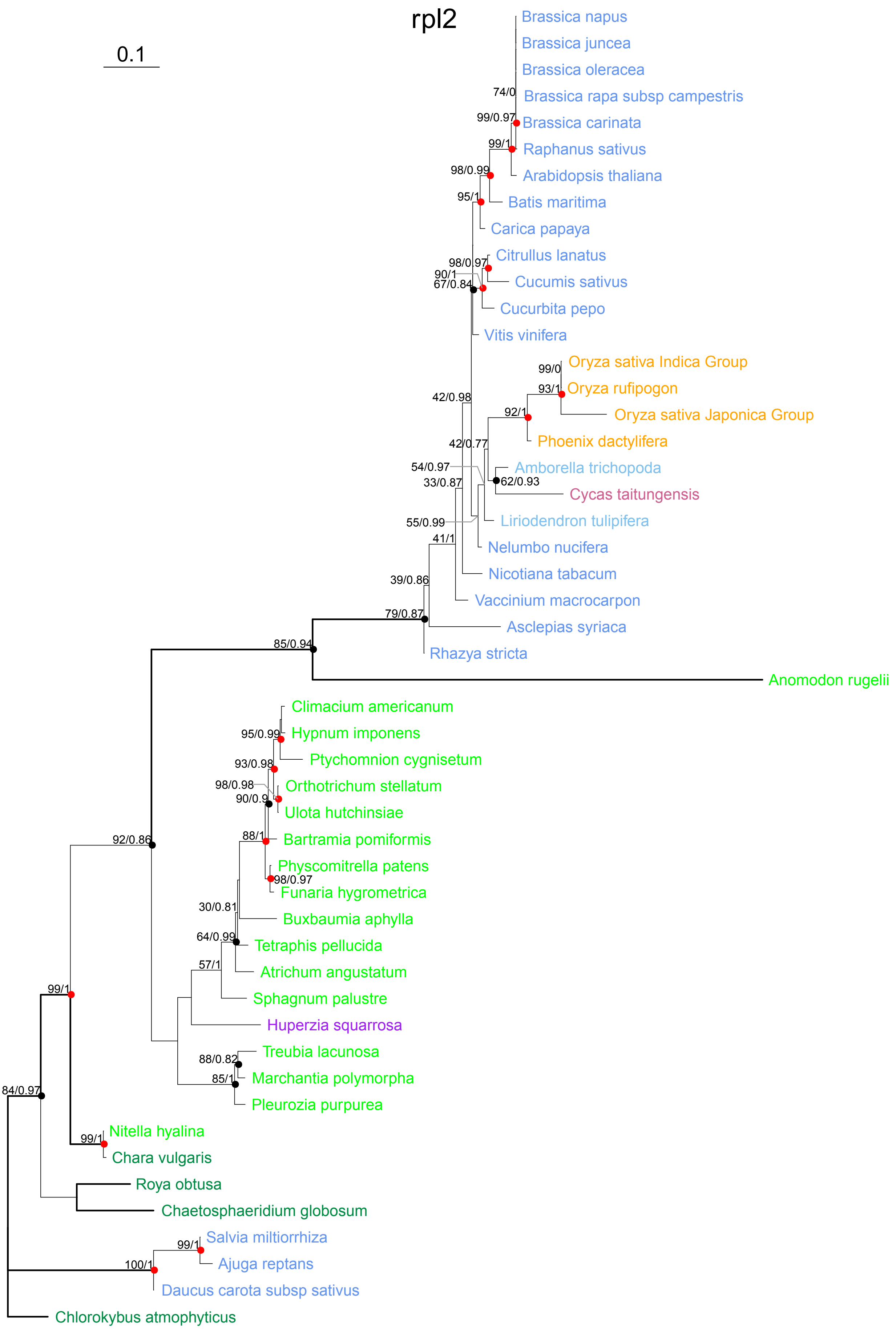

Figure S6 (continued)

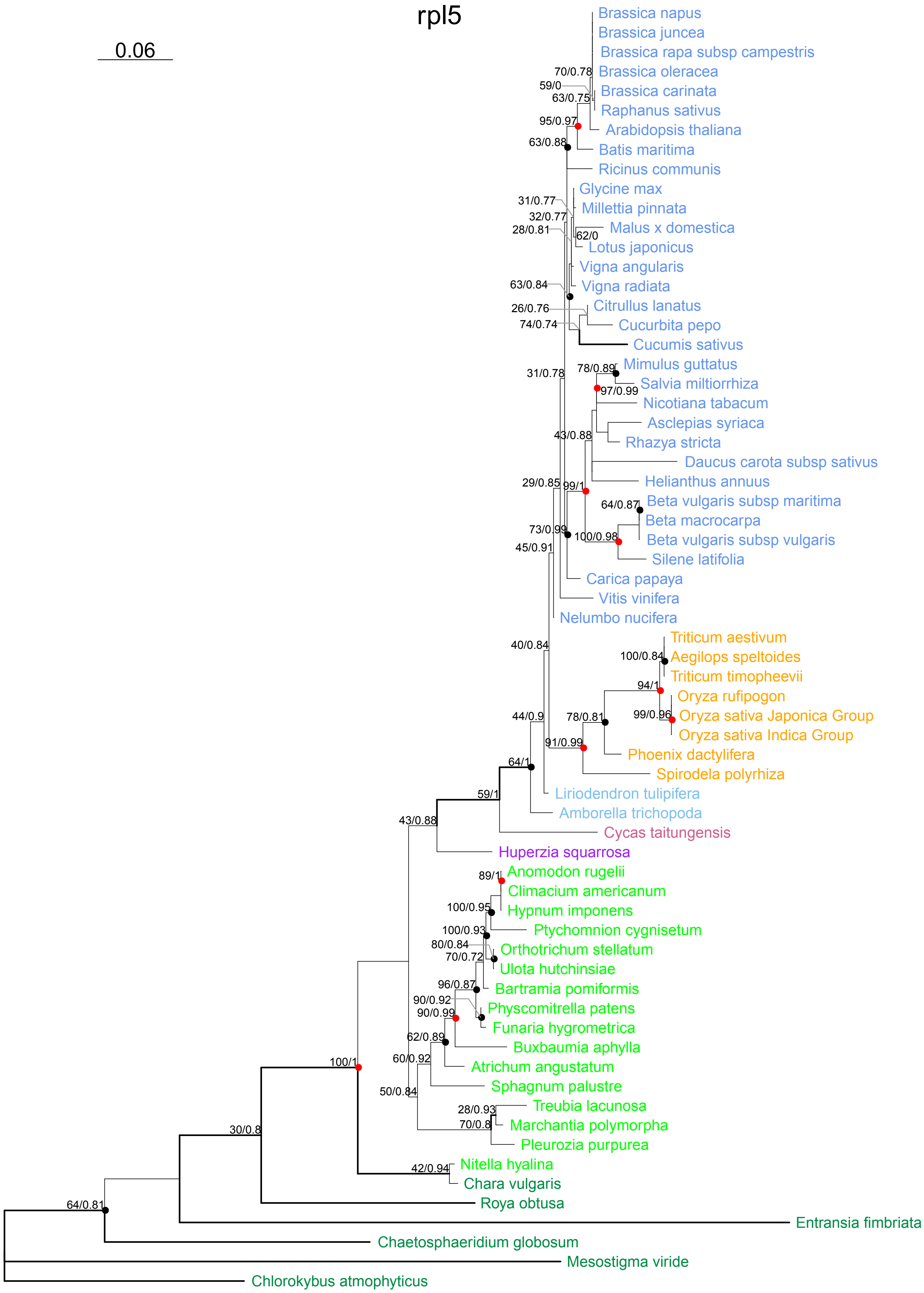

Figure S6 (continued)

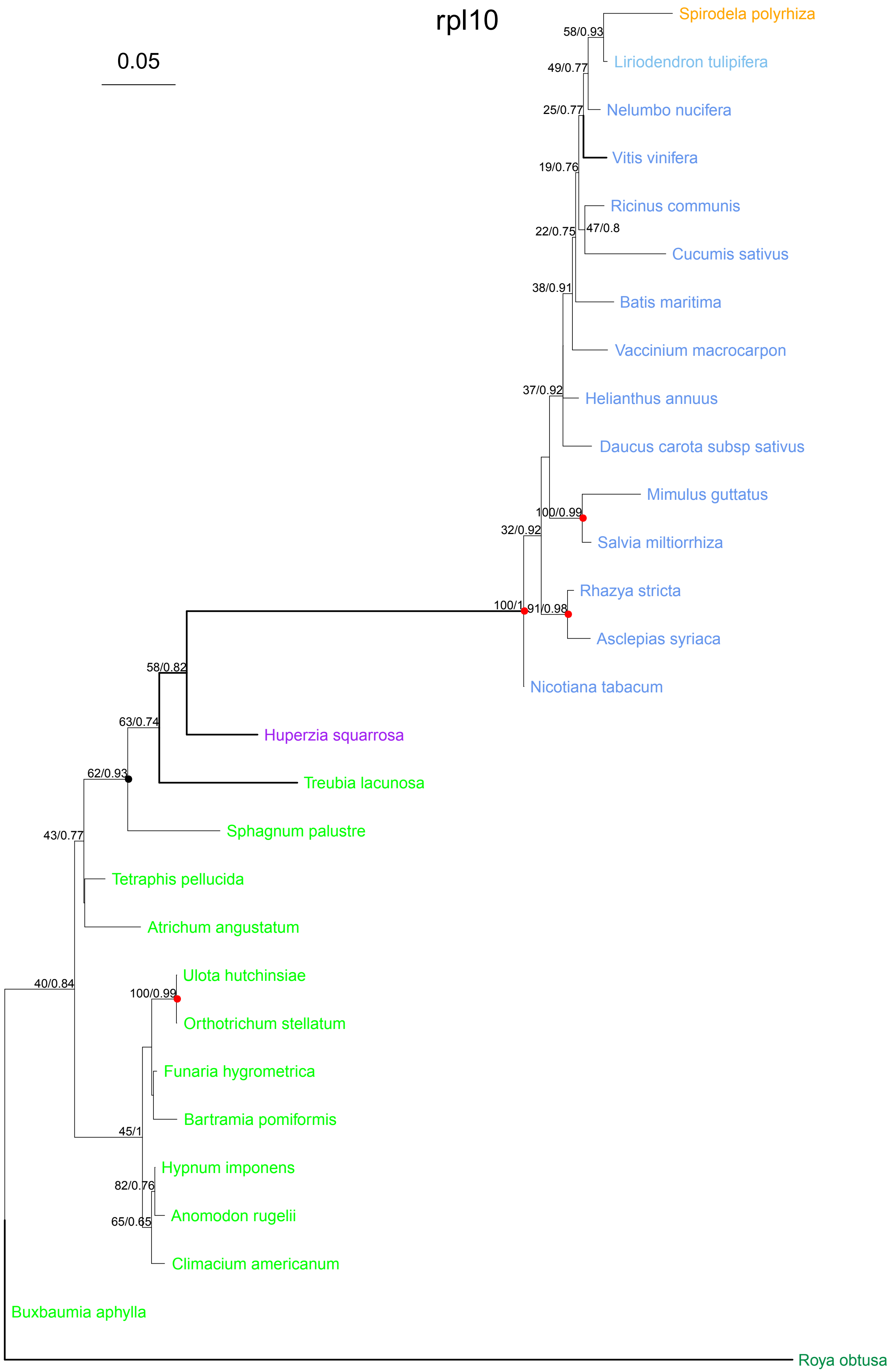

Figure S6 (continued)

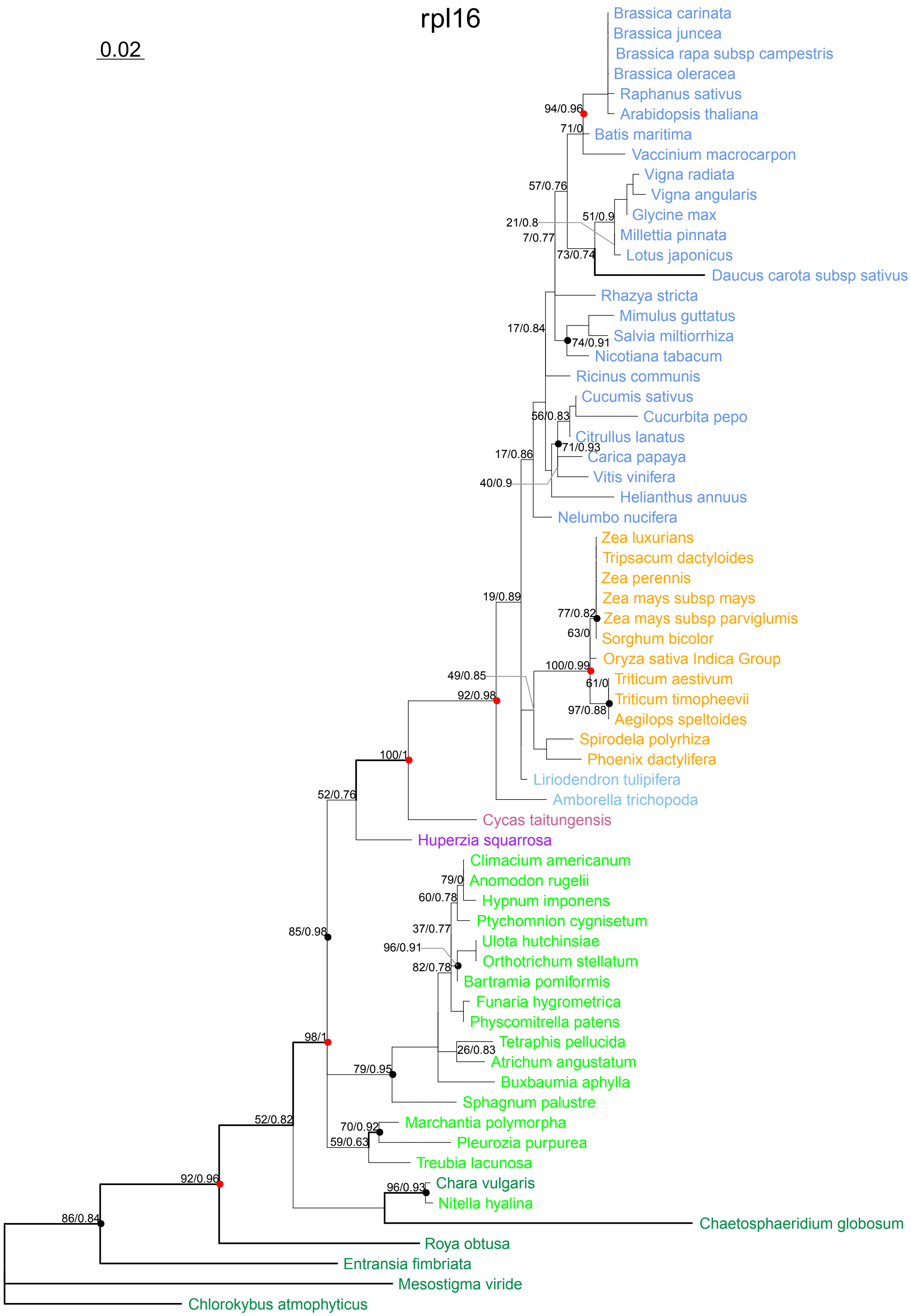

Figure S6 (continued)

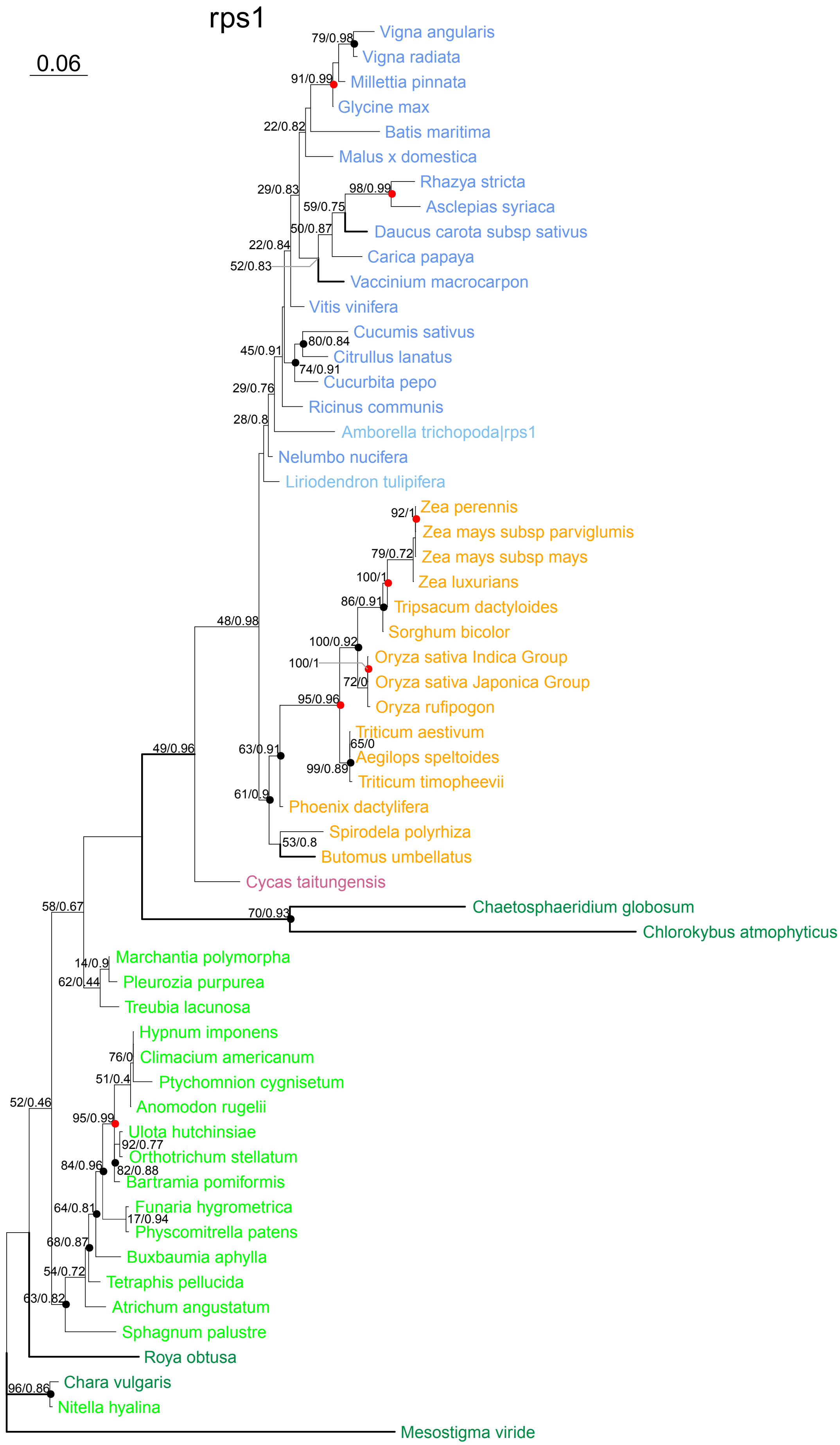

Figure S6 (continued)

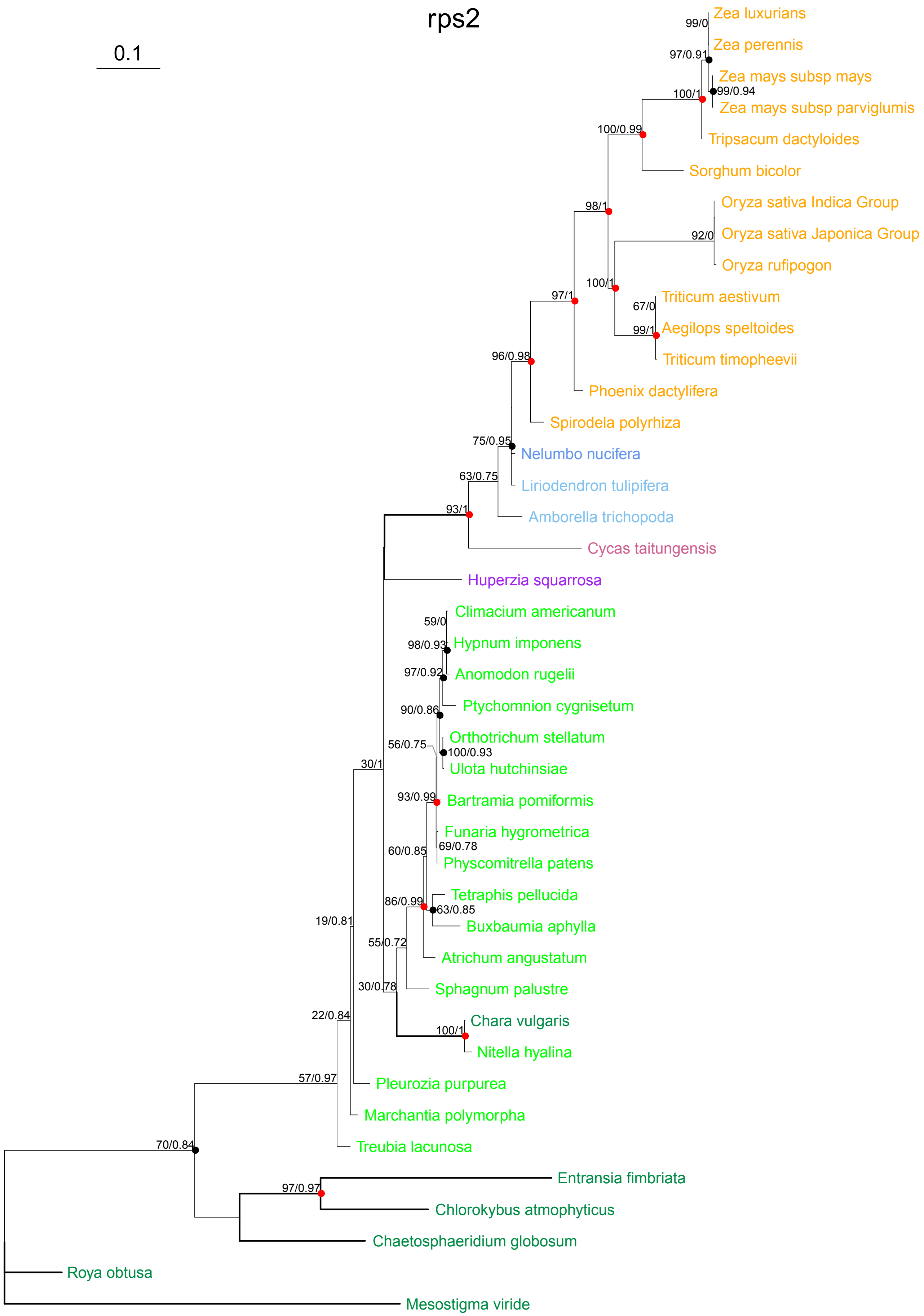

Figure S6 (continued)

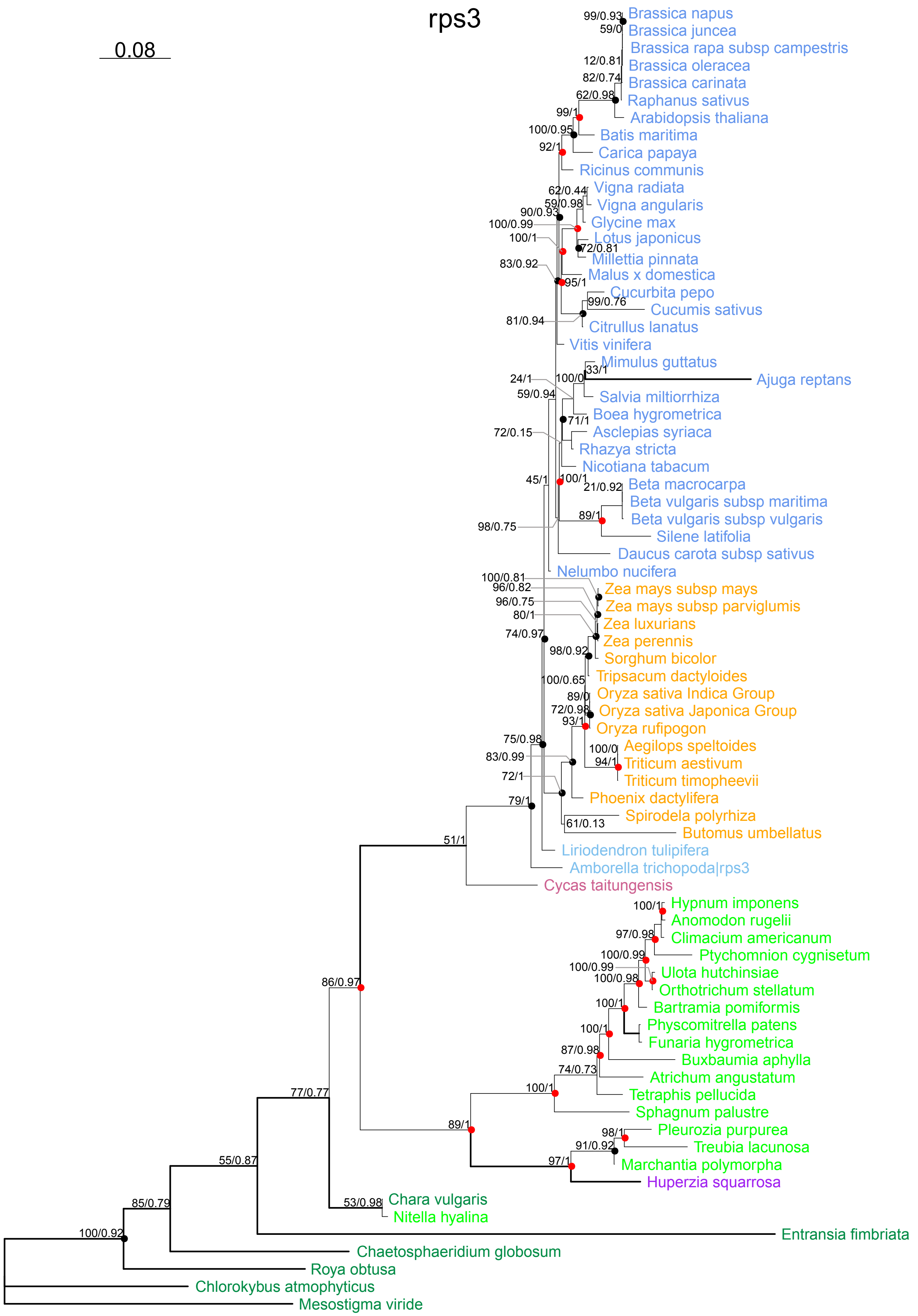

Figure S6 (continued)

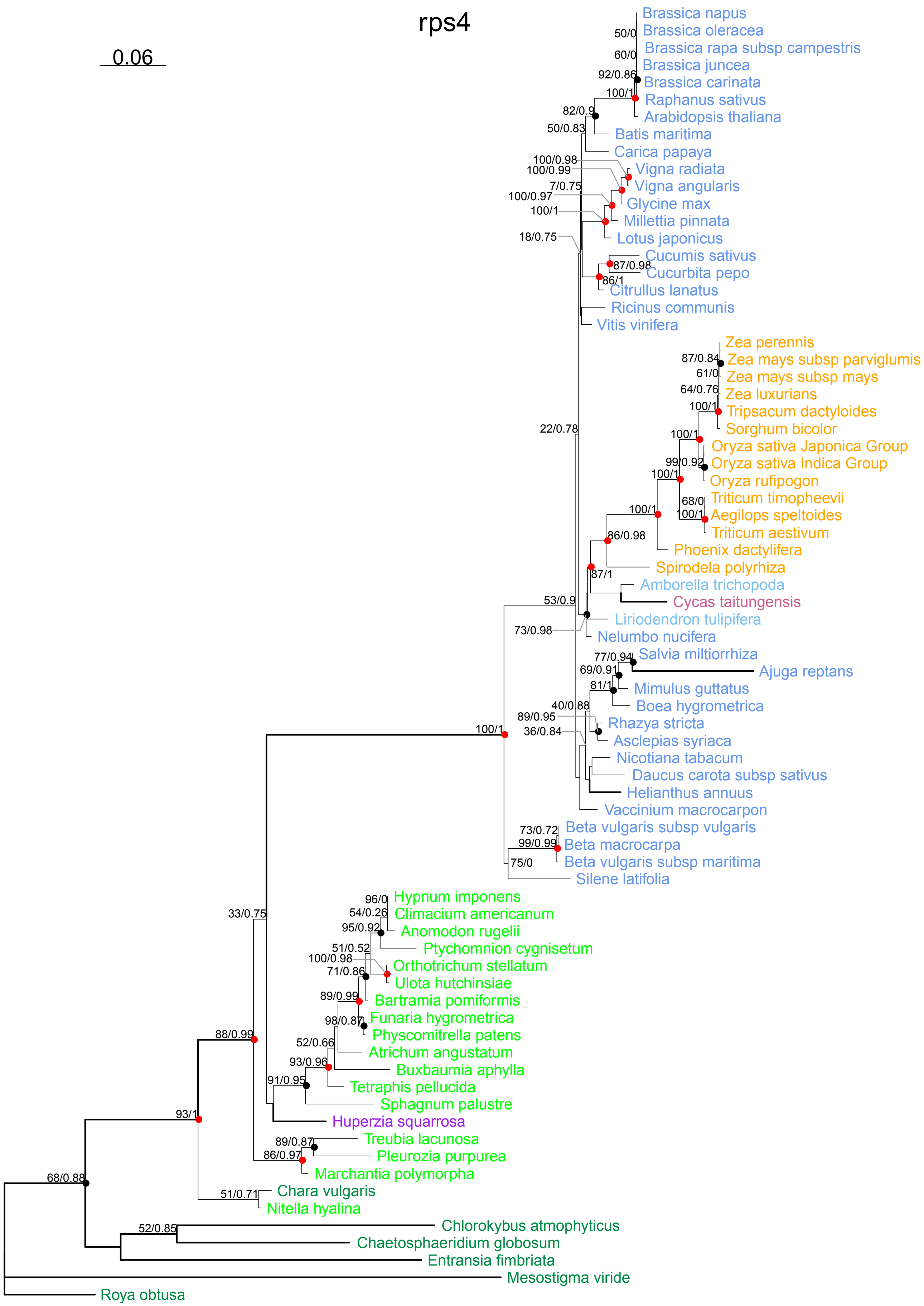

Figure S6 (continued)

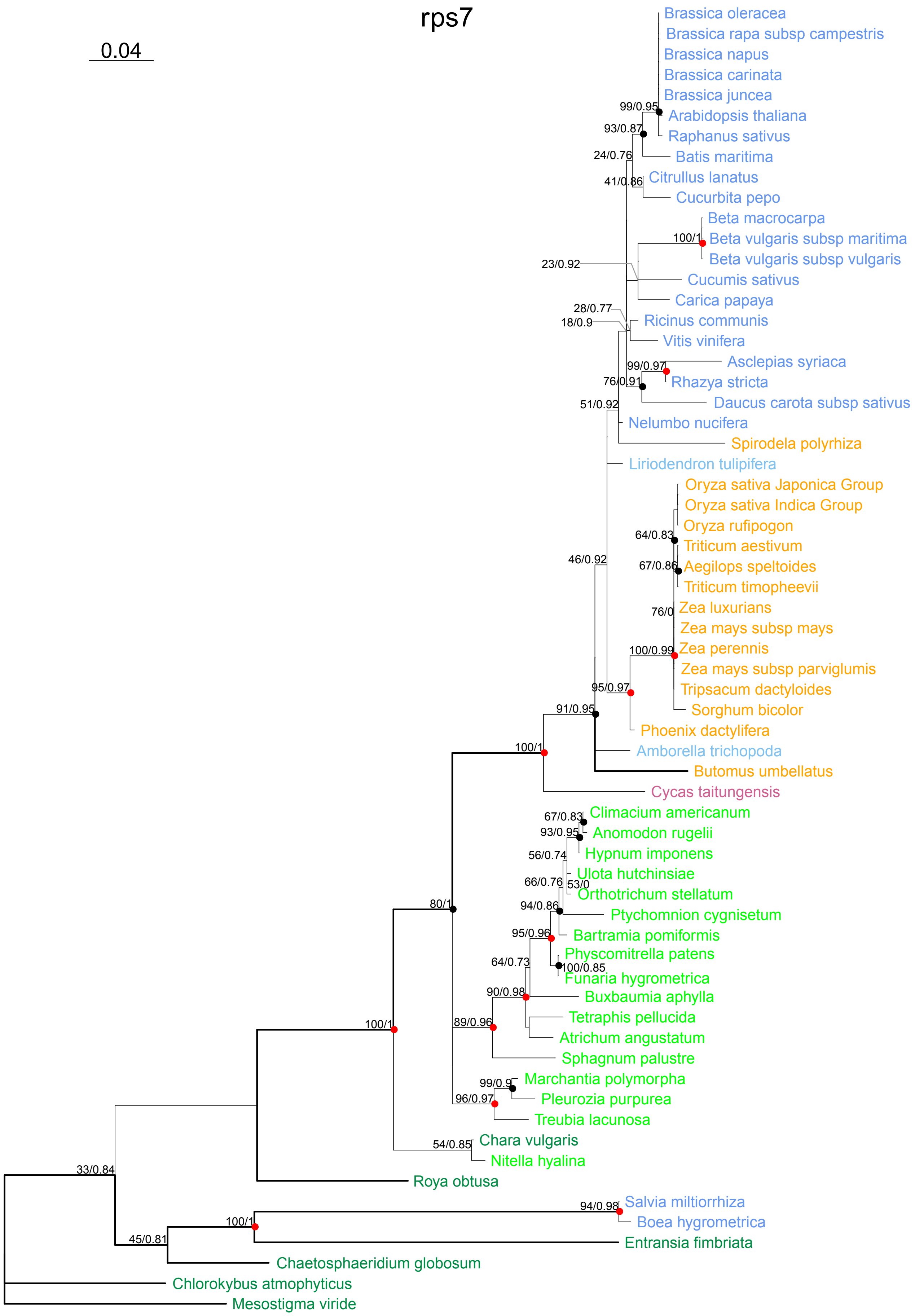

Figure S6 (continued)

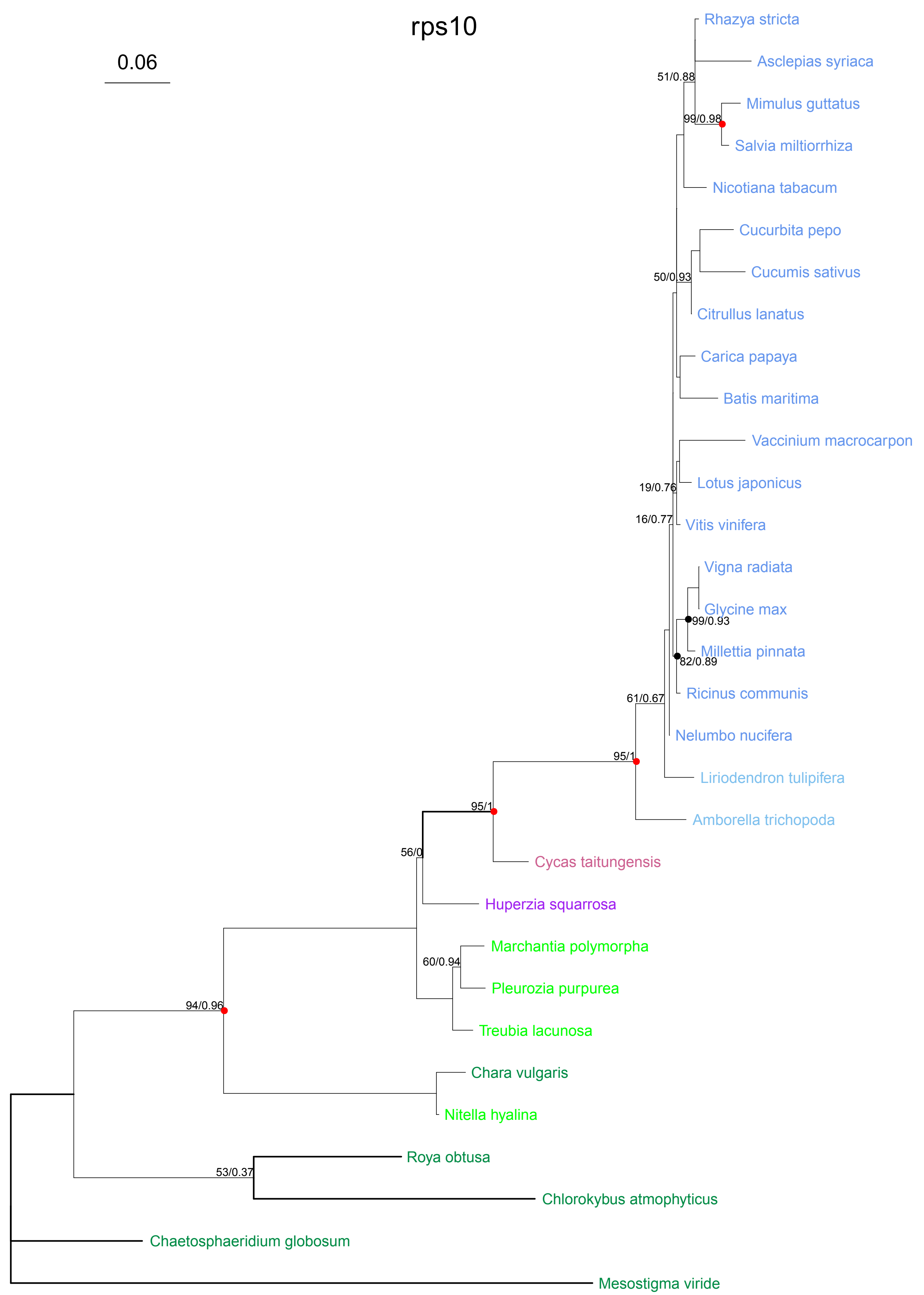

Figure S6 (continued)

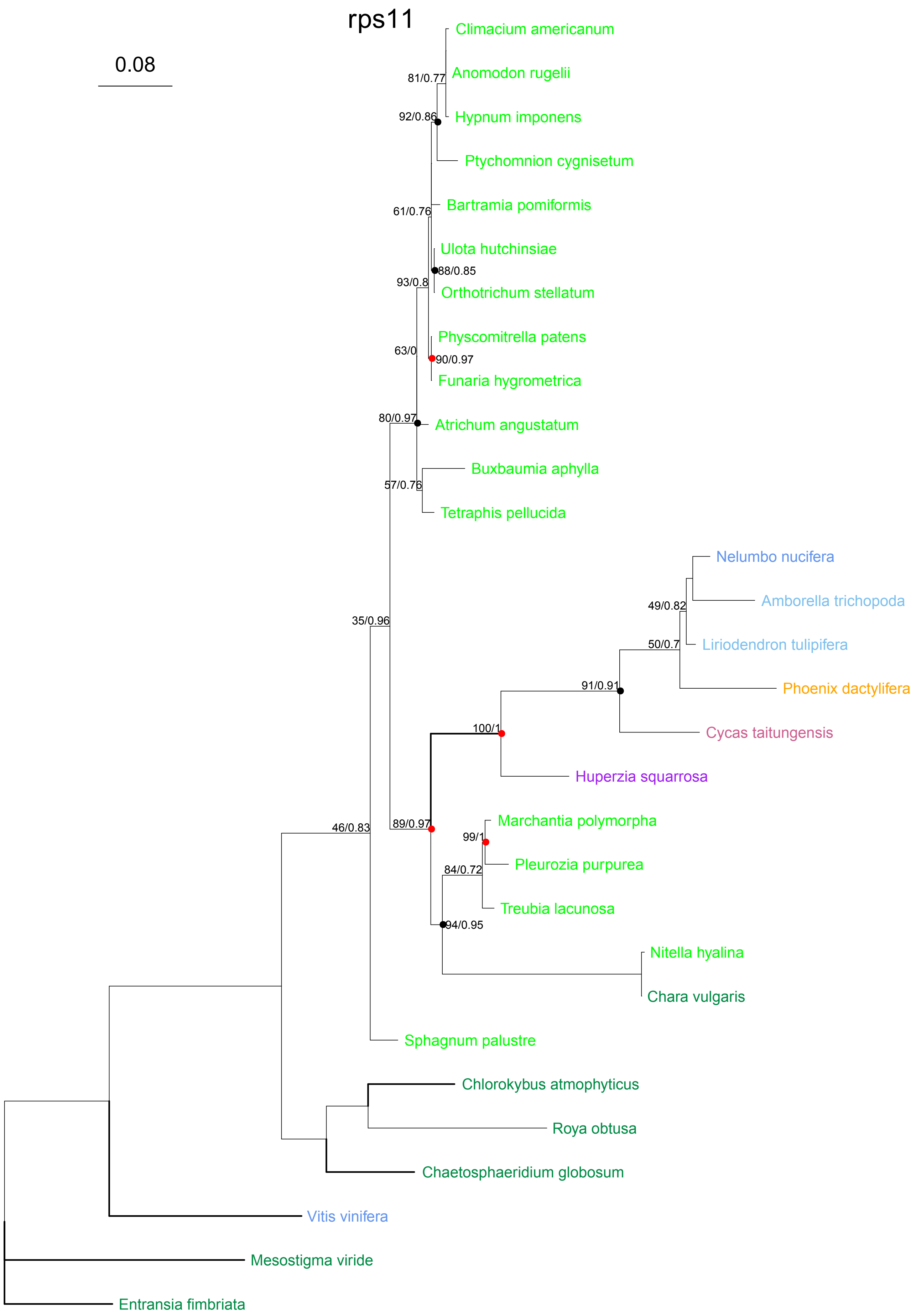

Figure S6 (continued)

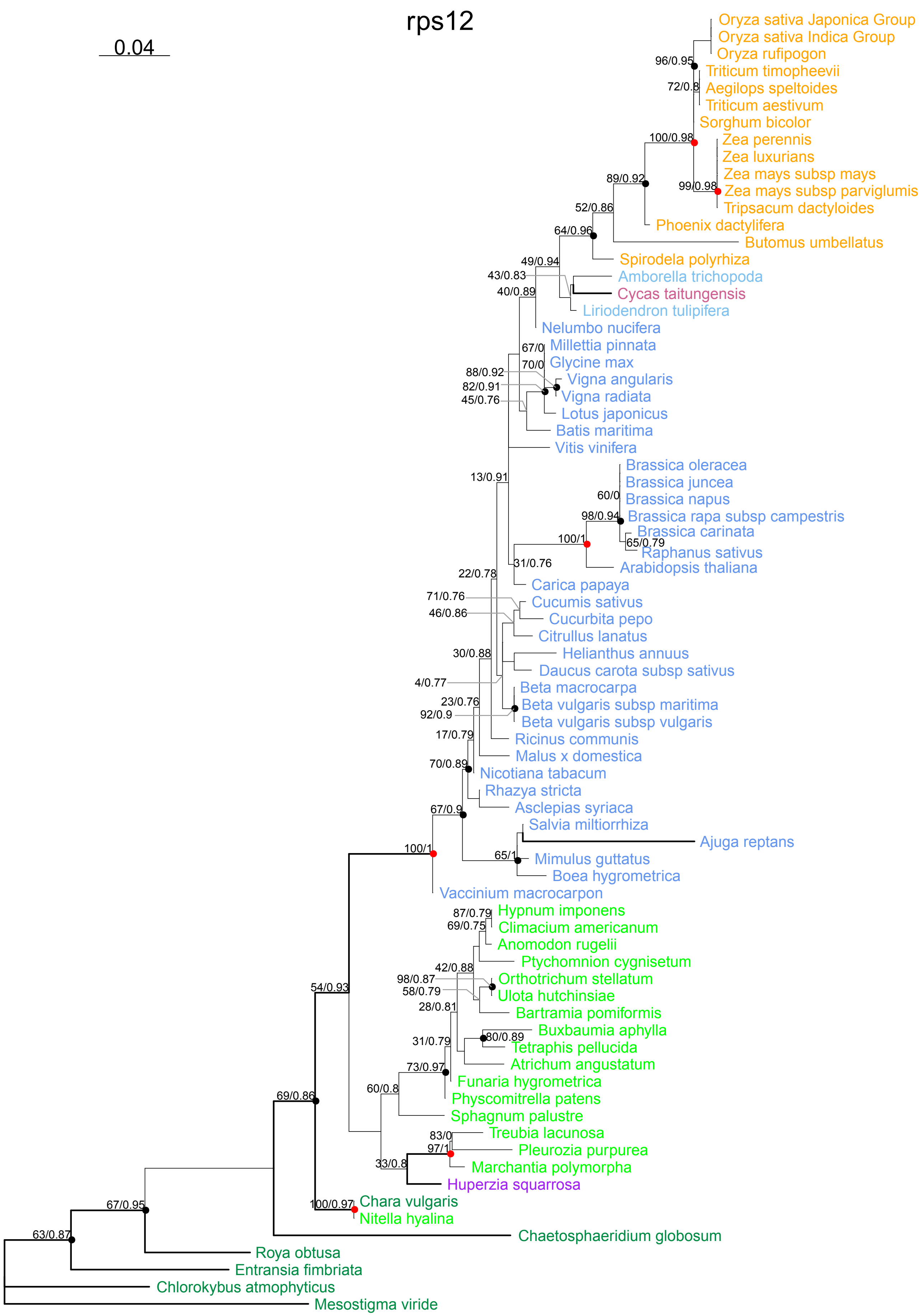

Figure S6 (continued)

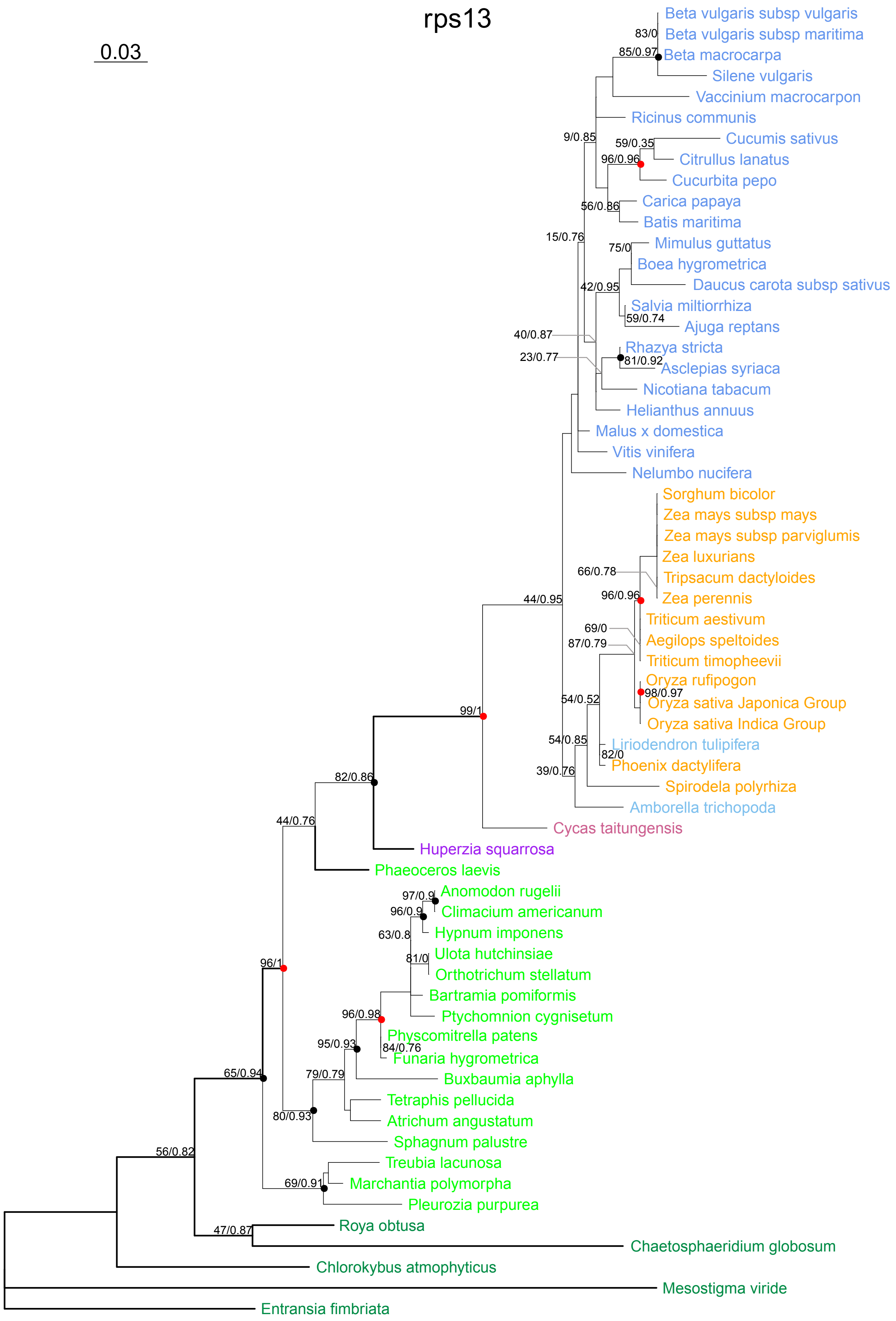

Figure S6 (continued)

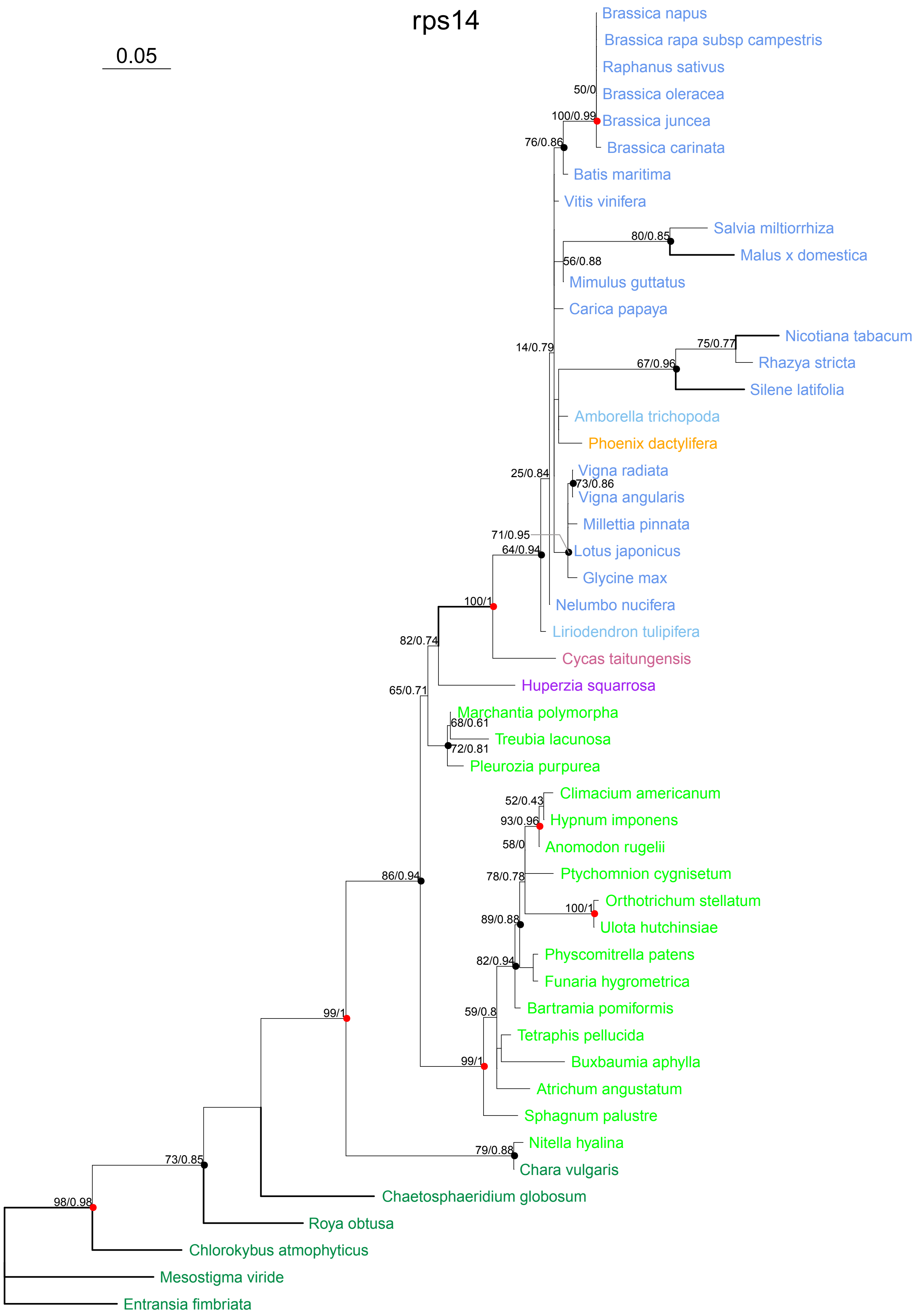

Figure S6 (continued)

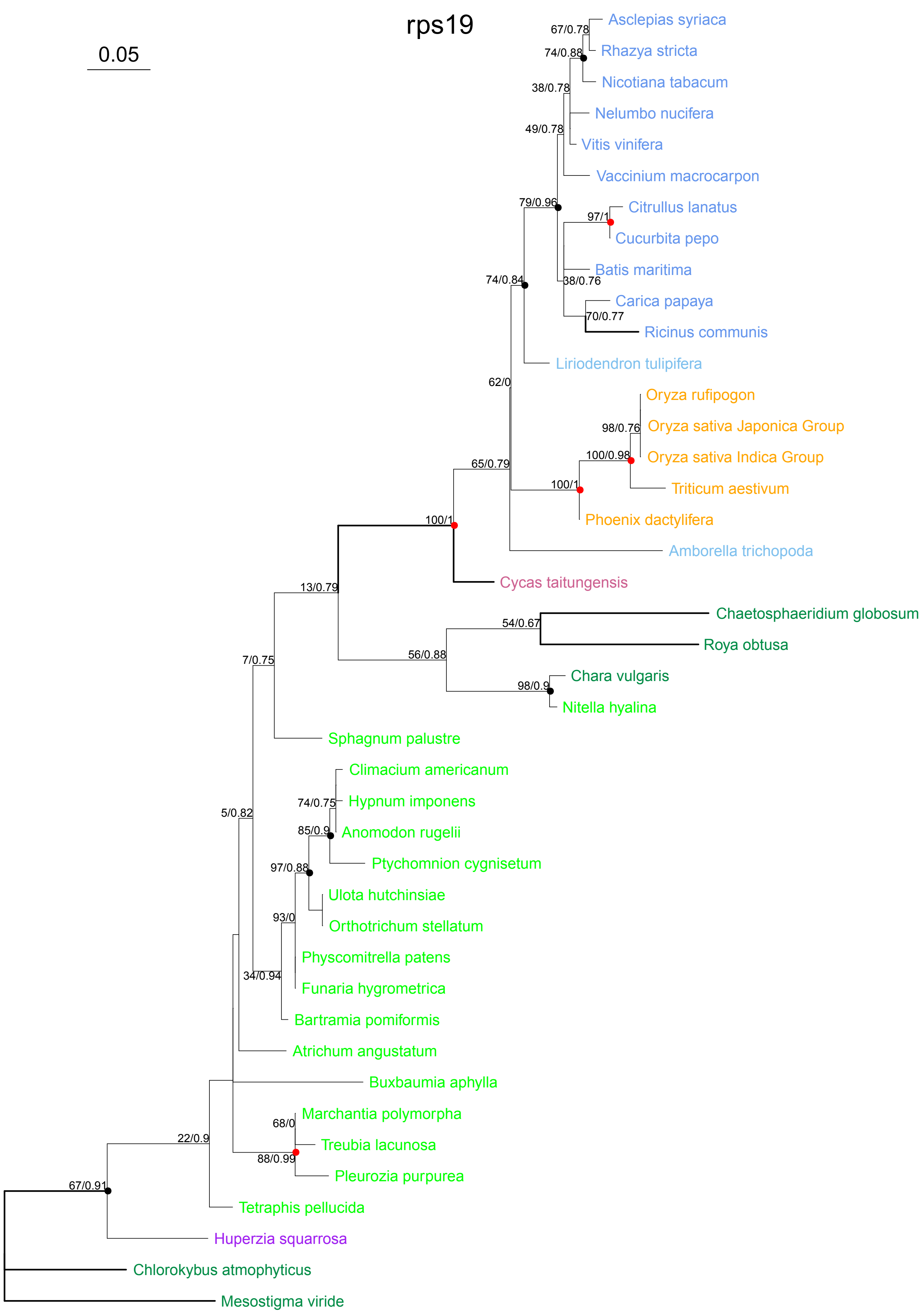

Figure S6 (continued)

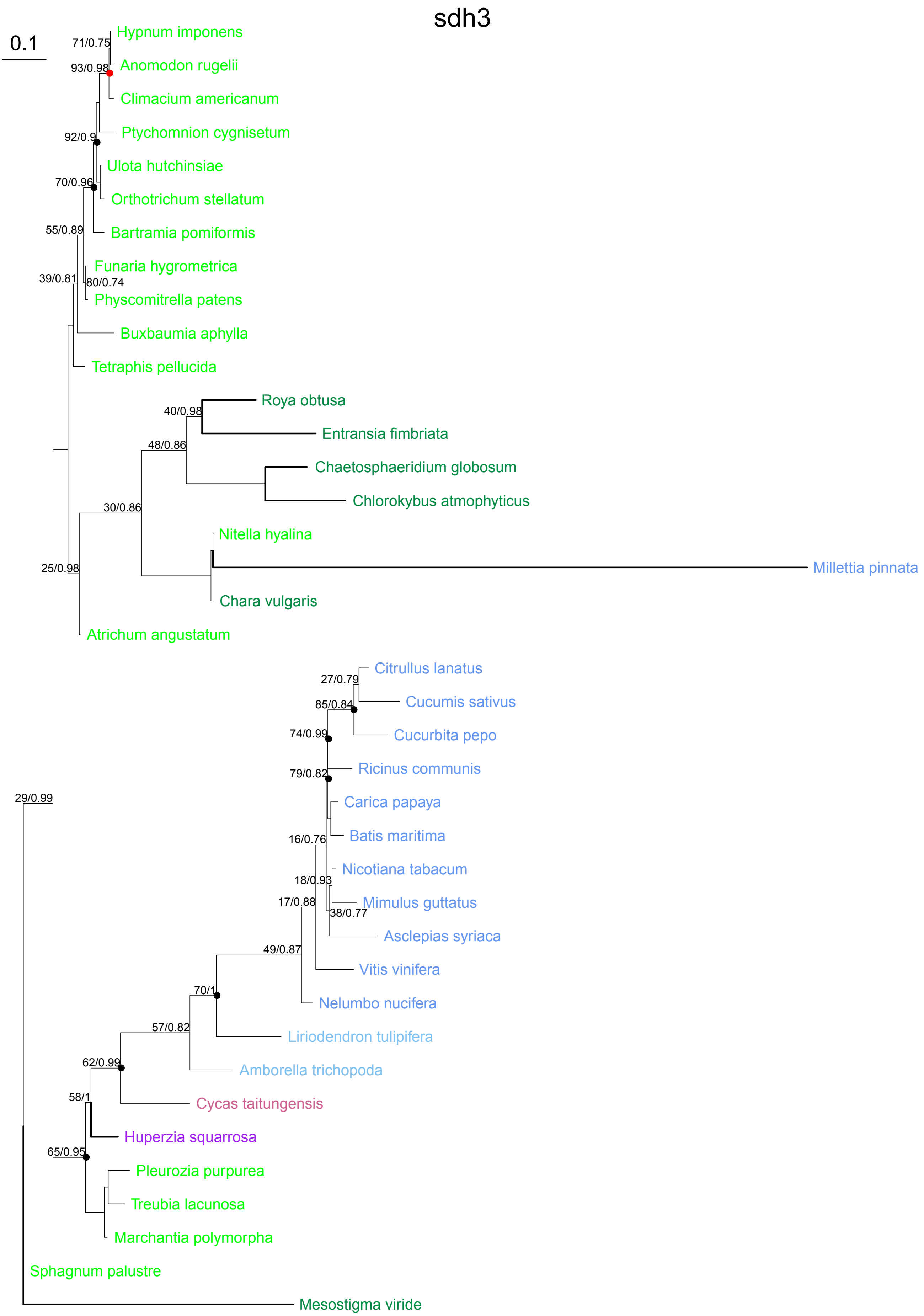

Figure S6 (continued)

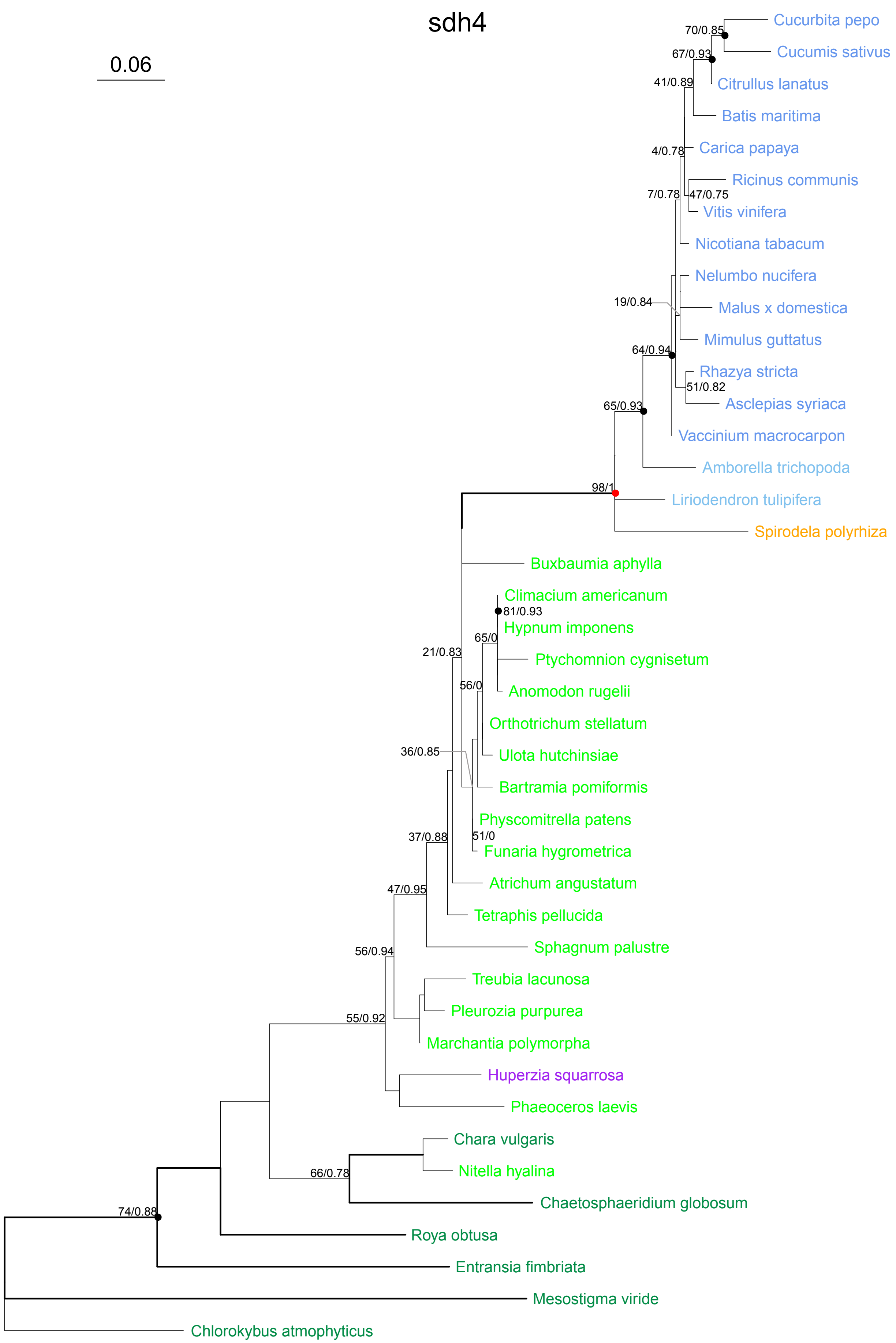

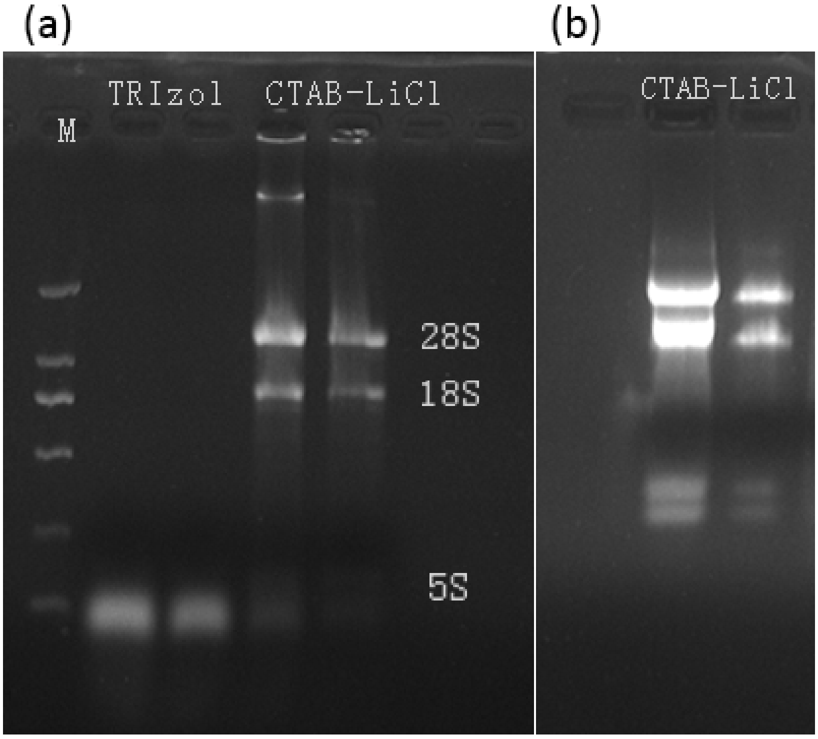

Figure S7. Total RNA isolation from *N. nucifera* using different methods. (a) Comparison of RNA isolation using TRIzol and CTAB-LiCl methods. (b) The DNA-free RNA isolated using the CTAB-LiCl method.

Table S1. Primers used for verification of the assembly.

Primers	Sequences	Position	Length of Sanger Result (bp) *	Mismatch/ Indel
NnMt01F	CGCGCTAGCCTATCACATCT	289866-289885	1,162	0
NnMt01R	TCGATTTCGATGCCCCGATT	291118-291099		
NnMt02F	GGCCTGTCATCGAAGGATGT	20046-20065	1,184	0
NnMt02R	AAAGCGACGTGTGTAGCTGA	21326-21307		
NnMt03F	GGTGGCACAAAAGCGTACAG	40063-40082	1,042	0
NnMt03R	GGGAAGTGAAGGCGGAATGA	41196-41177		
NnMt04F	CTTTCCCCGATCGACCTAGC	50069-50088	1,226	0
NnMt04R	GATGGGAACGGAGGCTGAAA	51389-51370		
NnMt05F	TGTCGTATGCCCTTGCCATT	60038-60057	1,367	0
NnMt05R	GCTTCAACGGCAGCTCAAAA	61497-61478		
NnMt06F	CCACAATCAAGGTGGCAGGA	67011-67030	1,262	1 mismatch: A-T at position 68,132
NnMt06R	GCATGCAGTACCCGAGTCTT	68370-68351		
NnMt07F	GCAAAGCTCGCAACTAGACG	110176-110195	999	0
NnMt07R	AGTTTTCTCGCTTGCTCGGA	111271-111252		
NnMt08F	CTATTCGGGGAGGACTTCAGC	130035-130055	1,124	0
NnMt08R	ACCGTCAAGCGCTAGTGAAA	131256-131237		
NnMt09F	TCTTGGAGGCCCCAAAATCG	150061-150080	960	0
NnMt09R	TGGTGGGTGGGGTAGGTAAA	151120-151101		
NnMt10F	TCGGTTGGACATTGGACTCG	230071-230090	1,214	0
NnMt10R	CGGGAACAAAAGGAACAGCG	231384-231365		
NnMt11F	ATGGCATGGCAGTAAGCAGT	245112-245131	1,046	0
NnMt11R	GTAGTGGTGCTTCGACCACA	246259-246240		
NnMt12F	CGAGAGAGCAGTGGATGGAC	260129-260148	1,151	0
NnMt12R	ACCCGGTTTGTTTGCATTCG	261382-261363		
NnMt13F	GCTTGTGCGCTTATTGCCTT	360134-360153	1,265	0
NnMt13R	GACACTCTCCGGAGCCTAGA	361487-361468		
NnMt14F	AGCTTCCATCAACGCTCCAA	390058-390077	927	0
NnMt14R	TAAAGATAGGCCGCTCTGCG	391082-391063		
NnMt15F	AGGATCAAACCGCTGCTGAA	410021-410040	1,056	0
NnMt15R	GCTACCCCGAGGCGAAAATA	411176-411157		
NnMt16F	AAGTGACGGCTTTAGGCGAA	440089-440108	907	0
NnMt16R	GCCGCCTTTGGTTGAATGTT	441089-441070		
NnMt17F	GCCTTAGTTCTTCCCCGCTT	465117-465136	1,025	0
NnMt17R	CATCGCTCGGGATCAGAGAC	466239-466220		
NnMt18F	TCGCTCCTCCATCCAACCTA	500059-500078	1,259	0
NnMt18R	AGCCAATAAGCTGGGGACAC	501408-501389		
**Total**			**20,176**	**1**

* PCR products were directly sequenced by ABI3730 sequencer, the primers (20 bp) and bases immediately following the primers (nearly 30 bp) were unable to be clearly sequenced, which makes the sequencing results nearly 100 bp shorter than PCR products.

Table S2. GC contents in whole genome and gene-coding regions of 79 plant mitochondrial genomes.

Species	Whole genome			Gene coding regions		
	**GC%**	**GC No.**	**Length (bp)**	**GC%**	**GC No.**	**Length (bp)**
Brassica juncea	45.24%	99414	219766	44.19%	22327	50526
Brassica rapa subsp. campestris	45.24%	99413	219747	44.19%	22327	50526
Brassica oleracea	45.20%	162839	360271	44.13%	21361	48408
Brassica napus	45.19%	100246	221853	44.09%	22560	51165
Brassica carinata	45.33%	105268	232241	44.08%	20526	46569
Raphanus sativus	45.21%	116844	258426	42.62%	13396	31434
Arabidopsis thaliana	44.77%	164270	366924	44.14%	30431	68940
Batis maritima	45.41%	183433	403930	44.49%	27232	61209
Carica papaya	45.12%	215159	476890	43.18%	14669	33972
Ricinus communis	44.98%	226159	502773	42.46%	13333	31398
Vigna angularis	45.19%	182769	404466	43.23%	10078	23313
Vigna radiata	45.11%	181015	401262	42.98%	12461	28992
Glycine max	45.03%	181275	402558	44.52%	24650	55365
Millettia pinnata	45.00%	191586	425718	42.91%	14660	34164
Lotus japonicus	45.40%	172892	380861	43.09%	13508	31347
Cucurbita pepo	42.80%	420617	982833	43.24%	13941	32244
Cucumis sativus	44.27%	688858	1555935	43.26%	14079	32547
Citrullus lanatus	45.08%	170955	379236	43.10%	14026	32544
Malus x domestica	45.39%	180170	396947	43.08%	12723	29532
Ajuga reptans	45.10%	158773	352069	44.14%	12228	27702
Salvia miltiorrhiza	44.39%	221599	499236	43.32%	33098	76401
Mimulus guttatus	45.14%	237296	525671	42.30%	14979	35412
Boea hygrometrica	43.27%	220910	510519	41.68%	13034	31272
Daucus carota subsp. sativus	45.42%	127686	281132	44.43%	26105	58752
Helianthus annuus	45.05%	135568	300945	43.45%	9802	22560
Rhazya stricta	43.68%	239616	548608	42.41%	16753	39504
Asclepias syriaca	43.43%	296387	682498	42.51%	13138	30906
Nicotiana tabacum	44.96%	193590	430597	44.50%	37108	83388
Vaccinium macrocarpon	45.33%	208393	459678	42.72%	11702	27390
Beta vulgaris subsp. maritima	43.91%	160240	364950	42.57%	42856	100683
Beta vulgaris subsp. vulgaris	43.86%	161748	368801	42.60%	37957	89100
Beta macrocarpa	43.89%	169084	385220	42.60%	43533	102195
Silene latifolia	42.56%	107849	253413	40.68%	12361	30387
Silene vulgaris	41.80%	178548	427138	**45.44%**	18856	41492
Vitis vinifera	44.14%	341361	773279	42.45%	21005	49479
Nelumbo nucifera	**48.16%**	252718	524797	**46.12%**	22130	47979
Zea luxurians	43.93%	236964	539368	43.09%	13711	31818
Zea perennis	43.88%	250299	570354	42.88%	13441	31347
Zea mays subsp. mays	43.93%	250256	569630	43.31%	42864	98976

Table S2. (continued)

Zea mays subsp. parviglumis	43.88%	298643	680603	43.64%	16504	37818
Tripsacum dactyloides	43.93%	309277	704100	43.36%	15098	34818
Sorghum bicolor	43.73%	204917	468628	42.79%	13423	31368
Oryza rufipogon	44.04%	246189	559045	44.12%	14372	32574
Oryza sativa Japonica Group	43.85%	215104	490520	43.86%	19659	44826
Oryza sativa Indica Group	43.84%	215466	491515	43.64%	20077	46008
Triticum timopheevii	44.35%	196659	443419	42.72%	14563	34089
Triticum aestivum	44.35%	200705	452528	42.40%	15307	36099
Aegilops speltoides	44.43%	211504	476091	42.76%	14075	32913
Phoenix dactylifera	45.14%	322781	715001	44.12%	16879	38253
Butomus umbellatus	**49.10%**	221355	450826	44.97%	12770	28395
Spirodela polyrhiza	45.70%	104423	228493	43.27%	13320	30780
Liriodendron tulipifera	**47.70%**	264146	553721	44.49%	15748	35397
Amborella trichopoda	45.92%	1775336	3866039	**48.99%**	37895	77354
Cycas taitungensis	46.92%	194668	414903	45.30%	15625	34494
Huperzia squarrosa	44.16%	182632	413530	43.40%	30642	70599
Phaeoceros laevis	44.60%	93438	209482	38.79%	9401	24237
Hypnum imponens	40.91%	42481	103830	36.30%	11743	32346
Anomodon rugelii	41.20%	42948	104239	37.77%	14249	37728
Climacium americanum	41.05%	43124	105048	36.46%	11799	32361
Ptychomnion cygnisetum	42.07%	43950	104480	37.56%	11928	31761
Orthotrichum stellatum	39.84%	41482	104131	35.48%	11471	32328
Ulota hutchinsiae	39.80%	41632	104608	35.46%	11462	32325
Bartramia pomiformis	39.05%	41473	106198	34.92%	11290	32328
Physcomitrella patens	40.57%	42734	105340	36.45%	13012	35703
Funaria hygrometrica	40.39%	44267	109586	35.70%	11544	32337
Buxbaumia aphylla	40.08%	40367	100725	36.30%	11286	31095
Tetraphis pellucida	42.53%	45818	107730	37.99%	13200	34746
Atrichum angustatum	40.51%	46647	115146	35.90%	11619	32367
Sphagnum palustre	38.99%	55088	141276	35.15%	11408	32457
Marchantia polymorpha	42.41%	79139	186609	40.66%	25708	63225
Pleurozia purpurea	45.37%	76462	168526	41.42%	18282	44139
Treubia lacunosa	43.38%	65928	151983	41.57%	21913	52710
Nitella hyalina	40.83%	32744	80193	38.03%	15828	41625
Chara vulgaris	40.90%	27704	67737	37.68%	14884	39504
Roya obtusa	26.83%	18637	69465	27.22%	8881	32622
Chaetosphaeridium globosum	34.41%	19466	56574	31.84%	10016	31455
Entransia fimbriata	42.70%	26325	61645	36.28%	10019	27618
Chlorokybus atmophyticus	39.80%	80298	201763	36.24%	17387	47976
Mesostigma viride	32.21%	13666	42424	31.31%	8694	27771

* The top three GC content percentage in each column are highlighted in bold

Table S3. The SSR sites identified in the *N. nucifera* mitochondrial genome.

SSR number	SSR type	SSR motif	size	start	end
1	p5	(GCGTA)3	15	5709	5723
2	p5	(AGCGA)3	15	5728	5742
3	p3	(AAC)4	12	5993	6004
4	p2	(TC)5	10	7867	7876
5	p4	(TAAT)3	12	7952	7963
6	p4	(GGCG)3	12	10892	10903
7	p4	(GAAA)3	12	11196	11207
8	p4	(CTTC)3	12	15712	15723
9	p5	(TCCTT)3	15	15964	15978
10	p4	(CTTC)3	12	16019	16030
11	p4	(TAGT)3	12	17181	17192
12	p5	(CAGTC)3	15	22845	22859
13	p2	(AG)5	10	23304	23313
14	p3	(CGC)4	12	28404	28415
15	p4	(GTTC)3	12	31082	31093
16	p4	(AGAC)3	12	31599	31610
17	p4	(CTTT)3	12	35249	35260
18	p4	(GTTC)3	12	36888	36899
19	p4	(GAAA)3	12	37046	37057
20	p4	(ATTC)3	12	40480	40491
21	p3	(GAT)4	12	47607	47618
22	p4	(TAGG)3	12	47819	47830
23	p3	(TTC)6	18	53520	53537
24	p4	(ATTA)3	12	55620	55631
25	p2	(AG)5	10	55706	55715
26	p3	(GTT)4	12	57579	57590
27	p5	(CTTCG)3	15	57839	57853
28	p5	(GCTAC)3	15	57858	57872
29	p4	(TTCC)3	12	64035	64046
30	p5	(TAGTA)3	15	64975	64989
31	p3	(CTA)4	12	67584	67595
32	c*	(ATA)5(AT)5*	22	67904	67925
33	p2	(TA)5	10	67962	67971
34	p2	(AT)5	10	67996	68005
35	p3	(ATA)4	12	68037	68048
36	p2	(TA)6	12	68063	68074
37	p3	(ATT)7	21	68111	68131
38	p1	(T)11	11	69654	69664
39	p5	(CTAGT)3	15	72558	72572
40	p4	(AATG)5	20	73134	73153
41	p6	(AGTGGA)3	18	78248	78265
42	p5	(GATGA)3	15	89952	89966
43	p2	(TA)6	12	90564	90575
44	p4	(TTCC)3	12	92607	92618
45	p5	(GGCCT)3	15	97282	97296
46	p4	(GAGC)3	12	100719	100730
47	p4	(GCTA)3	12	101821	101832
48	p4	(GGCC)3	12	104632	104643
49	p1	(T)11	11	104811	104821
50	p6	(AGAATA)3	18	104823	104840
51	p2	(AC)5	10	112192	112201
52	p1	(A)10	10	121227	121236
53	p1	(G)10	10	122197	122206
54	p1	(T)13	13	122724	122736
55	p1	(T)14	14	126309	126322
56	p6	(TTTCTA)6	36	127204	127239
57	p4	(GGCT)3	12	129078	129089
58	p4	(GAAA)3	12	129768	129779

Table S3. (continued)

59	p1	(A)10	10	130021	130030
60	p5	(TGGTT)3	15	132922	132936
61	p5	(CCTCT)3	15	137178	137192
62	p2	(TC)5	10	139250	139259
63	p4	(TATC)3	12	149305	149316
64	p4	(ATAA)3	12	153101	153112
65	p1	(A)10	10	153360	153369
66	p5	(ACAAA)3	15	153591	153605
67	p4	(GATA)3	12	154157	154168
68	p1	(T)10	10	154578	154587
69	p4	(AATC)3	12	159205	159216
70	p4	(GCCT)3	12	159599	159610
71	p4	(GGTA)3	12	164153	164164
72	p2	(TA)9	18	164779	164796
73	p4	(AGAA)3	12	164975	164986
74	p4	(GATT)3	12	170366	170377
75	p1	(A)13	13	170673	170685
76	p5	(GCGTA)3	15	175418	175432
77	p5	(AGCGA)3	15	175437	175451
78	p3	(AAC)4	12	175702	175713
79	p2	(TC)5	10	177576	177585
80	p4	(TAAT)3	12	177661	177672
81	p3	(AAG)6	18	179753	179770
82	p4	(TTAT)3	12	181362	181373
83	p1	(A)12	12	181463	181474
84	p1	(C)11	11	183465	183475
85	p4	(CAGC)3	12	185557	185568
86	p2	(CT)5	10	188771	188780
87	c	(T)11(G)10	21	193656	193676
88	p2	(AG)7	14	195622	195635
89	p2	(TG)5	10	196829	196838
90	p3	(CTC)5	15	202014	202028
91	p6	(AGAAAT)3	18	202393	202410
92	p2	(AT)5	10	202460	202469
93	p2	(CT)5	10	209274	209283
94	p5	(CCAAC)3	15	214712	214726
95	p3	(CTT)4	12	216983	216994
96	p1	(A)15	15	217263	217277
97	p4	(GGGA)3	12	219175	219186
98	p3	(ATG)4	12	220136	220147
99	p1	(T)16	16	224677	224692
100	p4	(AACA)3	12	225071	225082
101	p4	(AAAG)3	12	240213	240224
102	p4	(ATCT)3	12	242644	242655
103	p4	(ACTG)3	12	247120	247131
104	p4	(GACC)3	12	247677	247688
105	c	(AT)5(AAATAA)3	28	270952	270979
106	p4	(CGGC)3	12	271357	271368
107	p4	(ACAA)3	12	275595	275606
108	p5	(CGCTT)3	15	277934	277948
109	p2	(CT)5	10	278343	278352
110	p4	(CTTG)3	12	278901	278912
111	p2	(GA)5	10	282035	282044
112	p2	(CA)5	10	282417	282426
113	p4	(TAGC)3	12	282863	282874
114	p4	(TATC)3	12	283950	283961
115	p5	(CTATA)3	15	288367	288381
116	p4	(GGAA)3	12	289313	289324
117	p5	(GCGTA)3	15	295485	295499
118	p5	(AGCGA)3	15	295504	295518
119	p3	(AAC)4	12	295769	295780

Table S3. (continued)

120	p2	(TC)5	10	297641	297650
121	p4	(TAAT)3	12	297726	297737
122	p4	(GGCG)3	12	300659	300670
123	p4	(GAAA)3	12	300961	300972
124	p4	(CTTC)3	12	305472	305483
125	p5	(TCCTT)3	15	305723	305737
126	p4	(CTTC)3	12	305778	305789
127	p4	(TAGT)3	12	306940	306951
128	p4	(TGTT)3	12	309065	309076
129	p5	(CAGTC)3	15	312580	312594
130	p2	(AG)5	10	313039	313048
131	p3	(CGC)4	12	318141	318152
132	p4	(GTTC)3	12	320819	320830
133	p1	(G)11	11	322854	322864
134	p1	(T)11	11	324730	324740
135	p5	(CATGG)3	15	330030	330044
136	p4	(TTTA)3	12	336196	336207
137	p4	(TCAG)4	16	342373	342388
138	p4	(TTCC)3	12	342400	342411
139	p4	(GACT)3	12	345847	345858
140	p4	(TTCT)3	12	351317	351328
141	p5	(TATAC)4	20	353793	353812
142	p4	(AAAG)5	20	354767	354786
143	p4	(TTTC)3	12	357135	357146
144	p2	(AG)5	10	368743	368752
145	p2	(TA)6	12	369498	369509
146	p4	(GTTT)4	16	369886	369901
147	p5	(ATAAG)3	15	370603	370617
148	p4	(GGAT)3	12	372541	372552
149	p4	(ATAC)3	12	372595	372606
150	p4	(AATG)3	12	374944	374955
151	p3	(CTC)5	15	381750	381764
152	p3	(TGC)4	12	383427	383438
153	p4	(AAAG)3	12	386627	386638
154	p5	(TTCCT)3	15	388165	388179
155	p2	(TC)6	12	391666	391677
156	p1	(G)14	14	396079	396092
157	p4	(GATT)3	12	397050	397061
158	p1	(T)17	17	409052	409068
159	p5	(TCTAC)3	15	410926	410940
160	p4	(TCTA)5	20	411210	411229
161	p5	(CTTTG)3	15	412972	412986
162	p2	(AG)6	12	419112	419123
163	p4	(CTTA)3	12	425024	425035
164	p1	(A)10	10	429414	429423
165	p2	(AT)6	12	431699	431710
166	p4	(GCCC)3	12	438917	438928
167	p4	(GGAA)3	12	440601	440612
168	p1	(T)10	10	449121	449130
169	p5	(TACTA)4	20	449470	449489
170	p4	(CCTT)3	12	451100	451111
171	p1	(G)11	11	454531	454541
172	p6	(ATCTAT)3	18	457210	457227
173	p4	(TCAT)3	12	457389	457400
174	p4	(TTCT)3	12	457574	457585
175	p1	(T)10	10	459544	459553
176	p1	(T)10	10	470235	470244
177	p4	(AGAT)3	12	471353	471364
178	p4	(AATA)4	16	471474	471489
179	p3	(ATT)5	15	471902	471916
180	p1	(A)12	12	472200	472211

Table S3. (continued)

181	p1	(T)10	10	472654	472663
182	p4	(CCAG)3	12	474591	474602
183	p2	(AG)5	10	475173	475182
184	p1	(G)12	12	475799	475810
185	p2	(TC)8	16	477336	477351
186	p2	(AG)7	14	477367	477380
187	p3	(GCA)4	12	478577	478588
188	p3	(TGT)4	12	479463	479474
189	p4	(GCAA)3	12	480237	480248
190	p4	(GCAG)3	12	489622	489633
191	p1	(G)11	11	489980	489990
192	p2	(AG)5	10	492660	492669
193	p3	(GAA)4	12	493663	493674
194	p3	(TGA)4	12	493719	493730
195	p1	(G)14	14	495210	495223
196	p1	(A)12	12	501527	501538
197	p3	(TAA)4	12	508454	508465
198	p5	(GTAAT)3	15	509392	509406
199	p2	(AC)6	12	510101	510112
200	p4	(TCGC)3	12	515551	515562
201	p4	(TTCT)3	12	523789	523800

Table S4. Relative synonymous codon usage for the protein-coding mitochondrial genes of *Nelumbo nucifera*.

Codon	Count[1]	RSCU[2]	Codon	Count	RSCU	Codon	Count	RSCU	Codon	Count	RSCU
**UUU(F)**	362	1.08	UCU(S)	245	1.37	UAU(Y)	248	1.52	UGU(C)	102	1.28
**UUC(F)**	307	0.92	UCC(S)	184	1.03	UAC(Y)	78	0.48	UGC(C)	57	0.72
**UUA(L)**	239	1.32	UCA(S)	203	1.14	**UAA(*)**[3]	19	1.58	**UGA(*)**[3]	11	0.92
**UUG(L)**	231	1.28	UCG(S)	160	0.9	**UAG(*)**[3]	6	0.5	UGG(W)	155	1
**CUU(L)**	227	1.25	CCU(P)	239	1.4	CAU(H)	224	1.53	CGU(R)	163	1.2
**CUC(L)**	118	0.65	CCC(P)	135	0.79	CAC(H)	69	0.47	CGC(R)	85	0.63
**CUA(L)**	167	0.92	CCA(P)	197	1.15	CAA(Q)	242	1.48	CGA(R)	180	1.33
**CUG(L)**	105	0.58	CCG(P)	112	0.66	CAG(Q)	84	0.52	CGG(R)	97	0.71
**AUU(I)**	374	1.3	ACU(T)	203	1.37	AAU(N)	239	1.3	AGU(S)	175	0.98
**AUC(I)**	251	0.88	ACC(T)	156	1.05	AAC(N)	129	0.7	AGC(S)	105	0.59
**AUA(I)**	235	0.82	ACA(T)	139	0.94	AAA(K)	292	1.17	AGA(R)	195	1.44
**AUG(M)**	292	1	ACG(T)	94	0.64	AAG(K)	208	0.83	AGG(R)	94	0.69
**GUU(V)**	199	1.16	GCU(A)	280	1.58	GAU(D)	259	1.4	GGU(G)	243	1.25
**GUC(V)**	129	0.75	GCC(A)	162	0.91	GAC(D)	110	0.6	GGC(G)	98	0.51
**GUA(V)**	199	1.16	GCA(A)	177	1	GAA(E)	314	1.35	GGA(G)	292	1.51
**GUG(V)**	158	0.92	GCG(A)	90	0.51	GAG(E)	150	0.65	GGG(G)	142	0.73

[1] Count means the numbers of codons used among the protein-coding mitochondrial genes

[2] RSCU represents the relative synonymous codon usage

[3] The codons in bold with asterisks represent stop codons

Table S5 Collinearity analysis to identify conserved gene clusters among the 38 species.

			Monocots									Dicots																										
**Gene clusters**	**Nelumbo**	**Liriodendron**	**Phoenix**	**Oryza**	**Sorghum**	**Tripsacum**	**Zea**	**Aegilops**	**Triticum**	**Butomus**	**Spirodela**	**Ajuga**	**Arabidopsis**	**Asclepias**	**Batis**	**Beta**	**Boea**	**Brassica**	**Carica**	**Citrullus**	**Cucumis**	**Cucurbita**	**Daucus**	**Glycine**	**Helianthus**	**Lotus**	**Malus**	**Millettia**	**Mimulus**	**Nicotiana**	**Raphanus**	**Rhazya**	**Ricinus**	**Salvia**	**Silene**	**Vaccinium**	**Vigna**	**Vitis**
1	rRNA18-1	-	-	88,5	30	54	-	13,40	6,18	26	37	-	129	-	-	-	-	42	-	53	34,8	40	13	80	-	30	-	-	63	41	-	-	3	45	-	-	17	45
	rRNA5-1	-	-	-	-	-	-	-	-	27	38	-	128	-	-	-	3	41	-	52	33	-	12	-	-	31	-	-	61	43	-	-	4	-	-	-	-	46
	rRNA26-2	-	-	80	28	45	-	71,2	1	-	-	-	3	60	79	61	-	-	-	-	30,41	4	-	54	-	35	30	35	29,1	-	-	21	5	-	-	34	23	-
	nad6	28	13	17	-	12	213	18	-	-	-	44	28	-	-	-	65	-	10	12	27	-	-	-	-	40	-	-	-	-	50	27	59	-	9	-	30	-
	rRNA18_b-2	-	-	-	-	-	-	-	-	-	-	-	-	-	-	-	-	-	-	-	-	-	-	-	-	-	-	-	-	-	-	-	-	-	-	-	-	-
	trnD (GTC)_ψ-2	-	-	-	-	-	-	-	-	-	-	-	-	-	-	-	-	-	-	-	-	-	-	-	-	-	-	-	-	-	-	-	-	-	-	-	-	-
	ccmFN-2	-	-	66	21	29	-	38,3	16	-	2	38	-	-	73	17	12	23	61	34	15	21	-	63,107	-	52,15	13	9	64	52	6	1	-	-	12	7	40	23
	nad1_exon4,5	10	12	2	1	8	21	17	4	5	22	2	53	-	16	-	5	38	2	24	4	9	16	-	-	2	16	5	5	60,4	12	24	1	1	23	43	3	3
	matR	-	35	64	-	31	-	36	14	6	-	33	54	-	17	109	61	39	5	33	-	27	17	93	-	5	35	-	13	-	13	23	22	5	24	-	-	-
2	nad4L	14	19	61	40	38	-	30	70	-	34	31	67,7	68	30	-	16	28	-	61	61	22	77	57	6	17	-	16	47	-	-	3	30	-	47	12	-	59
	atp4	15	18	4	45	15	64	64	35	46	35	30	-	67	49	-	-	27	-	60	14	23	-	22	-	16	61	17	46	-	-	2	31	-	46	13	-	60
	ccmC	68	-	20	27	7	84	42	29	37,11	39	22	92	18	44	22	23	99	7	59	43	24	-	2	-	62	-	44	53	11	45	13	32	-	10	-	37	69
	sdh3-b	26	-	-	-	-	-	-	-	-	-	-	-	8	-	-	-	-	12	40,7	-	26	-	-	-	-	22	-	59	30,182,133	-	-	-	-	-	-	-	36
3	nad1_exon2,3	10	12	2	1	8	21	17	4	5	22	2	53	-	80	-	5	38	2	24	4	9	16	-	-	2	16	5	5	60,4	12	39,71	1	1	23	43	3	3
	rps13	-	-	5	16	9	-	21	6	-	-	24	-	61,29	1	-	-	-	16	55	46	10	32	-	-	-	39	-	33	61	-	70	33	89	-	9	-	83
	cox2	32	31	45	47	33	207	8	42	-	59	17	120,17	36	65	80,106	-	44,97	3	23	58	14	-	97,97	-	64	59	19	20	70	-	44	50	-	-	44	-	-
	rps4	70	27	51	-	32	8	19	48	-	43	-	30	51	4,12	88	-	-	11	11	-	43	78	64	-	32	-	47	49	-	43	26	60	68	-	17	34	-
	rps7	-	-	12	24	-	-	48	62	14	-	-	119	3	5	104,4	-	32	15	48	9	-	79	-	-	63	-	-	-	-	15	22	-	59	-	-	-	43
	atp1_ψ	7	21	42	2	35,13	190	3	-	15	51	-	116	17	-	-	-	-	-	-	52	20	37,19	99,48,68	-	33	25	12	4	-	38	-	56	10	29	45	-	-
	rRNA18_ψ-1	62	-	58	30	-	184	-	66,18	39	37	-	-	-	21	-	-	42	27	53	8	40	13	80	-	-	43	-	63	41	33	-	3	-	42	-	17	45
	atp6	31	2	48	48	26	-	22	21	21	40	1	115	47	75	96	6	25	-	6	49	18	5	101,27	43	21	49	51	34,22	-	8	76	43	-	-	-	31	47
	rps2	-	26	-	10	-	182	6	40	-	49	-	-	-	-	-	-	-	-	-	-	-	-	-	-	-	-	-	-	-	-	-	-	-	-	-	-	-
	trnL(CAA)_ψ	-	-	-	-	-	-	-	-	-	-	-	-	-	-	-	-	-	-	-	-	-	-	-	-	-	-	-	-	-	-	-	-	-	-	-	-	-
	ccmFC	50	20	67	-	21	36	-	25	25	48	32	-	37,60	31	18	51	48	4	44	3	32	29	26	-	37	1	-	42	139	27	65	21	95	31	-	7	30
4	cox1	41	8	72	37	-	159	46	44	-	1	-	-	25	32	108	66	83	24	1	2	2	80	24	19	1	4	2	40	53	-	60	18	119	1	2	-	40
	rps10	2	-	-	-	-	-	-	-	-	-	-	-	72	33	-	-	-	25	-	65	55	-	23	-	65	-	18	41	54	-	59	17	51	-	10	52	94
	rps19-2	66	-	60	-	-	-	29	71	-	-	-	-	52	24	-	-	-	6	3	-	54	-	-	-	-	-	-	-	67	-	41	39	-	-	11	-	-
	rps1	12	23	65	20	30	-	37	15,1	38	6	-	-	30	64	-	-	-	60	-	53	53	45,90	65	-	-	27	70	-	83	-	40	55	-	-	-	15	98
	mttB_ψ	-	-	-	-	-	-	-	-	-	-	-	-	-	-	-	-	-	-	-	-	-	-	-	-	-	-	-	-	-	-	5	-	-	-	-	-	-
5	nad5_exon4,5	34	32	-	18	43	87	15	-	3	13	-	7	11	41	-	-	21,60,21	9	43	1	5	1	66	20	-	7	23	6	-	4	73	2	15	11	1	1	1
	trnE(TTC)	35	-	79	39	27	19	63	-	30,36	-	16	-	39	55	41	26	-	51	-	59	15	-	-	-	42	-	58	56	150	-	30	58	-	33	53	9	52
	nad7	36	9	27	23	34	101	31	69	-	14	3	52	-	14	100	64	1	21	56	20	17	55	47	-	4	-	69	-	165	-	6	16	-	37	56	46	24,48
6	nad5_exon1,2	34	32	-	18	43	87	15	-	3	13	-	7	11	15	-	-	21,60,21	9	43	1	5	1	66	20	-	7	23	6	-	4	18	2	15	11	1	1	1
	atp9	22	22	55	51	40	-	-	3	-	32	7	110	6,31	6	30	31	59	58	42	48	19	11	-	21	-	12	-	7	62	31,16	19	19	143	25	26	14,45	-
	trnG (GCC)	-	-	-	-	-	-	-	-	-	-	-	-	-	77	-	-	-	-	-	-	-	-	-	-	-	-	-	-	-	-	61	-	-	-	-	-	-
	nad9	46	40	-	-	20	40	5	38	9	-	-	8	13	29	-	-	22	54	41	54	52	-	76	41	44	45	29	37	153	5	64,78	15	21	7	-	32	67
	ccmFN-1	43	29	66	21	29	-	3,38	16	43	2	38	85,98	66	73	-	50	58,23	31	34	15	21	36	107,63	-	52	13	9	64	52	30,6	1	-	155	12	7	40	-
	trnD (GTC)_ψ-1	-	-	-	-	-	-	-	-	-	-	-	-	-	-	-	-	-	-	-	-	-	-	-	-	-	-	-	-	-	-	7	-	-	-	-	-	-
	rRNA18-2	62	-	-	30	-	-	68,50,40	18,53,66	26	-	-	-	-	21	-	-	42,42	27	-	34	40	13	80	-	30	43	67	63	41	33	54	3	45	-	-	17	-
	rRNA5-2	-	-	-	-	-	-	-	-	-	-	-	-	-	-	-	-	-	-	-	-	-	-	-	-	-	-	-	-	-	-	55	-	-	-	-	-	-
	rRNA26-1	29	-	80	28	45	-	71,2	45,61	23	45	-	3	64	79	61,98	49	65	57	29	41,30,41	4	98	54	23	-	52,30	35	1	-	37	21	5	32	36	34	23	-
	nad2_exon1,2	1	-	85	34	16	141,47,23	-	-	1	3	12	7,1	1	45	32	7	-	-	17	29	1	38	-	-	-	23	1	6	92,120	44	14	26	58	16	-	13	28
7	rps12	56	43	40	9	50	139,108,27	59	58	47	23	42	100,53	69	57	120	57	62	33	22	23	51	43,108	21	40	61	50,20	53	-	34	34	8	51,51	-	-	20	42	99
	nad3	55	42	39	8	49	109	58	59	-	24	11	101	70	56	-	56	-	32	21	22	50	44,109	20	39	60	21	52	65	33	35	7	52,52	-	20	21	43	100
8	rpl16	64	16	38	52	5	70	1,33	22	-	58	-	9	54	35	-	42	-	39	5	24	31	53	87	37	6	-	46	66	-	21	43	41	66	-	15	4	-
	rps3	65	-	37	-	-	71,1	3	6	-	-	43	10	53	36	123	43	-	38	4	-	-	-	-	36	7	56	45	-	-	20	42	-	67	19	-	-	29
	rps19-1	66	15	-	-	-	-	29	71	-	-	-	-	52	37	-	-	-	6	3,62	-	54,29	-	-	-	-	-	-	-	67	-	41	39	-	-	-	-	79
	rpl2	67	14	-	-	-	-	-	-	-	-	-	58	10	34.68,11	-	-	74	37	2	39	28	21	-	-	-	-	32,24	-	162	-	28	-	-	-	16	-	102
	atp1	-	21	90,42	2	13,35	-	75	-	15	-	50	116	17	38	-	12,39	72	8	25	52	20	19,37	99,48,68	34	33	25	12	4,4	-	38	63	56	41	-	45	41	-
	trnC (GCA)	-	-	-	-	-	-	-	-	-	-	-	-	-	-	-	-	-	-	-	-	-	-	-	-	-	-	-	-	-	-	-	-	-	-	-	-	-
9	trnN (GTT)-cp	-	-	-	-	-	-	-	-	-	-	-	-	57	47	36	-	-	35	-	-	-	-	18	2	-	-	39	31	-	-	16	37	-	27	-	25	-
	trnY (GTA)	61	-	77,94	-	19	41	4	37	10	4	13	40	-	46	-	-	86	34	18	37	47	75	19	-	18	-	38	30	-	46	15	36	-	26	29	26	122
10	atp8	3	-	15	29	37	96	60	43,57	31	11	-	49	14	40	136	38	30	43	14	55	44	85	104	7	57	55	22	12	123	41	51	49	162	4	30	44	95
	cox3	4	25	3	-	41	-	62	10	32	9	-	-	15	51	-	37	51	44	15	56	45	52	8	8	56	-	21	-	124	-	50	48	-	3	31	-	96
	sdh4	5	-	-	-	-	-	-	-	-	8	-	-	16	52	-	-	-	45	16	57	46	-	-	-	55	-	20	10	125	-	49	47	-	2	32	48	97
	rps11	-	28	5,52	-	-	-	-	-	-	-	-	-	-	-	-	-	-	-	-	-	-	-	-	-	-	-	-	-	-	-	-	-	-	-	-	-	-
	trnI (CAT)	20,54,45,13	-	14	50,32	10	132,46	25	75	-	16	-	124	65,32	43	92,40	54,48	26	20,28,40	10,30	-	3,11,38	71	-	14	41	2,31	59	27,2,21	131	9	20	53,6	-	35	39,47	-	139
	nad1_exon1	10	12	2	1	8	21	17	4	5	22	2	53	-	69	-	5	38	2	24	4	9	16	-	-	2	16	5	5	60,4	12	23	1	1	23	43	3	3
11	ccmB	-	33	83	43	24	149	66	-	22	47	19	12	21	66	73	32	63	23	37	21	37	92,112	44	12	53	10	-	23	160	17	45	25	166	41	-	29	101
	trnK (TTT)	49	-	25	-	4	-	24,73,54	47,76	-	25	-	72	12	67	78	33	90	22	36	-	36	60	28	44,11	58	11	49,30	68	158	19	53	24	-	-	-	33	72
	trnQ (TTG)	16	-	11,34	38	28	118	72	46	28	20	21	-	28,28	76	144	59	24	30	8,39	31	25	-	33	-	27	34	13	69	105	7	62	10	72	-	51	38	-
	rpl10	71	-	-	-	-	-	-	-	-	26	-	-	20	18	-	-	-	-	-	18	-	66	-	13	-	-	-	24	161	-	46	23	167	-	18	-	32
	petN-cp	-	-	-	-	-	-	-	-	-	-	-	-	-	-	-	-	-	-	-	-	-	-	-	-	-	-	-	-	-	-	-	-	-	-	-	-	-
	trnD (GTC)-cp	19	-	-	-	-	-	-	-	-	-	6	-	24	22	-	19	88	50	-	17	-	-	38	10	24	6	55	36	77	18	57	34	-	-	-	-	156
	trnT (GGT)-cp	-	-	-	-	-	-	-	-	-	-	-	-	-	-	-	-	-	-	-	-	-	-	-	-	-	-	-	-	-	-	-	-	-	-	-	-	-
	nad4	11	-	44	49	55	-	-	28	34	7	5	60	71	23	-	20	76	49	-	64	13	26	36	-	23	5	54	44	78	51	56	35	-	-	46	-	160
	trnS (TGA)	-	-	54	-	44	-	32	68	-	27	-	-	26	10	-	60	81	59	-	-	-	35	-	-	-	-	-	15	-	49	48	44	-	-	-	-	76
	trnH (GTG)-cp	-	-	8	-	-	152	-	-	35	41	-	-	-	54	-	25	18	-	-	51,10	16,16	-	72	17	26	15	31	55	152	3	29	57	-	32	52	36	-
12	trnS (GCT)	23	-	68	-	47	111	57	60	-	53	-	80	40	9	-	27	17	17	-	63	-	96,116	4	-	47	-	65	57	149	2	31	9	37	-	24	-	-
	trnF (GAA)	24	-	-	-	-	-	-	-	-	54	36	-	-	8	156	28	-	-	50	-	-	-	-	29	46	41	64	-	148	-	32	8	36	-	-	27	-
	trnP (TGG)	25,60,38	-	1	41	36	55	44	27	-	55	-	36	42	7	-	29	11	-	49	-	34	114,49,94	7	-	-	40	63	-	146,154	55	33	7,14	35	48	-	-	110,64
	sdh3-a	26	-	-	-	-	-	-	-	-	-	-	-	8	28	-	-	-	12	7,40	28	26	-	-	-	-	-	-	59	182,30	-	10	29	-	-	-	-	36
13	rpl5	53	3	74	-	-	-	61	11	-	28	-	22	9	62	86	-	93	-	45	-	-	111,46,91	-	9	51,14	17	60	19	20	22	36,68	46	-	40	-	21	37
	rps14	52	-	89	-	-	-	-	6	-	29	-	-	-	61	-	-	94	-	-	-	7	-	-	-	13,50	18	61	18	18	23	37,69	-	-	-	-	20	38
	cob	51	-	63	-	-	130	70	20	44	30	-	23,61	35,60	60	84	13	95	46	47	-	8	81	12	31	12	19	62	17	17	24	25	-	-	38	36	19	39
14	trnP (TGG)-cp	25,60,38	-	1	41	36	55	44	27	-	55	-	36	42	7	-	29	11	-	49	-	34	114,49,94	7	-	-	40	63	-	146,154	55	33,75	7,14	35	48	-	-	110,64
	trnW (CCA)-cp	39	-	-	42	-	-	23	-	49	36	47	95	44	70	-	45	-	53	-	13	-	-	81	42	-	47	68	39	155	1	74	13	20	-	-	18	111,65
15	petG-cp_ψ	-	-	-	-	-	-	-	-	-	-	-	-	-	-	-	-	-	-	-	-	-	-	-	-	-	-	-	-	-	-	-	-	19	-	-	-	112
	petL-cp_ψ	-	-	-	-	-	-	-	-	-	-	-	-	-	-	-	-	-	-	-	-	-	-	-	-	-	-	-	-	-	-	-	-	18	-	-	-	113

*The consequent numbers of each species represent the gene collinearity. Blocks of conserved gene clusters were marked as red.

Table S6. RNA editing data obtained from 40 protein-coding genes and one pseudogene in the *Nelumbo nucifera* mitochondrial genome.

Gene	CDS length(bp)	Total edits	Edits/100 nt	Nonsyn[a]	Syn[b]
atp1	1530	13	0.850	12	1
atp4	597	15	2.513	14	1
atp6	774	27	3.488	25	2
atp8	480	7	1.458	5	2
atp9	225	16	7.111	12	4
ccmB	621	43	6.924	40	3
ccmC	759	0	0.000	0	0
ccmFC	1356	0	0.000	0	0
ccmFN	1734	40	2.307	36	4
cob	1182	21	1.777	20	1
cox1	1584	15	0.947	13	2
cox2	783	24	3.065	21	3
cox3	798	20	2.506	20	0
matR	1968	19	0.965	18	1
mttBψ	842	30	3.563	ND[c]	ND[c]
nad1	978	39	3.988	34	5
nad2	1467	45	3.067	39	6
nad3	357	16	4.482	16	0
nad4	1488	68	4.570	65	3
nad4L	303	13	4.290	13	0
nad5	2013	45	2.235	43	2
nad6	633	11	1.738	10	1
nad7	1185	40	3.376	38	2
nad9	573	16	2.792	16	0
rpl16	516	12	2.326	8	4
rpl2	996	3	0.301	2	1
rpl5	561	12	2.139	12	0
rps1	606	7	1.155	7	0
rps10	363	6	1.653	5	1
rps11	444	5	1.126	4	1
rps12	378	0	0.000	0	0
rps13	351	6	1.709	6	0
rps14	303	2	0.660	2	0
rps19	285	5	1.754	4	1
rps2	663	10	1.508	9	1
rps3	1692	17	1.005	14	3
rps4	1059	25	2.361	23	2
rps7	447	3	0.671	3	0
sdh3	375	3	0.800	2	1
sdh4	450	1	0.222	1	0
Total	33719	700	2.076	612	58

[a] Nonsynonymous edits;

[b] Synonymous edits;

[c] No data available, because mttBψ is a pseudogene;

Table S7. RNA editing sites of *Amborella trichopoda*, *Liriodendron tulipifera*, *Nelumbo nucifera*, *Oryza sativa* and *Vitis vinifera*.

gene	Amborella	Liriodendron	Nelumbo	Vitis	Oryza
atp1	11	14	13	8	5
atp4	12	16	15	5	9
atp6	23	29	27	21	16
atp8	7	6	7	8	4
atp9	ND[a]	15	16	4	8
ccmB	49	49	43	6	35
ccmC	41	41	0	18	35
ccmFC	37	24	0[b]	7	27
ccmFN	47	34	40	13	31
cob	26	30	21	25	19
cox1	33	37	15	22	4
cox2	21	14	24	21	19
cox3	21	19	20	10	1
matR	25	19	19	11	ND
mttB	53	58	30 [c]	ND	33
nad1	37	33	39	12	23
nad2	41	49	45	19	30
nad3	19	21	16	5	15
nad4	68	58	68	21	20
nad4L	13	13	13	8	10
nad5	55	48	45	21	11
nad6	20	25	11	9	18
nad7	40	39	40	22	32
nad9	16	14	16	8	12
rpl16	8	9	12	10	12
rpl2	4	3	3	2	1
rpl5	13	12	12	13	1
rps1	3	5	7	7	3
rps10	3	6	6	7	ND
rps11	5	8	5	ND	4
rps12	9	9	0	9	ND
rps13	5	7	6	6	8
rps14	2	2	2	3	ND
rps19	4	5	5	8	6
rps2	ND	ND	10	ND	10
rps3	ND	17	17	10	10
rps4	27	ND	25	14	15
rps7	3	4	3	3	2
sdh3	10	10	3[d]	1	ND
sdh4	7	ND	1	6	ND
Total	818	802	700	403	489

[a] ND indicates no data available;
[b] The cDNA sequence of *ccmFC* mature mRNA was not acquired;
[c] mttB is a pseudogene in *Nelumbo Nucifera*;
[d] Indicates the edit information of either *sdh3a* or *sdh3b;*

Table S8. Positively selected sites in *Nelumbo nucifera* mitochondrial genes and parameter estimates in the branch-site model.

Gene	Parameters under null model	Parameters under alternative model	lnL0	lnL1	2Δl	d.f.	P-value	Positively selected sites
atp1	P0 = 0.73411, ω0 = 0.04850, P1 = 0.10371, (P2a+P2b = 0.16219)	P0 = 0.73409, ω0 = 0.04850, P1 = 0.10371, ω2 = 1.00000, (P2a+P2b = 0.1622)	42287	42287	0	1	1	341 L 0.785
atp4	P0 = 0.58719, ω0 = 0.27211, P1 = 0.20703, (P2a+P2b = 0.20578)	P0 = 0.63816, ω0 = 0.27211, P1 = 0.22500, ω2 = 2.67083, (P2a+P2b = 0.13684)	105268	105269	2	1	0.1573	none
atp6	P0 = 0.84173, ω0 = 0.11281, P1 = 0.15827, (P2a+P2b = 0)	P0 = 0.84173, ω0 = 0.11281, P1 = 0.15827, ω2 = 1.00000, (P2a+P2b = 0)	584570	584570	0	1	1	none
atp8	P0 = 0.49659, ω0 = 0.21112, P1 = 0.28227, (P2a+P2b = 0.22115)	P0 = 0.50674, ω0 = 0.21112, P1 = 0.28804, ω2 = 2.82118, (P2a+P2b = 0.20523)	196277	196280	6	1	0.01431	none
atp9	P0 = 0.33256, ω0 = 0.04649, P1 = 0.05655, (P2a+P2b = 0.61089)	P0 = 0.33255, ω0 = 0.04649, P1 = 0.05655, ω2 = 1.00000, (P2a+P2b = 0.6109)	283609	283609	0	1	1	27 L 0.910
ccmB	P0 = 0.41300, ω0 = 0.21071, P1 = 0.37844, (P2a+P2b = 0.20857)	P0 = 0.41715, ω0 = 0.21071, P1 = 0.38224, ω2 = 2.57537, (P2a+P2b = 0.20061)	705727	705732	10	1	0.001565	none
ccmC	P0 = 0.62823, ω0 = 0.15352, P1 = 0.37177, (P2a+P2b = 0)	P0 = 0.62823, ω0 = 0.15352, P1 = 0.37177, ω2 = 1.00000, (P2a+P2b = 0)	89901	89901	0	1	1	none
ccmFC	P0 = 0.00011, ω0 = 0.20196, P1 = 0.00011, (P2a+P2b = 0.99978)	P0 = 0.00003, ω0 = 0.20196, P1 = 0.00002, ω2 = 1.00000, (P2a+P2b = 0.99995)	630380	630379	2	1	0.1573	101 V 0.685;182 Y 0.601
ccmFN	P0 = 0.47694, ω0 = 0.07410, P1 = 0.52306, (P2a+P2b = 0)	P0 = 0.47694, ω0 = 0.07410, P1 = 0.52306, ω2 = 1.00000, (P2a+P2b = 0)	71409	71409	0	1	1	none
cob	P0 = 0.85629, ω0 = 0.08859, P1 = 0.09391, (P2a+P2b = 0.0498)	P0 = 0.85650, ω0 = 0.08859, P1 = 0.09393, ω2 = 1.00000, (P2a+P2b = 0.04957)	499962	499961	2	1	0.1573	none
cox1	P0 = 0.86770, ω0 = 0.05271, P1 = 0.03245, (P2a+P2b = 0.09986)	P0 = 0.95686, ω0 = 0.05270, P1 = 0.03580, ω2 = 36.05381, (P2a+P2b = 0.00733)	155053	710116	1110126	1	0	36 F 0.996**; 268 Y 0.778
cox2	P0 = 0.87395, ω0 = 0.08431, P1 = 0.07105, (P2a+P2b = 0.05499)	P0 = 0.91075, ω0 = 0.08428, P1 = 0.07367, ω2 = 5.22707, (P2a+P2b = 0.01558)	854782	835539	38486	1	0	117 H 0.681
cox3	P0 = 0.69291, ω0 = 0.06991, P1 = 0.15197, (P2a+P2b = 0.15512)	P0 = 0.71281, ω0 = 0.06991, P1 = 0.15634, ω2 = 3.39625, (P2a+P2b = 0.13085)	409672	409689	34	1	5.511E-09	none

Table S8. (continued)

matR	P0 = 0.53767, ω0 = 0.25209, P1 = 0.46233, (P2a+P2b = 0)	P0 = 0.52719, ω0 = 0.25399, P1 = 0.46538, ω2 = 999.00000, (P2a+P2b = 0.00742)	616124	231991	768266	1	0	138 G 0.549;162 L 0.937
mttB	P0 = 0.62644, ω0 = 0.22775, P1 = 0.37355, (P2a+P2b = 0)	P0 = 0.00000, ω0 = 0.22888, P1 = 0.00000, ω2 = 69.56743, (P2a+P2b = 1)	997233	738385	517696	1	0	76 I 0.735
nad1	P0 = 0.85867, ω0 = 0.08020, P1 = 0.14133, (P2a+P2b = 0)	P0 = 0.85867, ω0 = 0.08020, P1 = 0.14133, ω2 = 1.00000, (P2a+P2b = 0)	556740	556739	2	1	0.1573	none
nad2	P0 = 0.85736, ω0 = 0.13673, P1 = 0.14264, (P2a+P2b = 0)	P0 = 0.85736, ω0 = 0.13673, P1 = 0.14264, ω2 = 1.00000, (P2a+P2b = 0)	254076	254076	0	1	1	none
nad3	P0 = 0.80260, ω0 = 0.09334, P1 = 0.19740, (P2a+P2b = 0)	P0 = 0.80260, ω0 = 0.09334, P1 = 0.19740, ω2 = 1.00000, (P2a+P2b = 0)	754754	754718	72	1	0	none
nad4	P0 = 0.63805, ω0 = 0.10560, P1 = 0.12036, (P2a+P2b = 0.24159)	P0 = 0.79957, ω0 = 0.10557, P1 = 0.15085, ω2 = 8.14262, (P2a+P2b = 0.04958)	682983	444067	477832	1	0	101 F 0.666;282 Y 0.565
nad4L	P0 = 0.88082, ω0 = 0.05944, P1 = 0.11918, (P2a+P2b = 0)	P0 = 0.88082, ω0 = 0.05944, P1 = 0.11918, ω2 = 1.00000, (P2a+P2b = 0)	211305	211305	0	1	1	none
nad5	P0 = 0.82512, ω0 = 0.07890, P1 = 0.07877, (P2a+P2b = 0.09612)	P0 = 0.80601, ω0 = 0.07890, P1 = 0.07694, ω2 = 3.43304, (P2a+P2b = 0.11705)	614042	613569	946	1	0	none
nad6	P0 = 0.84264, ω0 = 0.11946, P1 = 0.15736, (P2a+P2b = 0)	P0 = 0.84264, ω0 = 0.11946, P1 = 0.15736, ω2 = 1.00000, (P2a+P2b = 0)	774622	774622	0	1	1	none
nad7	P0 = 0.75540, ω0 = 0.04222, P1 = 0.11002, (P2a+P2b = 0.13459)	P0 = 0.86195, ω0 = 0.04221, P1 = 0.12556, ω2 = 14.34779, (P2a+P2b = 0.01249)	207648	137869	139558	1	0	80 L 0.576;107 F 0.741
nad9	P0 = 0.62737, ω0 = 0.09905, P1 = 0.23389, (P2a+P2b = 0.13874)	P0 = 0.68082, ω0 = 0.09903, P1 = 0.25383, ω2 = 2.71673, (P2a+P2b = 0.06535)	33049	998902	1931706	1	0	132 Y 0.638
rpl10	P0 = 0.57106, ω0 = 0.17835, P1 = 0.42894, (P2a+P2b = 0)	P0 = 0.57105, ω0 = 0.17835, P1 = 0.42895, ω2 = 1.00000, (P2a+P2b = 0)	7138	7138	0	1	1	none
rpl16	P0 = 0.55834, ω0 = 0.09571, P1 = 0.23094, (P2a+P2b = 0.21072)	P0 = 0.56564, ω0 = 0.09571, P1 = 0.23396, ω2 = 2.65551, (P2a+P2b = 0.20041)	843775	843776	2	1	0.1573	none
rpl2	P0 = 0.00000, ω0 = 0.15937, P1 = 0.00000, (P2a+P2b = 1)	P0 = 0.00000, ω0 = 0.15937, P1 = 0.00000, ω2 = 1.00000, (P2a+P2b = 1)	829899	829899	0	1	1	4 L 0.823

Table S8. (continued)

rpl5	P0 = 0.56464, ω0 = 0.17610, P1 = 0.37452, (P2a+P2b = 0.06084)	P0 = 0.47265, ω0 = 0.17610, P1 = 0.31351, ω2 = 2.96351, (P2a+P2b = 0.21384)	54906	54918	24	1	9.634E-07	none
rps1	P0 = 0.55354, ω0 = 0.18225, P1 = 0.27408, (P2a+P2b = 0.17239)	P0 = 0.53602, ω0 = 0.18225, P1 = 0.26540, ω2 = 3.01608, (P2a+P2b = 0.19858)	348314	348313	2	1	0.1573	none
rps10	P0 = 0.35619, ω0 = 0.05030, P1 = 0.44551, (P2a+P2b = 0.1983)	P0 = 0.34477, ω0 = 0.05030, P1 = 0.43122, ω2 = 3.26842, (P2a+P2b = 0.22401)	307202	307201	2	1	0.1573	none
rps11	P0 = 0.44281, ω0 = 0.10511, P1 = 0.12116, (P2a+P2b = 0.43603)	P0 = 0.75952, ω0 = 0.10520, P1 = 0.20895, ω2 = 92.52504, (P2a+P2b = 0.03153)	736647	212111	1049072	1	0	1 K 0.765;74 G 0.831;76 G 0.837
rps12	P0 = 0.58893, ω0 = 0.05371, P1 = 0.21901, (P2a+P2b = 0.19205)	P0 = 0.58100, ω0 = 0.05371, P1 = 0.21607, ω2 = 2.64459, (P2a+P2b = 0.20293)	246084	246090	12	1	0.000532	none
rps13	P0 = 0.87942, ω0 = 0.17068, P1 = 0.12058, (P2a+P2b = 0)	P0 = 0.87942, ω0 = 0.17068, P1 = 0.12058, ω2 = 1.00000, (P2a+P2b = 0)	596898	596896	4	1	0.0455	none
rps14	P0 = 0.60801, ω0 = 0.17363, P1 = 0.23402, (P2a+P2b = 0.15797)	P0 = 0.58688, ω0 = 0.17363, P1 = 0.22588, ω2 = 3.08103, (P2a+P2b = 0.18724)	664893	664896	6	1	0.01431	none
rps19	P0 = 0.76556, ω0 = 0.08044, P1 = 0.23444, (P2a+P2b = 0)	P0 = 0.76556, ω0 = 0.08044, P1 = 0.23444, ω2 = 1.00000, (P2a+P2b = 0)	593877	593877	0	1	1	none
rps2	P0 = 0.76802, ω0 = 0.20018, P1 = 0.23198, (P2a+P2b = 0)	P0 = 0.76802, ω0 = 0.20018, P1 = 0.23198, ω2 = 1.00000, (P2a+P2b = 0)	309356	309356	0	1	1	none
rps3	P0 = 0.69822, ω0 = 0.13708, P1 = 0.30178, (P2a+P2b = 0)	P0 = 0.69823, ω0 = 0.13708, P1 = 0.30177, ω2 = 1.00000, (P2a+P2b = 0)	540546	540544	4	1	0.0455	none
rps4	P0 = 0.00000, ω0 = 0.00000, P1 = 1.00000, (P2a+P2b = 0)	P0 = 0.00000, ω0 = 0.00000, P1 = 1.00000, ω2 = 2.65795, (P2a+P2b = 0)	724034	724034	0	1	1	none
rps7	P0 = 0.83338, ω0 = 0.19230, P1 = 0.16662, (P2a+P2b = 0)	P0 = 0.83338, ω0 = 0.19230, P1 = 0.16662, ω2 = 1.00000, (P2a+P2b = 0)	476124	476124	0	1	1	none
sdh3	P0 = 0.59158, ω0 = 0.12753, P1 = 0.36021, (P2a+P2b = 0.0482)	P0 = 0.60975, ω0 = 0.12715, P1 = 0.37332, ω2 = 999.00000, (P2a+P2b = 0.01693)	613911	951482	675142	1	0	35 M 0.670;83 N 0.544
sdh4	P0 = 0.00000, ω0 = 0.21987, P1 = 0.00000, (P2a+P2b = 1)	P0 = 0.00000, ω0 = 0.21998, P1 = 0.00000, ω2 = 999.00000, (P2a+P2b = 1)	427328	517181	179706	1	0	25 I 0.961*;31 L 0.956*

Table S9. The homologs in 78 species of chloroplast-derived fragments in *Nelumbo nucifera* mtDNA.

**Position**	469237-471234	523411-524797	516533-517761	451200-452196	471275-471895	230963-231283	452532-452828	181488-181698	29058-29263	318795-319000	523258-523402	472035-472175	285719-285805	426801-426884	505480-505560	123420-123497	413440-413496	91514-91569	342137-342190
**Genes Contained**	**petN psbM**	ycf3	petL petG **trnW(CCA) trnP(TGG)**	psbC	**trnD(GTC)**	psbC	psbD psbC	ndhD	ycf2	ycf2	ycf3	**trnT(GGT)**	ycf2	**trnN(GTT)**	**trnH(GTG)**	**trnM(CAT)**	ndhE	trnT(GGT)	trnA(TGC)
**Length(bp)** / **SP_name**	1998	1387	1229	997	621	321	297	211	206	206	145	141	87	84	81	78	57	56	54
Brassica rapa subsp. campestri	0	0	0	0	0	0	0	0	0	0	0	0	0	1	1	1	0	0	0
Brassica juncea	0	0	0	0	0	0	0	0	0	0	0	0	0	1	1	1	0	0	0
Brassica oleracea	0	0	0	0	0	0	0	0	0	0	0	0	0	1	1	1	0	0	0
Brassica napus	0	0	0	0	0	0	0	0	0	0	0	0	0	1	1	1	0	0	0
Brassica carinata	0	0	0	0	0	0	0	0	0	0	0	0	0	1	1	1	0	0	0
Raphanus sativus	0	0	0	0	0	0	0	0	0	0	0	0	0	1	1	1	0	0	0
Arabidopsis thaliana	0	0	0	0	0	0	0	0	0	0	0	0	0	1	1	1	0	0	0
Batis maritima	0	0	0	0	0	0	0	0	0	0	0	0	0	1	1	1	0	0	0
Carica papaya	0	0	0	0	1	0	0	1	1	1	0	0	0	1	1	1	0	0	0
Ricinus communis	0	1	0	0	0	0	0	0	0	0	1	0	1	1	1	1	0	0	0
Vigna radiata	0	0	0	0	0	0	0	0	0	0	0	0	1	1	1	1	0	0	0
Vigna angularis	0	0	0	0	0	0	0	0	0	0	0	0	1	1	1	1	0	0	0
Glycine max	0	0	0	0	0	0	0	0	0	0	0	0	1	1	1	1	0	0	0
Millettia pinnata	0	0	0	0	0	0	0	0	0	0	0	0	0	1	1	1	0	0	0
Lotus japonicus	0	0	0	0	0	0	0	0	0	0	0	0	1	1	1	1	0	0	0
Cucurbita pepo	0	0	0	0	0	0	0	1	1	1	1	0	1	1	1	1	0	0	0
Cucumis sativus	0	0	0	0	0	0	0	0	1	1	0	0	0	0	1	1	0	0	0
Citrullus lanatus	0	0	0	0	0	0	0	0	0	0	0	0	1	1	1	1	0	0	0
Malus x domestica	0	0	0	0	0	0	0	0	0	0	0	0	0	1	1	1	0	0	0
Salvia miltiorrhiza	0	0	0	0	0	0	1	0	1	1	0	0	0	1	1	1	0	0	0
Ajuga reptans	0	0	0	0	0	0	0	0	0	0	0	0	0	1	0	1	0	0	0
Mimulus guttatus	0	0	0	0	0	0	0	0	0	0	0	0	1	1	1	1	0	0	0
Boea hygrometrica	0	0	0	1	0	1	1	0	1	1	0	0	0	1	1	1	0	0	0
Daucus carota subsp. sativus	0	0	0	0	0	0	0	0	0	0	0	0	1	1	1	0	0	0	0
Helianthus annuus	0	0	0	0	0	0	0	0	0	0	0	0	1	1	1	1	0	0	0
Asclepias syriaca	0	0	0	0	0	0	0	0	0	0	0	0	0	1	1	1	0	0	0
Rhazya stricta	0	1	0	0	0	0	0	0	0	0	1	0	0	1	1	0	0	0	0
Nicotiana tabacum	0	0	1	0	0	0	0	0	0	0	0	0	1	1	1	1	0	0	0
Vaccinium macrocarpon	0	0	0	0	0	0	0	1	0	0	0	0	1	1	0	1	0	0	0
Beta vulgaris subsp. vulgaris	0	0	0	0	0	0	0	0	0	0	0	0	0	1	1	1	0	0	0
Beta vulgaris subsp. maritima	0	0	0	0	0	0	0	0	0	0	0	0	0	1	1	1	0	0	0
Beta macrocarpa	0	0	0	0	0	0	0	0	0	0	0	0	0	1	1	1	0	0	0
Silene vulgaris	0	0	0	1	0	0	1	0	0	0	0	0	0	1	0	0	0	0	0
Silene latifolia	0	0	0	0	0	0	0	0	0	0	0	0	0	1	1	0	0	0	0
Vitis vinifera	0	0	1	1	0	1	1	0	0	0	1	0	1	1	1	1	0	0	0
Zea luxurians	0	0	0	0	0	0	0	0	0	0	0	0	0	1	1	1	0	0	0
Zea perennis	0	0	0	0	0	0	0	0	0	0	0	0	0	1	1	1	0	0	0
Zea mays subsp. mays	0	0	0	0	0	0	0	0	0	0	0	0	0	1	1	1	0	0	0
Zea mays subsp. parviglumis	0	0	0	0	0	0	0	0	0	0	0	0	0	1	1	1	0	0	0
Tripsacum dactyloides	0	0	0	0	0	0	0	0	0	0	0	0	0	1	1	1	0	0	0
Sorghum bicolor	0	0	0	0	0	0	0	0	0	0	0	0	0	1	1	1	0	0	0
Oryza sativa Japonica Group	0	0	0	0	0	0	0	0	0	0	0	0	0	1	1	1	0	0	0
Oryza rufipogon	0	0	0	0	0	0	0	0	0	0	0	0	0	1	1	1	0	0	0
Oryza sativa Indica Group	0	0	0	0	0	0	0	0	0	0	0	0	0	1	1	1	0	0	0
Triticum timopheevii	0	0	0	0	0	0	0	0	0	0	0	0	0	1	0	1	0	0	0
Triticum aestivum	0	0	0	0	0	0	0	0	0	0	0	0	0	1	0	1	0	0	0
Aegilops speltoides	0	0	0	0	0	0	0	0	0	0	0	0	0	1	0	1	0	0	0
Phoenix dactylifera	0	0	0	0	0	0	0	0	0	0	0	0	0	1	1	1	0	0	0
Butomus umbellatus	0	0	0	0	0	0	0	0	0	0	0	0	0	0	1	0	0	0	0
Spirodela polyrhiza	0	0	0	1	0	0	0	0	0	0	0	0	0	1	1	1	0	0	0
Liriodendron tulipifera	1	0	1	1	1	1	1	1	0	0	0	0	1	1	1	1	0	0	0
Amborella trichopoda	0	0	0	1	0	1	1	1	0	1	1	0	0	1	1	1	1	0	0
Cycas taitungensis	0	0	0	0	0	0	0	0	0	0	0	0	0	0	1	1	0	0	0
Huperzia squarrosa	0	0	0	0	0	0	0	0	0	0	0	0	0	0	0	0	0	0	0
Pleurozia purpurea	0	0	0	0	0	0	0	0	0	0	0	0	0	0	0	0	0	0	0
Anomodon rugelii	0	0	0	0	0	0	0	0	0	0	0	0	0	0	0	0	0	0	0
Hypnum imponens	0	0	0	0	0	0	0	0	0	0	0	0	0	0	0	0	0	0	0
Climacium americanum	0	0	0	0	0	0	0	0	0	0	0	0	0	0	0	0	0	0	0
Ptychomnion cygnisetum	0	0	0	0	0	0	0	0	0	0	0	0	0	0	0	0	0	0	0
Ulota hutchinsiae	0	0	0	0	0	0	0	0	0	0	0	0	0	0	0	0	0	0	0
Orthotrichum stellatum	0	0	0	0	0	0	0	0	0	0	0	0	0	0	0	0	0	0	0
Bartramia pomiformis	0	0	0	0	0	0	0	0	0	0	0	0	0	0	0	0	0	0	0
Physcomitrella patens	0	0	0	0	0	0	0	0	0	0	0	0	0	0	0	0	0	0	0
Funaria hygrometrica	0	0	0	0	0	0	0	0	0	0	0	0	0	0	0	0	0	0	0
Buxbaumia aphylla	0	0	0	0	0	0	0	0	0	0	0	0	0	0	0	0	0	0	0
Atrichum angustatum	0	0	0	0	0	0	0	0	0	0	0	0	0	0	0	0	0	0	0
Tetraphis pellucida	0	0	0	0	0	0	0	0	0	0	0	0	0	0	0	0	0	0	0
Sphagnum palustre	0	0	0	0	0	0	0	0	0	0	0	0	0	0	0	0	0	0	0
Phaeoceros laevis	0	0	0	0	0	0	0	0	0	0	0	0	0	0	0	0	0	0	0
Marchantia polymorpha	0	0	0	0	0	0	0	0	0	0	0	0	0	0	0	0	0	0	0
Treubia lacunosa	0	0	0	0	0	0	0	0	0	0	0	0	0	0	0	0	0	0	0
Nitella hyalina	0	0	0	0	0	0	0	0	0	0	0	0	0	0	0	0	0	0	0
Chara vulgaris	0	0	0	0	0	0	0	0	0	0	0	0	0	0	0	0	0	0	0
Roya obtusa	0	0	0	0	0	0	0	0	0	0	0	0	0	0	0	0	0	0	0
Chaetosphaeridium globosum	0	0	0	0	0	0	0	0	0	0	0	0	0	0	0	0	0	0	0
Entransia fimbriata	0	0	0	0	0	0	0	0	0	0	0	0	0	0	0	0	0	0	0
Chlorokybus atmophyticus	0	0	0	0	0	0	0	0	0	0	0	0	0	0	0	0	0	0	0
Mesostigma viride	0	0	0	0	0	0	0	0	0	0	0	0	0	0	0	0	0	0	0

*Blocks numbered as “1” and marked as green were homologs of chloroplast-derived fragments in Nelumbo nucifera mtDNA.

Table S10. The primers used to amplify the cDNA of the protein coding genes of the *N. nucifera* mitochondrial genome.

Gene	Forward Primer	Reverse Primer	Predicted Product sizes
matR	GAAGTTTAGACCGCTCACA	AGCAGACAACTAACGCAAGC	2096
nad5	TTTGGTTGTCTGATTACA	CTCCTCCAGTTCGATTAT	2057
ccmFN	CAGAAATTTTTAAATGAGCACG	AACCAGCCCAGCAATAAG	1777
rps3	AAAAGTAAAGTCTAAGCG	ACTACATCTGCCTTTACG	1738
cox1	AGGAAACGAAATAATCTCA	TTAGGTTCTTAGTAGCAGTC	1645
atp1	CCCTTGACTCTATCCTATT	AGTCCACATTATTCCTTTC	1577
nad4	TTCAATGTTCGATTCTACT	TTCTTTGCTGATTCCTCT	1526
nad2	GCAGAATTAGTTCGGATC	ACCTTGTCAATGATCGAA	1512
ccmFC	AATCGAACGGATAGAGCA	CGCAGCCACTACTTTGAC	1398
cob	CAAGAGTTGTCACGATAG	TGGTGTAATACTCACTCG	1259
nad7	ATTTCTGCCTTTCTTTCC	TCGAGCAACTAGTAGTCC	1221
rps4	TACGCGATGTGGCGAAAA	GTCCCTATGAGCGACTAC	1114
rpl2	TCGAATGATTACGAGATACA	CTGTGTGAACAAGGTCTTG	1037
nad1	TGAGTGAATAGAAAATCG	ACTTCCTGGCACATACAT	1016
mttBψ[1]	CAATCCTATTTGTTTTGTTC	GTGACTTTGCCAGGTTCT	896
cox3	ACAACCGAGGCAAAGTGG	TCCACTGATTCGTTCCTT	836
cox2	AAGAATGAACCAAACCAA	TTTTCCCTCACCCTACTC	831
atp6	GGGATTAAATGAAAAGTG	CGCTTTTGTTCAATTATA	830
ccmC	CCATGTCTTTCTTGTTCACTAG	GAGAATCAAGCAACTTGCC	811
rps2	TAACCACCTGAGCCAACT	CCCCTAGCTTGGTATTTG	734
nad6	TTTCAGGGAAGGACGACG	GAGCGAGTGTCCCCTGGT	672
ccmB	GGAACGAAGAAGTAAGGA	AGGCTTTTCAATGACTTG	663
rps1	GAAGAGGAGGAATACAAA	TGTTTGATCTGCCGCTGT	652
atp4	ATGCTGATAACGTTTCTAA	TAGCATGAACATGAATTAGAT	638
nad9	GCGGAACTACAAGAAAGC	CTGGACCTATTCTGATTCTG	626
rpl5	GGAAAAGAAAAGCCAACT	TTCGCTTCTCCGACATCT	597
rpl16	TGTATTTAACCAGAAAATAG	TCCCCACTAACCAATTAC	558
rpl10	TTCTTTGAAGATCGAGGA	CTTCCCGACCTATTTTAC	553
atp8	TCTGGAGGGAATCATTTT	CCGAACGACCAAGTCATA	535
sdh4	CATCACGTTGGCTTTGAA	TCTCAGTCTCAGCCTCCA	496
rps7	TTGCTCGGAAATTCTTAG	GCTCTTTATGTGGTCTCA	489
rps11	GGGCAGGAGTTTCAAATC	GGAGAACTTCTTGGAACT	483
rps10	CCAAAAAGAAACACCACC	TTTGATCCCCTTTTCCTC	424
rps12	GAAGACAAAGGAAAGAGC	AACAAGTTCAAGAGGCAT	422
sdh3a[2]	TGAGCATCCATCAACAAC	GAGAAGCACAAGCAGTGA	419
nad3	AGAACGAAGTGGGCTGTG	TTTTGCCCTATCACTAGT	398
rps13	TAGTCCAAGGAAGGATCA	TACCAAGGGCTTTCGGTA	398
sdh3b[2]	GAACATCCATCAACAACA	TACAGTCATTTCCATCTT	360
nad4L	CTCTTACATTCCACGTTC	CTTCGTATTTTGTAACTC	358
rps14	GATGAGGGGGAAACTCAGT	CCTTGTTCTATTGGTTTGG	350
rps19	GGGAAACGCAGAAATTAG	GCCATATGCGCTTAGACT	324
atp9	GGAAAAGCGTGACGAGAA	AGAGGTTAAAATCAAAACT	284

[1] *mttBψ* represents a pseudogene.

[2] *sdh3a* and *sdh3b* are two different copies of gene *sdh3*.

Table S11. Accession numbers of the species used in the present study.

Species	Accession No.	Species	Accession
Aegilops speltoides	NC_022666.1	*Marchantia polymorpha*	NC_001660.1
Ajuga reptans	NC_023103.1	*Mesostigma viride*	NC_008240.1
Amborella trichopoda	KF754799-KF754803	*Millettia pinnata*	NC_016742.1
Anomodon rugelii	NC_016121.1	*Mimulus guttatus*	NC_018041.1
Arabidopsis thaliana	NC_001284.2	*Nicotiana tabacum*	NC_006581.1
Asclepias syriaca	NC_022796.1	*Nitella hyalina*	NC_017598.1
Atrichum angustatum	NC_024520.1	*Orthotrichum stellatum*	NC_024522.1
Bartramia pomiformis	NC_024519.1	*Oryza rufipogon*	NC_013816.1
Batis maritima	NC_024429.1	*Oryza sativa Indica Group*	NC_007886.1
Beta macrocarpa	NC_015994.1	*Oryza sativa Japonica*	NC_011033.1
Beta vulgaris subsp. maritima	NC_015099.1	*Phaeoceros laevis*	NC_013765.1
Beta vulgaris subsp. vulgaris	NC_002511.2	*Phoenix dactylifera*	NC_016740.1
Boea hygrometrica	NC_016741.1	*Physcomitrella patens*	NC_007945.1
Brassica carinata	NC_016120.1	*Pleurozia purpurea*	NC_013444.1
Brassica juncea	NC_016123.1	*Ptychomnion cygnisetum*	NC_024514.1
Brassica napus	NC_008285.1	*Raphanus sativus*	NC_018551.1
Brassica oleracea	NC_016118.1	*Rhazya stricta*	NC_024293.1
Brassica rapa subsp. campestris	NC_016125.1	*Ricinus communis*	NC_015141.1
Butomus umbellatus	NC_021399.1	*Roya obtusa*	NC_022863.1
Buxbaumia aphylla	NC_024518.1	*Salvia miltiorrhiza*	NC_023209.1
Carica papaya	NC_012116.1	*Silene latifolia*	NC_014487.1
Chaetosphaeridium globosum	NC_004118.1	*Silene vulgaris*	NC_016406.1
Chara vulgaris	NC_005255.1	*Sorghum bicolor*	NC_008360.1
Chlorokybus atmophyticus	NC_009630.1	*Sphagnum palustre*	NC_024521.1
Citrullus lanatus	NC_014043.1	*Spirodela polyrhiza*	NC_017840.1
Climacium americanum	NC_024515.1	*Tetraphis pellucida*	NC_024290.1
Cucumis sativus	NC_016005.1	*Treubia lacunosa*	NC_016122.1
Cucurbita pepo	NC_014050.1	*Tripsacum dactyloides*	NC_008362.1
Cycas taitungensis	NC_010303.1	*Triticum aestivum*	NC_007579.1
Daucus carota subsp. sativus	NC_017855.1	*Triticum timopheevii*	NC_022714.1
Entransia fimbriata	NC_022861.1	*Ulota hutchinsiae*	NC_024517.1
Funaria hygrometrica	NC_024523.1	*Vaccinium macrocarpon*	NC_023338.1
Glycine max	NC_020455.1	*Vigna angularis*	NC_021092.1
Helianthus annuus	NC_023337.1	*Vigna radiata*	NC_015121.1
Huperzia squarrosa	NC_017755.1	*Vitis vinifera*	NC_012119.1
Hypnum imponens	NC_024516.1	*Zea luxurians*	NC_008333.1
Liriodendron tulipifera	NC_021152.1	*Zea mays subsp. mays*	NC_007982.1
Lotus japonicus	NC_016743.2	*Zea mays subsp.*	NC_008332.1
Malus x domestica	NC_018554.1	*Zea perennis*	NC_008331.1

Method S1. The method for isolating the *N. nucifera* mitochondrial genome.

The round ends of the mature *N. nucifera* seed shell were cut open carefully in the premise of embryo intactness. Then, the seeds were rinsed with sterile water twice and germinated in a dark environment in the incubator at room temperature. When the green embryos became completely yellow, 250 g leaves were cut and homogenized in a Waring blender (2×10 second pulses and 2×5 second pulses, respectively) in chilled homogenization buffer (0.4 M Sucrose, 50 mM Tris-HCl, 3 mM $EDTA_2Na$, 0.5% BSA, 1% PVP-40. Add 0.5% β-mercaptoethanol immediately before use, pH 7.5) at a 1:4 (w/v) ratio. After filtration through four layers of miracloth, the extract was centrifuged at 1,000 g for 15 min to eliminate large cell fragments. To sediment the mitochondria, the supernatant was recentrifuged at 3,000 g for 15 min to eliminate plastids, and the resulting supernatant was then centrifuged at 18,000 g for 20 min. To purify the mitochondria, the crude mitochondrial pellet was resuspended in wash buffer A (0.4 M Sucrose, 50 mM Tris-HCl, pH7.5), adding $MgCl_2$ and DNase I to final concentrations of 10 mM and 50 μg/ml, separately. The result was mixed gently but well and incubated for 1 h on ice with occasional swirling. The suspension was centrifuged at 18,000 g for 20 min at 4℃. The pellet was resuspended in wash buffer B (0.4 M Sucrose, 50 mM Tris-HCl, 25 mM $EDTA_2Na$, pH 7.5) and layered on a discontinuous sucrose density gradient that consisted of 0.6/1.2/1.6/2.0 M sucrose solutions (pH 7.5) that contained 10 mM $EDTA_2Na$. The gradient was placed in a SW-627 rotor and centrifuged at 80,000 g for 45 min. Purified mitochondria were removed from the 1.6 M-1.2 M interphase and washed with wash buffer B (0.4 M Sucrose, 50 mM Tris-HCl, 25 mM $EDTA_2Na$, pH 7.5) and centrifuged at 18,000 g for 20 min. The pellet was resupended in 2 ml wash buffer B (0.4 M Sucrose, 50 mM Tris-HCl, 25 mM $EDTA_2Na$, pH7.5). One-fifth volume of lysis buffer (5% SDS, 50 mM Tris-HCl, 25 mM $EDTA_2Na$) and Proteinase K (final concentration of 200 μg/ml) were gently added to the mitochondria solutions. Then, the tube was gently inverted and mixed once for every 15 min during a period of 30 min at 37℃, 30 min at 50℃ and 30 min at 65℃ in water bath. After cold NH_4Ac to 0.8 M final concentration was added, the nucleic acids were extracted with an equal volume of Tris-saturated phenol/chloroform/isoamyl alcohol (PCI, 25:24:1) once and chloroform/isoamyl alcohol once (CI, 24:1), separately. The mtDNA were precipitated in 2 volumes of 100% ethanol overnight at -20°C and centrifuged at 18,000 g for 20 min. The crude DNA was washed twice with 70% ethanol and resuspended in TE buffer with RNase digestion for 30 min at 37℃.

We designed three pairs of primers that specifically corresponded to the loci of the nuclear *β-actin* gene (*β-actin*_F: 5'-CCTGATGGGCAAGTGATT-3', *β-actin*_R: 5'-GCTCAT-ACGGTCAGCAATA-3'), the mitochondrial *rps3* (ribosomal protein S3) gene (*rps3*_F: 5'-CAAGCATCCGAAATACGC-3', *rps3*_R: 5'-TACTTCCGCAGGAGCATA-3') and the chloroplast *rbcL* (the ribulose 1, 5-bisphosphate carboxylase) gene (*rbcL*_F: 5'-GCTGCCGAATCTTCTACTGG-3', *rbcL*_R: 5'-CCAAGGGTGTCC TAAAGT-3'). The purity of the mtDNA was evaluated by the semi-quantitative PCR technique with these specific primers.
